# Drivers of floristic discovery in a temperate flora: insights from three decades of vascular plant records in Ukraine

**DOI:** 10.1186/s40529-026-00492-4

**Published:** 2026-03-06

**Authors:** Mykyta Peregrym, Ihor Olshanskyi, Svitlana Zhygalova

**Affiliations:** 1 Ecology and Genetics Research Unit, University of Oulu, P.O. Box 3000, Oulu, 90014 Finland; 2 Department of Landscape Gardening and Ecology, Luhansk Taras Shevchenko National University, Hohol’ Str., 90, Myrhorod, Poltava region 37600 Ukraine; 3 Department of Systematics and Floristics of Vascular Plants, M.G. Kholodny Institute of Botany, National Academy of Sciences of Ukraine, Tereshchenkivska Str., 2, Kyiv, 01004 Ukraine; 4 London, UK

**Keywords:** Biodiversity monitoring, Citizen science, Grasslands, Herbarium collections, Hybrids, Synanthropic habitats

## Abstract

**Background:**

Floristic discoveries continue to occur even in regions with a long history of botanical exploration. Ukraine, one of the largest countries in Europe, has been the subject of intensive botanical study for more than two centuries, yet new species, subspecies, and national records are still regularly documented. Understanding the ecological and methodological factors that drive these discoveries is important for improving biodiversity assessment, guiding field surveys, and updating national floras. This study provides a comprehensive synthesis of all vascular plant taxa newly described for science or newly recorded from Ukraine during the period from 1997 to 2024 and identifies the principal drivers of floristic discovery in a temperate flora.

**Results:**

A total of 331 species and subspecies of vascular plant were discovered or newly recorded during the study period, including 57 taxa described as new to science. These findings span lycopods, ferns, gymnosperms, and flowering plants, with flowering plants contributing the largest share. Synanthropic habitats, particularly those associated with human disturbance and escaped cultivated plants, yielded the highest number of discoveries. Grasslands, woodlands, stone outcrops, and coastal habitats also contributed substantially, while mountainous areas were notable centres for both newly described taxa and hybridogenic diversity. The majority of discoveries were based on material collected during spring and summer, although historical herbarium specimens, some over a century old, were essential for many taxonomic descriptions and for confirming previously overlooked taxa. Citizen science platforms supported several recent national records by enabling rapid detection and preliminary verification of unusual occurrences, although they have not yet contributed directly to the description of new taxa.

**Conclusions:**

Our findings highlight the continued incompleteness of floristic knowledge, even in well-studied temperate regions, and underscore the need for targeted survey strategies that integrate historical collections, underexplored habitats, and public participation. While Ukraine provides the case study, these patterns and methodological approaches are broadly applicable to biodiversity assessment and conservation planning in similar biogeographic contexts worldwide.

**Supplementary Information:**

The online version contains supplementary material available at 10.1186/s40529-026-00492-4.

## Background

The discovery of a new species is one of the greatest thrills of the life botanical, often shrouded in romantic stories (Baker 2014). A chance to find something absolutely new motivates young people to become a botanist, and already experienced botanists to get the recognition. At the same time the facts of descriptions of species new for science are honored even for well-known and leading scientific organisations. That is why they inform about their successes in biodiversity discovery almost every year on their official websites. So, from ancient dinosaurs to worms at the bottom of the ocean, scientists and associates from the Natural History Museum (London, UK) described an extraordinary 815 new species in 2023 (Davis 2023). The researchers of the California Academy of Sciences added 153 new species to the tree of life in that time, from observing to collecting to DNA testing (Lindqwister 2023). In 2023, 74 plants and 15 fungi were named by botanists and mycologists at Kew and at their partner organisations around the globe (Cheek 2024). The Missouri Botanical Garden’s science and conservation staff discovered and named 67 newly-described and newly-resurrected species of plants during the last year (Martin 2023). This global enthusiasm has even led to the development of strategic guidelines and predictive maps for finding new species (Šlapeta 2013; Pennisi 2021; Martin and Haelewaters 2023).

Generally, ca. 2000 new species of plants are described around the world every year, however, the rate of discovery is slowing down, due to reduction in financial and scientific support for fundamental natural history studies (Christenhusz and Byng 2016). For example, average of 19.5 species of vascular plants described per year in Nigeria (the long-term trend in species descriptions credibly declines over time), nevertheless predictions for the number of new species descriptions by 2070 vary from 1004 to 2239 species for this country (Bello et al. 2023). In Europe, where floras have been intensively surveyed for centuries, the description of new taxa is rare. Yet, even documenting a species as new to a national flora remains valuable, with implications for conservation, invasion biology, and biogeographic knowledge. Understanding the ecological and methodological drivers behind such discoveries can improve survey efficiency and biodiversity monitoring, especially in regions considered well-explored. As well, the process impacts directly on biodiversity conservation and our knowledge about evolution (Hey 2009).

Ukraine provides a good case study: it is the largest country wholly within Europe, with botanical research dating back to the late 18^th^ century (Melnyk 2012) and a rich history of national and regional floristic works (“Flora of the Ukrainian SSR”, Vol. I-XII (1936–1965); “Flora Unionis Rerumpublicarum Sovieticarum Socialisticarum”, Vol. I-XXX (1934–1964); “Flora of the European part of the USSR”, Vol. I-VIII (1974–1989); two editions of the checklist of vascular plants of the former USSR (Cherepanov 1995, 2007); and many regional floras, cheklists, identification guides). The most recent comprehensive checklist of vascular plants (Mosyakin and Fedoronchuk 1999) is now over 25 years old, and numerous new species, subspecies, and hybrids have been reported since its publication. Here, we compile and analyze a list of all new species and subspecies of vascular plant for science and for Ukraine recorded for the first time from the country between 1997 and 2024, identifying patterns in taxonomy, origin, habitat, geography, seasonality, and data sources. Such a review is carried in the first time for the flora of Ukraine, and at the same time these results are used to infer general drivers of floristic discovery in temperate regions and to provide recommendations applicable beyond the country. As well, such information will be useful for updating data in floristic lists of continental, national and regional levels, as well as it will allow researchers to be more effective and targeted to carry out their explorations.

## Materials and methods

### Study area

As mentioned above, our research covers the territory of Ukraine, a country geographically located in Central Europe, but politically – in Eastern Europe. The total area of the internationally recognised territory of the country is 603,549 km^2^ (Rudenko 2007; Zasenko et al. 2024). It lies in a temperate climatic zone influenced by moderately warm, humid air from the Atlantic Ocean (Rudenko 2007). Average annual temperatures range from about 5.5–7 °C in the north to about 11–13 °C in the south (Zasenko et al. 2024). There are three main zones of natural vegetation (the forest zone, the forest-steppe, and the steppe) and two mountain countries with a clearly defined altitudinal zone (the Crimean Mountains and the Ukrainian part of the Carpathians) (Rudenko 2007). The highest peak of the Ukrainian Carpathians is the Hoverla Mount (2,061 m above sea level), and the Roman-Kosh Mount (1,545 m above sea level) is the highest peak of the Crimean Mountains (Rudenko 2007; Zasenko et al. 2024). However, natural vegetation is highly transformed by antropogenic impact, for example steppes occupied about 40% of the recognized territory of the country, but presently only about 1–3% of the natural and semi natural steppes of Ukraine remain unchanged (not transformed) (Ya. Didukh 2009; Korotchenko and M. Peregrym 2012).

The total number of vascular plants within Ukraine has not been established clearly yet. There is different data. For example, Mosyakin and Fedoronchuk mentioned 5100 species based on the National Report, and they noted “our estimation is somewhat different”, but the accurate number is absent (Mosyakin and Fedoronchuk 1999). According to Ya. Didukh, the spontaneous flora of vascular plants of Ukraine consists of almost 4500 species (Didukh 2010). Unfortunately, more precise information is not available.

### Terminology

The term “floristic discovery” is used in this study that might have a wider meaning from the description of a new plant taxon for science to a finding of any new plant taxon, even the lowest rank, in any area, even the smallest one (for example, within a yard, a village, a town or a district). However, in our case we narrowed it to floristic findings of new species and subspecies of vascular plants for the internationally recognised territory of Ukraine, as well as descriptions of new species and subspecies of vascular plants for science from the territory studied.

### Literature review

The research is based exclusively on the results of a literature review using Scopus (https://www.scopus.com), Web of Science (https://www.webofscience.com) and PubMed (https://pubmed.ncbi.nlm.nih.gov) databases, as well as not indexed periodical editions and monographs published in Ukraine and Russia mostly in Slavic languages in the period 1997 – December 31, 2024. A part of publications of 1997 and 1998 also were included in our research, but only unaccounted ones in the checklist by Mosyakin and Fedorchuk (Mosyakin and Fedoronchuk 1999). A total of 192 publications were analyzed in our study (Petryk 1992; Shipunov 1996, 1997, 2000; Rostanski et al. 1997; Yeremko 1997; Agapova 1998; Egorova 1998; Krassovskaya 1998; Levichev 1998, 2008; Moysienko 1998, 2005; Travnicek 1998; Kirschner and Štěpánek 1998; Mosyakin and Moysienko 1999; Honcharenko 2000; Tzvelev 2000, 2001a, b, 2002, 2003a, b, 2004, 2005, 2006, 2007, 2009, 2012; Umanets 2000a, 2000b Vasyljeva and Kovalenko 2000; Danylyk and Panchenko 2001; Krassovskaja et al. 2001; Nikitin 2001, 2002, 2003; Pavlov 2001; Didukh and Boratynski 2002; Shumilova 2002, 2014; Sova and Mosyakin 2002; Tikhomirov 2002, 2015; Byalt and Orlova 2003; Dubyna et al. 2003; Kuziarin 2003, 2009, 2012; Tatanov 2003a, b; Romo et al. 2004; Seregin 2004, 2008, 2009, 2010, 2012; Thomson 2004; Zielinski 2004; Kucherevskyi 2005; Panchenko and Mosyakin 2005; Chorna et al. 2006; Moysienko and Yena 2006; Yena et al. 2006, 2011; Danylyk et al. 2007; Albach 2008; Gureyeva and Page 2008; Krassovskaja 2008; Moysienko and Mosyakin 2008; Orlov 2008; Yena 2008; Zavjalova 2008; Efimov 2008; Bagrikova 2009; Bednarska 2009; Danylyk and Honcharenko 2009; Fitsajlo and Orlov 2009; Jasinska et al. 2009; Kucherevskyi et al. 2009; Orlov and Gubar 2009; Prots 2009; Peterson et al. 2009; Andryk et al. 2010; Borsukevych 2010; Didukh et al. 2010; Ostapko et al. 2010; Peregrym and Kuzemko 2010; Punina et al. 2010; Zhmud and Zhmud 2010; Bezsmertna 2011; Knyasev 2011; Lazkov 2011; Ryff 2011, 2013; Yena and Shevera 2011; Bezsmertna et al. 2012; Fateryga and Kreutz 2012, 2014; Geltman and Shatko 2012; Hahn 2012; Kreutz and Fateryga 2012; Tzvelev and Geltman 2012; Fateryga et al. 2013, 2014, 2015, 2022; Melnikov 2013; Melnyk et al. 2013; Olshanskyi and Orlov 2013; Orlov and Iakushenko 2013; Yu. Peregrym et al. 2013; Ryff et al. 2013, 2023; Tyshchenko et al. 2013; Bagrikova and Ryff 2014; Burda 2014; Harpke et al. 2014; Nachychko 2014, 2016; Orlov et al. 2014, 2019, 2022; Nobis et al. 2014, 2020; Štěpánek and Kirschner 2014, 2018, 2022a, b; Zhou et al. 2014; Ljubka et al. 2014; Popova 2015; Seregin et al. 2015a, b; Zvyagintseva 2015; Raab-Straube and Raus 2015, 2017, 2019b, a, 2020, 2021b, a, 2023, p. 1, 2024; Nachychko and Honcharenko 2016; Vasjukov 2016; Parnikoza and Celka 2016; Sołtys-Lelek and Oliіar 2016; Kipriyanova and Shadrin 2017; Gouz and Timoshenkova 2017; Bednarska and Brazauskas 2017; Mosyakin 2017; Bondareva et al. 2018; Ostapko 2018, 2020; Shiyan 2018; Mayorov 2018; Novák and Zukal 2018; Bulakh et al. 2019, 2020; Shynder 2019; Wolf et al. 2019; Kuzemko et al. 2019; Novikov et al. 2020; Orlov and Shevera 2020, 2021; Shynder et al. 2020, 2022a, b, c, 2024; Takács et al. 2020; Shevera et al. 2020; Mosyakin and Mandák 2020; Kechaykin et al. 2020; Bagrikova et al. 2021; Moysiyenko et al. 2021, 2023; Mosyakin and Mosyakin 2021; Dudáš et al. 2022; German 2022; Kobiv et al. 2022; Shalimov 2022; Bagrikova and Perminova 2022; Peregrym and Koopman 2023; Štěpánek et al. 2023; Mátis et al. 2023; Krahulec et al. 2023; Sennikov and Tikhomirov 2024a, b; Shiyan et al. 2024; Bronskov and Bronskova 2024).

### Approaches used for data analysis

Generalized information on all floristic discoveries, including supporting data (location, habitat type, date of collection, species origin, and notes), is presented in a tabular format in the Supplementary Material. Taxa of the highest ranks, including newly described species and subspecies of vascular plants reported for the first time in the flora of Ukraine, are listed according to poster overviews based on recent molecular phylogenetic syntheses (Cole et al. 2021; Cole 2022). The order of families and species follows the Latin alphabet, and taxonomic volumes are accepted according to Plants of the World Online (https://powo.science.kew.org)*.*

For the analysis of habitats associated with the newly described or newly recorded taxa in Ukraine, we used the classification system proposed in the National Habitat Catalogue of Ukraine (Kuzemko et al. 2018). All types of habitats are divided into nine groups, which are mainly in line with the major groups of the European Nature Information System – EUNIS (https://eunis.eea.europa.eu): marine, coastal, aquatic, wetlands, grasslands, scrub, forests, stone outcrops and other sparsely vegetated habitats, synanthropic. Some species exhibited broad ecological amplitudes and were therefore recorded in multiple habitat types. For 18 taxa, the habitat information was not specified in the original publications and remains unknown.

To identify the most productive periods of floristic discovery within the year, whether describing new taxa or recording species or subspecies new to the territory of Ukraine, we divided the year into four quarters: 1^st^ quarter – January, February, March; 2^nd^ quarter – April, May, June; 3^rd^ quarter – July, August, September; and 4^th^ quarter – October, November and December. Next, all available dates of herbarium specimens or field observations cited in the analyzed publications (a total of 490 records; however, 30 of these specified only the year without an exact date) were compiled. This allowed us to identify the periods when the highest numbers of discoveries were made. Monthly-level analysis was avoided due to calendar discrepancies: a part of the country transitioned from the Julian to the Gregorian calendar on February 14, 1918 (with a 13-day difference between the two), and it is not always clear which calendar style was used in the original sources.

### Licences

Figure 2 was prepared as a modified version of the map “Ukraine, administrative divisions” by TUBS (2025), available under the Creative Commons Attribution–ShareAlike 3.0 license (CC BY-SA 3.0; https://creativecommons.org/licenses/by-sa/3.0*)*, via Wikimedia Commons.

## Results

### General information

The total number of new species and subspecies of vascular plants reported for the first time within Ukraine during the studied period is 331, however presently accepted ones are only 314. There are 1 species of lycopods, 9 species and subspecies of ferns, 2 species of gymnosperms, and 302 species and subspecies of flowering plants (1 species of magnoliids, 78 taxa of monocots and 223 taxa of eudicots). New ferns are represented by 5 genera and 5 familes, gymnosperms – by 2 genera and families, and angiosperms – by 164 genera and 59 families (1 genus and family of magnoliids, 46 genera and 14 families of monocots, as well as 117 genera and 44 families of eudicots). *Aizoaceae* Martinov, *Menispermaceae* Juss., *Talinaceae* (Fenzl) Doweld are new families for the spontaneous flora of Ukraine, as well as 31 genera (*Althenia* F. Petit, *Ampelopsis* Michx., *Apocynum* L., *Axyris* L., *Calligonum* L., *Cyclospermum* Lag., × *Dactylocamptis* P.F. Hunt & Summerh, *Diplachne* P. Beauv., *Dysphania* R. Br., *Eclipta* L., *Gelasia* Cass., *Groenlandia* J. Gay, *Klasea* Cass., *Koelreuteria* Laxm., *Lomelosia* Raf., *Macleaya* R. Br., *Malcolmia* W.T. Aiton, *Menispermum* Tourn. ex L., *Mesembryanthemum* L., *Nerium* L., *Olimarabidopsis* Al-Shehbaz, O’Kane & R.A. Price, *Parentucellia* Viv., *Petrosedum* Grulich, *Phedimus* Raf., *Puschkinia* Adams, *Sporobolus* R. Br., *Symphyotrichum* Nees, *Talinum* Adans., *Trichophorum* Pers., *Triodanis* Raf., *Tyrimnus* (Cass.) Bosc) are reported here for the first time.

The largest numbers of new taxa were found within next families: *Asteraceae* Bercht. & J. Presl (52 species and subspecies), *Poaceae* Barnhart and *Rosaceae* Juss. (22 species each), *Orchidaceae* Juss. (20 species and subspecies), *Portulacaceae* Juss. (10 species), *Amaryllidaceae* J. St.-Hil., *Brassicaceae* Burnett and *Lamiaceae* Martinov (9 species each), *Papaveraceae* Juss. (8 species), *Cyperaceae* Juss., *Cactaceae* Juss. and *Caryophyllaceae* Juss. (7 species each). The most discoveries of new taxa within genera were made in following ones: *Taraxacum* F.H. Wigg. (33 species), *Rubus* L. (11 species), *Epipactis* Zinn and *Portulaca* L. (10 taxa each), *Allium* L. (9 taxa), *Opuntia* Mill. (7 species), *Viola* L. (6 taxa), *Euphorbia* L. (5 species), *Thymus* L. (4 species), *Asplenium* L., *Cardamine* L., *Crataegus* L., *Cotoneaster* Medik., *Dryopteris* Adans., *Erigeron* L., *Festuca* Tourn. ex L., *Juglans* L., *Lonicera* L., *Oxalis* L., *Papaver* L., *Plantago* L., *Poa* L., *Rumex* L., and *Veronica* L. (3 taxa within each).

**Fig. 1:**
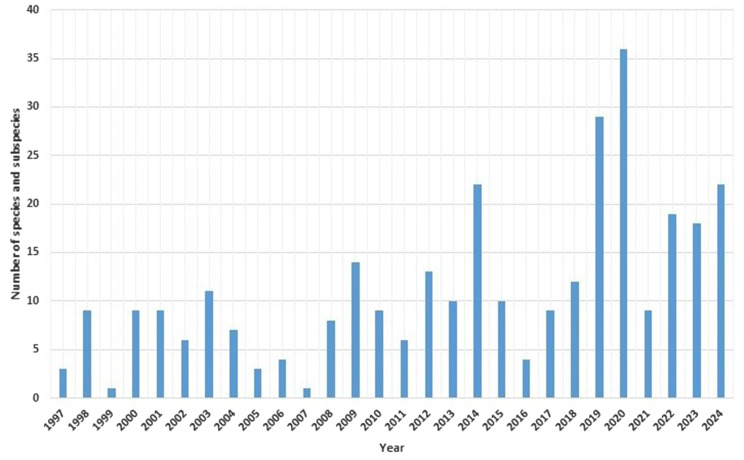
Annual number of vascular plant species and subspecies newly recorded from Ukraine, based on the publication dates of relevant studies (1997–2024)

The average number of accepted vascular plant species and subspecies newly recorded from Ukraine per year, based on the publication dates of the relevant studies, was 11.18 ± 8.26. The annual distribution of published discoveries is presented in Fig. 1.

### New taxa for science

The description of 57 new taxa for science from the territory of Ukraine during the studied period is very notable: 46 species (14 nothospecies among them) and 11 subspecies (3 nothosubspecies). However, not all of them are recognized at present, a part of them (3 species and 4 subspecies) is already considered as synonyms to other taxa. For example, *Brachypodium pinnatum* (L.) P. Beauv. subsp. *juzepczukii* Tzvelev is not accepted by POWO, and it is simply considered as *B. pinnatum* (POWO, 2025); or *Arenaria martrinii* Tzvel. which is considered as a synonym to *A. serpyllifolia* L. subsp. *serpyllifolia* (POWO, 2025). Though there is a group of other examples: in particular, newly described *Gagea microfistulosa* Levichev is already considered as *G. polidorii* J.-M. Tison (Peterson et al. 2009), nevertheless it is a new species for the flora of Ukraine.

Almost half of these new taxa have been described from the Crimean Peninsula (25 species and subspecies), 7 taxa from both Transcarpathia and Kherson region, 6 taxa from Ivano-Frankivsk region, 3 taxa from all Luhansk, Donetsk and Mykolaiv regions, 2 taxa from both Chernivtsi and Odesa regions, as well as Kharkiv, Poltava, Dnipro, Zaporizhzhia, Kyiv, Rivne, Ternopil’, Lviv regions and Kyiv were mentioned among typical materials. Also large areas like Ukraine, the North part of Ukraine, Western Polissya, Prycarpathia and the Carpathians for a whole without any clarification were noted in descriptions of these new taxa. Besides, it is worthing to note that 40 taxa were described based on old herbarium materials, as well as these collections were used when identifying 49 taxa known early for science, but first noted for the flora of Ukraine. The largest number of new taxa for science were described among following genera: *Taraxacum* (7 species), *Thymus* (4 species), *Viola* (3 species and 1 subspecies), *Allium* (3 species), *Poa* (3 species), *Gagea* Salisb. and *Festuca* (2 species each), and *Epipactis* (1 species and 1 subspecies).

### Hybrids

It should be noted that a significant number (39 taxa) of all discovered or described taxa in the flora of Ukraine are hybrids. Most of them are nothospecies, but there are 4 nothosubspecies (*Anacamptis × simorrensis* (E.G. Camus) H. Kretzschmar, Eccarius & H. Dietr. nothosubsp. *ticinensis* (Gsell) Fateryga & Kreutz (= *A. coriophora* (L.) R.M. Bateman, Pridgeon & M.W. Chase subsp. *coriophora* × *A.pyramidalis* (L.) Rich.), *×Dactylocamptis uechtritziana* (Hausskn.) B. Bock ex M. Peregrym & Kuzemko nothosubsp. *magyarii* (Soó) Fateryga & Kreutz (= *Anacamptis palustris* (Jacq.) R.M. Bateman, Pridgeon & M.W. Chase subsp. *elegans* (Heuff.) R.M. Bateman, Pridgeon & M.W. Chase × *Dactylorhiza incarnata* (L.) Soó), *Orchis ×-beyrichii* Kern. nothosubsp. *mackaensis* (Kreutz) Fateryga & Kreutz (*O. militaris* L. subsp. *stevenii* (Rchb. f.) B. Baumann, H. Baumann, R. Lorenz & Ruedi Peter × *O. simia* Lam.), *Viola* ×*popovae* Vl. Nikit. nothosubsp. *romankoshica* Vl. Nikit. (= *V. nemoralis* Kijrtz. subsp. *abbreviata* Vl. Nikit. × *V. sieheana* W. Beck.)), also 3 taxa are intergeneric hybrids (×*Dactylocamptis uechtritziana* (= *Dactylorhiza incarnata* × *Anacamptis palustris*), *×Dactylocamptis uechtritziana* nothosubsp. *magyarii* (= *Anacamptis palustris* subsp. *elegans* × *Dactylorhiza incarnata*), ×*Agrotrigia hajastanica* (Tzvelev) Tzvelev (= *Agropyron cristatum* (L.) Gaertn. s.l. × *Elytrigia repens* (L.) Desv. ex Nevski)) and 2 taxa are hybrids of three species (*Lonicera ×muendeniensis* Rehder (= *L. morrowii* A. Gray × *L. ruprechtiana* Regel × *L. tatarica* L.), *Crataegus monogyna* Jacq. × *C. laevigata* (Pior.) DC. × *C. rhipidophylla* Gand.). The largest number of taxa of hybridogenic origin belong to the following families: *Orchidaceae* (8 taxa), *Lamiaceae*, *Poaceae* (4 taxa each), *Rosaceae* and *Violaceae* Batsch (3 taxa). Among genera: *Thymus* (4 taxa), *Crataegus* and *Viola* (3 taxa each). Only 7 hybrid taxa have alien origin (*Equisetum* ×*moorei* Newman, *Lolium ×elongatum* (Ehrh.) Banfi, Galasso, Foggi, Kopecký & Ardenghi, *Mesembryanthemum ×vascosilvae* (Gideon F. Sm., E. Laguna, F. Verloove & P.P. Ferrer) Sáez & Aymerich, *Symphyotrichum* ×*salignum* (Willd.) G.L. Nesom, *Lonicera ×muendeniensis*,* Oenothera ×wienii* Renner ex Rostański, *Vitis ×instabilis* Ardenghi, Galasso, Banfi & Lastrucci), and 4 of them (*Lolium ×elongatum*, *Mesembryanthemum ×vascosilvae*, *Symphyotrichum* ×*salignum*, *Lonicera ×muendeniensis*) are considered as escaped from their cultivation in Kyiv Botanical Gardens and Syrets Arboretum. The largest number of native taxa of hybrid origin were identified within the Crimean Peninsula (17), as well as 2 taxa each from the Lugansk, Donetsk and Transcarpathian regions. Basically, new taxa of hybridogenic origin were identified from fresh field materials of the authors, but 10 species were identified from herbarium material, and 8 of the latter (*Otites ×klopotovii* Tzvelev, *Hedysarum ×smirnovii* Knjasev, *Thymus alternans* Klokov × *T. pulegioides* L., *Paeonia ×maleevii* Kem.-Nath. ex Mordak & Punina, *Crataegus* ×*poplavskae* Tzvelev, *Viola ×bachtschisaraensis* Vl. Nikit., *V. ×poltavensis* Vl. Nikit, *V.* ×*popovae* Vl. Nikit. nothosubsp. *romankoshica* Vl. Nikit.) were described for the first time for science.

### Regions where new taxa were found or described

**Fig. 2:**
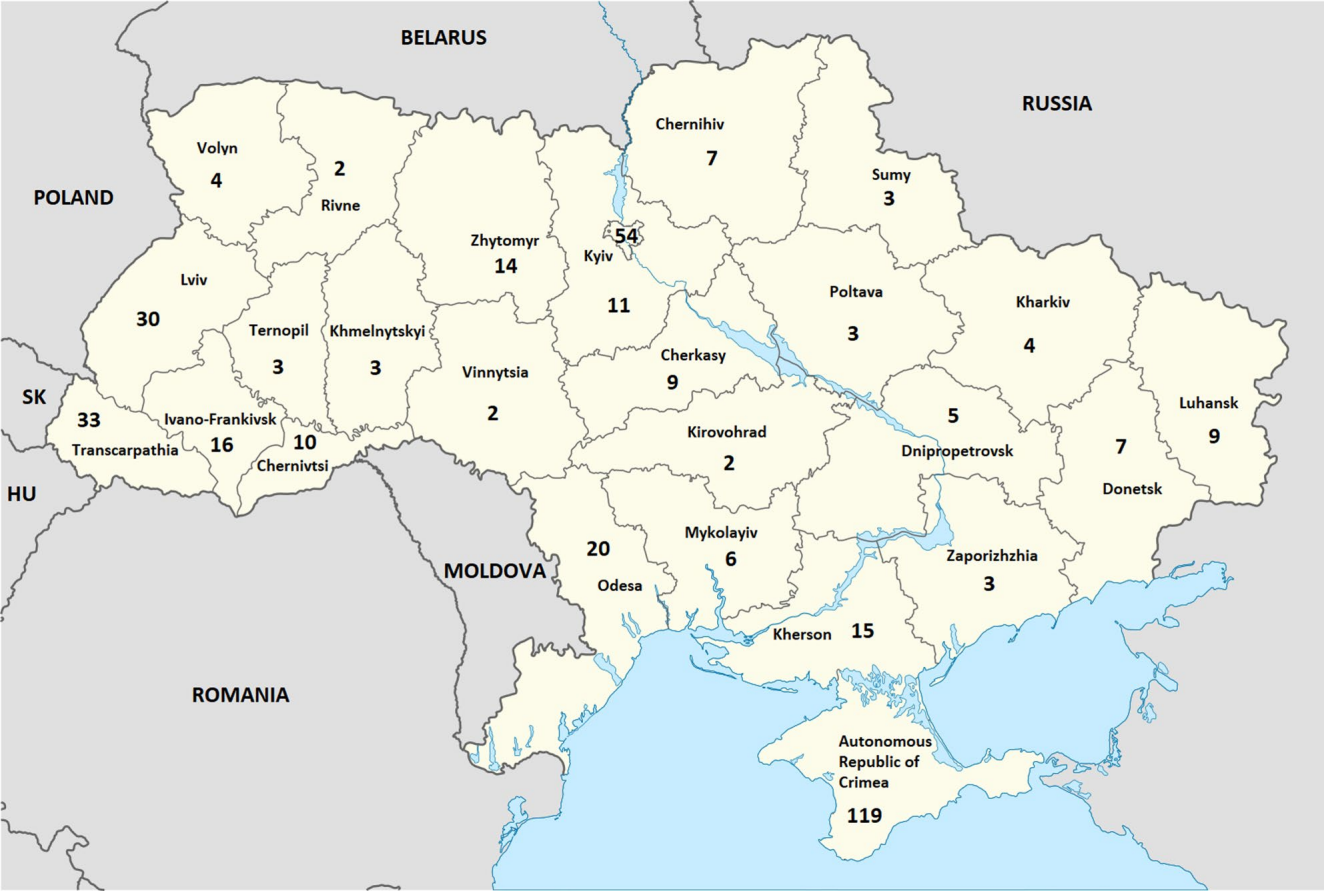
Geographic distribution of new species and subspecies of vascular plants recorded for the flora of Ukraine during 1997–2024, shown by administrative regions

Overall, the Autonomous Republic of Crimea, located on the Crimean Peninsula, is the clear leader in the number of new species and subspecies discovered for the flora of Ukraine, with 119 taxa recorded. The second highest number was documented for Kyiv city (54 taxa), followed by Transcarpathian region (33 taxa), Lviv region (30 taxa), Odesa region (20 taxa), Ivano-Frankivsk region (16 taxa), Kherson region (15 taxa), Zhytomyr region (14 species), Kyiv region (11 taxa), Chernivtsi region (10 taxa), Cherkasy and Luhansk regions (9 taxa each), Chernihiv and Donetsk regions (7 species each), Mykolaiv region (6 species), Dnipropetrovsk region (5 species), Kharkiv and Volyn regions (4 species each), Khmelnytskyi, Poltava, Sumy, Ternopil, and Zaporizhzhia regions (3 taxa each), and Kirovohrad, Rivne, and Vinnytsia regions (2 species each). This territorial distribution is illustrated in Fig. 2.

It should be noted that several large geographic areas, such as the northern, western, and southeastern parts of Ukraine, North Polissya, the Carpathians, and Podillia, were mentioned in publications without precise reference to administrative units. Furthermore, many species and subspecies were recorded for the first time from multiple locations, often situated in different administrative regions; therefore, the total number of localities substantially exceeds the number of taxa.

### The origin of new taxa: native and alien ones

The majority, 166 taxa, among all recently described and discovered taxa has natural origin in Ukraine. Other 139 taxa are alien, and the origin of nine taxa is not precisely determined: seven of them are most likely of natural origin, and two ones are most likely of alien origin. There are two groups of new alien taxa: the bigger one includes plants escaped from cultivation (76 taxa) and the smaller one (63 taxa) plants brought in by transport, cargo transportation or in some other way as a result of anthropogenic activity. If to exclude data about newly described taxa for science which are given above, the largest number of recently noted native plants were found within the Crimean Peninsula (47 taxa) and in some of western regions of Ukraine: Lviv region − 28 taxa, Transcarpathia − 13 taxa and Ivano-Frankivsk region - nine taxa. Also four species were discovered in Luhansk region, three species - in Kherson region, two new taxa were reported from each of the following regions: Chernivtsi, Kharkiv, Odesa, Sumy, Volyn’ and Zhytomyr regions. Also, one new taxon was reported from Cherkasy, Chernihiv, Dnipropetrovsk, Khmylnytskyi, Mykolaiv, Rivne, Ternopil’, Vinnytsia regions and Kyiv.

The largest number of new alien species escaped from the cultivation (49 taxa) are noted in Kyiv city. Also 16 such taxa found in the Autonomous Republic of Crimea, seven taxa - in each of Cherkasy and Odesa regions, six taxa - in Transcarpathia, five taxa - in each of Kyiv and Zhytomyr regions, and three taxa - in Dnipropetrovsk region. As well, two taxa of escaped plants detected in each of Chernighiv, Chernivtsi, Donetsk, Khmelnytskyi, Kirovohrad and Mykolaiv regions, only one taxon - in each of Ternopil’ and Zaporizhzhya regions. The current situation is somewhat different with regard to alien plants which entered the flora of Ukraine in other ways. Namely, the majority of such species were discovered in Crimea (26 taxa), as well as eight taxa in each of Odesa region and Transcarpathia. Six new alien species were noted in Kherson region, five taxa - in each of Kyiv city, Chernivtsi and Zhytomyr regions. Only two such new alien species were found in each of Kharkiv, Kyiv and Luhansk regions, as well as one species - in each of Cherkasy, Chernihiv, Donetsk, Lviv, Sumy, Vinnytsia and Volyn’ regions.

### Habitats

The majority of taxa newly recorded for the flora of Ukraine are associated with synanthropic habitats (123 taxa). In addition, *Chenopodium ucrainicum* Mosyakin & Mandák, a species newly described for science, also occurs in such habitats. As noted in the previous section, a substantial portion of these taxa are escapees from cultivation, primarily distributed in synanthropic environments of neighboring regions. Grasslands represent the second most significant habitat type, with 56 taxa newly recorded for Ukraine and 33 taxa newly described for science. Woodlands rank third, comprising 25 taxa newly recorded for Ukraine and 9 newly described taxa. Stone outcrop habitats host 12 taxa newly recorded for Ukraine and 4 newly described for science. In coastal habitats, 7 new species and 1 subspecies were identified for the flora of Ukraine, while 3 species were newly described from these environments. Although no species or subspecies were newly described for science from aquatic, wetland, or shrub habitats, new records for Ukraine’s flora were documented as follows: 18 taxa from aquatic habitats, 8 from wetlands, and 7 from shrublands. Regrettably, the habitat types of 19 taxa could not be determined, as this information was not provided in the original publications.

### Plant collection and publication dates

The highest number of floristic discoveries were based on herbarium collections and field observations made during the second quarter of the year (April–June), with a total of 213 records. The third quarter (July–September) was nearly as productive, yielding 198 records. Notably, the colder half of the year, from mid-autumn to mid-spring, also contributed to floristic discoveries: 46 records were collected in the fourth quarter (October–December), and 5 in the first quarter (January–March). These data provided the documentary basis for either newly described species and subspecies or new records for the flora of Ukraine.

The majority of herbarium collections and field observations that underpinned floristic discoveries (317 records, or 64.3%) were made during the period covered by the analyzed publications, i.e., from 1997 to 2024. The most productive years within this period were 2017 (37 records) and 2020 (34 records). Notable earlier years outside the publication timeframe include 1989 and 1990, with 8 and 10 records, respectively.

In terms of the timing of publication of floristic discoveries (see Fig. 1), the most productive years were 2020, with 36 taxa reported, and 2019, with 29 taxa.

### Herbarium collection

It is important to emphasize that 82 floristic discoveries, representing 26.1% of all recorded floristic discoveries for the flora of Ukraine during the studied period, were based on herbarium materials. Notably, 34 of these cases involved the description of new species and subspecies for science, accounting for 69.4% of all taxa newly described from Ukraine within the analyzed timeframe. The publications reviewed in this study include data on 19 herbarium specimens collected in the 19^th^ century, 66 collected during the first half of the 20th century, and 78 collected between 1951 and 1990. The oldest specimen is a collection of *Botrychium simplex* E. Hitchc. from the vicinity of Kharkiv, dated 12 June 1828.

### Citizen science platforms

The growing role of citizen science platforms in contributing to floristic discoveries in Ukraine is also worth referencing. Although no new species or subspecies of vascular plants have yet been formally described solely on the basis of data from these platforms, several noteworthy findings have emerged through their use. For example, *Astragalus calycinus* M. Bieb was discovered with the help of the Plantarium platform (https://www.plantarium.ru), while the occurrence of *Tamarix laxa* Willd. was partially confirmed through observations on iNaturalist (https://www.inaturalist.org)*.* Over the past decade, platforms such as iNaturalist have increasingly been considered as complementary, or, in some cases, alternative, sources of data to herbarium collections. Although their precise role in the analyzed floristic discoveries remains unclear, references to observations from these platforms are increasingly cited in relevant publications.

## Discussion

The results of this study demonstrate that, despite Ukraine’s long history of floristic research and relatively accessible territory, significant discoveries of vascular plant taxa continue to be made. This underscores a broader truth: even in relevantly well-studied temperate regions, floristic inventories remain incomplete, and ongoing fieldwork, herbarium study, and taxonomic revision remain essential.

The predominance of synanthropic habitats among discovery sites highlights that human-altered landscapes are a key frontier for floristic exploration. Many new recorded taxa, particularly alien species, were found in urban and peri-urban environments or near transport corridors, reinforcing the view that synanthropic habitats are dynamic interfaces between cultivated and spontaneous floras (Steen et al. 2024). These areas also act as primary zones for naturalization and early establishment of alien species, including potential future invasives. Thus, regular monitoring of such habitats should be an integral component of national biodiversity assessments (Galera and Sudnik-Wójcikowska 2011; Podda et al. 2025). Notably, Ukrainian botanists have only recently begun to systematically investigate escaped cultivated taxa, with several species now documented outside botanical gardens and arboreta for the first time (Konaikova et al. 2015; Peregrym et al. 2016; Shynder 2019; Shynder et al. 2020, 2022a, b; Konaikova and Peregrym 2023).

Separately, it is important to emphasize that, based on historical experience and previous research (Santini et al. 2023), the introduction and documentation of new alien plant taxa in Ukraine are expected not only to continue but likely to intensify in the coming years. The ongoing full-scale Russian invasion of Ukraine represents a major driver of this process, as large-scale movements of military equipment, ammunition, supplies, food, and personnel from multiple regions of the world increase the risk of unintentional species introductions. At the same time, widespread habitat destruction caused by military activities is generating extensive disturbed environments and novel ecological niches that may facilitate the establishment and spread of alien plant species.

Grasslands and forests featured prominently in floristic discoveries for taxa of native origin in Ukraine. These results echo findings from other European countries, where steppe remnants and montane areas serve as refugia for poorly studied or cryptic taxa (Birks and Willis 2008; Kajtoch et al. 2016; Kirschner et al. 2020). The Crimean Peninsula, in particular, emerged as a hotspot of both endemism and ongoing discovery, contributing nearly one-third of all new taxa, including many of hybrid origin. This underlines the biogeographic uniqueness of Crimea and the urgent need for its continued botanical exploration and protection of its natural areas that, unfortunately, it looks almost impossible in the conditions of the Russian-Ukrainian war at the present.

Herbarium collections are playig a substantial role in documenting floristic novelties, with over a quarter of the discoveries based on historical specimens. In many cases, herbarium samples collected in the 19th and early 20th centuries provided the basis for the formal description of new taxa. This highlights the critical importance of herbarium digitization, curation, and re-examination using contemporary taxonomic frameworks, especially in regions where field access may be limited due to geopolitical or logistic constraints.

The growing influence of citizen science platforms, particularly iNaturalist (Mesaglio and Callaghan 2021), reflects a shift in how floristic data are collected and verified. Moreover, such platforms have already directly supported species descriptions, although there have been no such cases in Ukraine yet, as well as their role in early detection and range confirmation of taxa is increasing (Mesaglio et al. 2025). The integration of citizen science observations with formal taxonomic research holds promise, especially in regions where professional botanists are few. However, the challenges of data quality, taxonomic expertise, and long-term archiving must be carefully addressed (Aceves-Bueno et al. 2017; López-Guillén et al. 2024).

Temporal patterns in floristic discoveries expectedly suggest that April–September are the most productive time, corresponding to the active growing season of most vascular plants. Nonetheless, records from late autumn and early spring demonstrate that year-round vigilance can be rewarding.

Overall, the Ukrainian case illustrates that floristic discovery remains a vital component of biodiversity research even in intensively studied floras. Focusing survey efforts on underexplored habitats (especially synanthropic and steppe), biogeographic hotspots (Crimea, Carpathians), and taxonomically complex groups (e.g., *Taraxacum*, *Rubus*, *Viola*), while fostering collaboration among professionals, citizen scientists, and herbarium curators, can accelerate the rate of discovery. These strategies are widely transferable and can inform biodiversity assessment, conservation planning, and invasive species management across temperate regions worldwide.

## Conclusions

This study demonstrates that, even in a relevantly well-studied temperate flora, new national records and taxa new to science continue to emerge at a substantial rate. Analysis of nearly three decades of discoveries in Ukraine revealed consistent drivers: underexplored synanthropic and grassland habitats, mountainous regions rich in endemism and hybridization, and taxonomically complex plant groups. Historical herbarium collections and, increasingly, citizen science platforms also played important roles in expanding floristic knowledge.

These findings highlight the importance of integrating targeted field surveys, systematic herbarium re-examination, and collaborative networks involving both professionals and trained citizen scientists. The methodological and ecological patterns identified here are relevant well beyond Ukraine and can help improve biodiversity inventories, guide conservation priorities, and strengthen early detection of invasive species in other temperate regions.

Also, this research is an important contribution for the future updating of the current checklist of the flora of Ukraine (Mosyakin and Fedoronchuk 1999).

## Supplementary Information

Below is the link to the electronic supplementary material.

Supplementary Material 1

## Data Availability

All data generated or analysed during this study are included in this published article and its supplementary information file.

## References

[CR1] Aceves-Bueno E, Adeleye AS, Feraud M et al (2017) The accuracy of citizen science data: a quantitative review. Bull Ecologic Soc Am 98:278–290. 10.1002/bes2.1336

[CR2] Agapova N (1998) Generis *Ornithogalum* L. (Hyacinthaceae) species nova. Novitates Systematicae Plant Vascularium 31:29–35

[CR3] Albach DC (2008) Further arguments for the rejection of paraphyletic taxa: *Veronica* subgen. *Pseudolysimachium* (Plantaginaceae) Taxon 57:1–6

[CR4] Andryk YY, Balog L, Shevera MV (2010) *Humulus japonica* Siebold. et Zucc. (Cannabaceae) is a new adventive species of flora of Ukraine. Ukr Bot J 67:438–445

[CR5] Bagrikova NA (2009) *Fumaria capreolata* L. – a new adventive species in the flora of Ukraine. Ukrainian Bot J, 66:49–52

[CR6] Bagrikova NA, Perminova YA (2022) Characteristics and distribution of the *Opuntia* (Cactaceae) representatives naturalized in Crimea. Trudy po Prikladnoy Botanike, Genetike i Selektsii 183:149–160. 10.30901/2227-8834-2022-3-149-160

[CR7] Bagrikova NA, Ryff LE (2014) On naturalization of representatives of the genus *Opuntia* Mill. on the territory of the Crimean Peninsula. In: Abstracts of the International Scientific Conference. Kherson, pp 19–21

[CR8] Bagrikova NA, Ryff LE, Chichkanova YS, Perminova YA (2021) Characteristics of fruit and seeds of representatives of the genus *Opuntia* (Cactaceae) naturalized in Crimea. Bot J 106:1002–1015. 10.31857/S0006813621100033

[CR9] Baker WJ (2014) Discovering plant diversity – are we up to speed? In: Royal Botanical Gardens Kew. https://www.kew.org/read-and-watch/discovering-plant-diversity. Accessed 1 Feb 2024

[CR10] Bednarska I (2009) *Festuca polovina* Bednarska is a new for science notospecies in the flora of Ukraine. Ukrainian Bot J 66:29–34

[CR11] Bednarska I, Brazauskas G (2017) *Festuca galiciensis*, a new species of the *F. valesiaca* group (Poaceae) from Ukraine. Phytotaxa 306:21. 10.11646/phytotaxa.306.1.2

[CR12] Bello A, Edie SM, Yessoufou K, Muellner-Riehl AN (2023) Trends in botanical exploration in Nigeria forecast over 1000 yet undescribed vascular plant species. Ann Botany 133:789–800. 10.1093/aob/mcad106 10.1093/aob/mcad106 PMC11082469 37503672

[CR13] Bezsmertna OO (2011) *Dryopteris villarii* (Вellardi) Woynar ex Schinz et Thell. (Dryopteridaceae), a new species for the flora of Ukraine. Ukrainian Bot J 68:829–832

[CR14] Bezsmertna OO, Peregrym MM, Vasheka OV (2012) Genus *Asplenium* L. (Aspleniaceae) in the natural flora of Ukraine. Ukrainian Bot J 69:544–558

[CR15] Birks HJB, Willis KJ (2008) Alpines, trees, and refugia in Europe. Plant Ecol Divers 1:147–160. 10.1080/17550870802349146

[CR16] Bondareva LV, Ryff LE, Svirin SA, Yevseenkov PE (2018) Botanical and geographical phenomenon of the Sevastopol region in connection with new floristic finds. Materials II Intern. conf. (to the 90^th^ anniversary of the birth of Prof. A.G. Yelenevsky). MPGU, Moskow, pp 108–111

[CR17] Borsukevych LM (2010) *Groenlandia densa* (L.) Fourr. (Potamogetonaceae) – a representative of a genus new to the flora of Ukraine. Ukrainian Bot J 67:100–103

[CR18] Bronskov OI, Bronskova OM (2024) *Tamarix laxa* (Tamaricaceae), a new species in the flora of Ukraine. Ukr Bot J 81:229–241. 10.15407/ukrbotj81.03.229

[CR19] Bulakh OV, Protopopova VV, Shevera MV (2019) *Portulaca cypria* Danin, *P.**granulatostellulata* (Poelln.) Ricceri & Arrigoni, *P.**papillatostellulata* (Danin & H.G. Baker) Danin (Portulacaceae Juss.) – taxa new to the flora of Ukraine from Transcarpathia. Bulletin of the Chernivtsi National University 10:87–92

[CR20] Bulakh OV, Volutsa O, Tokaryuk A et al (2020) *Portulaca oleracea* aggregate (Portulacaceae) from the Chernivtsi region (Ukraine). Biolohichni Systemy 12:251–262. 10.31861/biosystems2020.02.251

[CR21] Burda RI (2014) Spontaneous distribution of *Aristolochia macrophylla* (Aristolochiaceae) in Koncha–Zaspy forests (Kyiv). Ukrainian Bot J 71:558–562

[CR22] Byalt VV, Orlova LV (2003) *Egeria densa* Planch. (Hydrocharitaceae) – a new adventive species for the flora of Ukraine. Novitates Systematicae Plant Vascularium 35:211–214

[CR23] Cheek M (2024) Kew’s top 10 new species of 2023. In: Royal Botanical Gardens Kew. https://www.kew.org/read-and-watch/top-10-species-2023. Accessed 1 Feb 2024

[CR24] Cherepanov SK (1995) Vascular plants of Russia and adjacent states (the former USSR). Cambridge University Press, Cambridge [England]; New York

[CR25] Cherepanov SK (2007) Vascular plants of Russia and adjacent states (The former USSR), digitally printed version. Cambridge University Press, Cambridge

[CR26] Chorna GA, Protopopova VV, Shevera MV, Fedoronchuk MM (2006) *Elodea nuttallii* (Planch.) St. John (Hydrocharitaceae) – a new species for the flora of Ukraine. Ukrainian Bot J, 63:328–332

[CR27] Christenhusz MJM, Byng JW (2016) The number of known plants species in the world and its annual increase. Phytotaxa 261:201. 10.11646/phytotaxa.261.3.1

[CR28] Cole TCH (2022) Plant Phylogeny Posters (PPP) – poster titles and languages with links. https://www.researchgate.net/publication/344193089_Plant_Phylogeny_Posters_PPP_-_poster_titles_and_languages_with_links. Accessed 17 Feb 2022

[CR29] Cole TCH, Hilger HH, Bachelier JB et al (2021) Spanning the Globe – The plant phylogeny poster (PPP) project. Ukrainian Bot J 78:235–241. 10.15407/ukrbotj78.03.235

[CR30] Danylyk IM, Honcharenko VI (2009) *Schoenoplectus pungens* (Vahl) Palla (Cyperaceae) – a new species of the Ukrainian flora. Ukrainian Bot J 66:650–655

[CR32] Danylyk IM, Panchenko SM (2001) *Carex brunnescens* (Pers.) Poiret (Cyperaceae) – a new species for the flora of Ukraine. Ukrainian Bot J, 58:73–77

[CR31] Danylyk IM, Myhaly AV, Kish RY (2007) *Trichophorum* Pers. (Cyperaceae) – new genus for the Ukrainian flora. Ukrainian Bot J 64:905–909

[CR33] Davis J (2023) Natural History Museum scientists described a record 815 new species in 2023. In: Natural History Museum. https://www.nhm.ac.uk/discover/news/2023/december/natural-history-museum-scientists-described-815-new-species-2023.html. Accessed 1 Feb 2024

[CR35] Didukh YP (2010) The «Red Data Book of Ukraine. Plant Kingdom». An afterword. Ukrainian Bot J 67:481–503

[CR37] Didukh YP, Boratynski A (2002) The genus *Celtis* L. (Ulmaceae) in the flora of Ukraine. Ukrainian Bot J 59:5–9

[CR34] Didukh M, Kuzemko A, Mazur T, Vinichenko T (2010) *Nuphar pumila* (Timm.) DC. (Nymphaeaceae Salisb.) – a new species for the flora of Ukraine. Bull Taras Shevchenko Natl Univ Kyiv Series: Introduction Conserv Plant Divers 28:10–16

[CR36] Didukh YaP (ed) (2009) The Red Data Book of Ukraine. Plant Kingdom. Globalconsulting, Kyiv

[CR38] Dubyna DV, Zhmud OI, Chorna GA (2003) New species of *Eclipta prostrata* (L.) L. (Asteraceae) and *Diplachne fasticularis* (Lam.) P. Beauv. (Poaceae). Ukrainian Bot J 60:419–426

[CR39] Dudáš M, Király G, Kobiv Y, Pliszko A (2022) New floristic records from Central Europe 9 (reports 122–133). Thaizia J Bot 32:81–90

[CR40] Efimov P (2008) Notes on *Epipactis condensata*, *E. rechingeri* and *E. purpurata* (Orchidaceae) in the Caucasus and Crimea. Willdenowia 38:71. 10.3372/wi.38.38104

[CR41] Egorova T (1998) Genus *Papaver* L. (Papaveraceae) in flora Europae Orientalis. Novitates Systematicae Plant Vascularium 31:92–120

[CR42] Fateryga AV, Kreutz CAJ (2012) A new *Epipactis* species from the Crimea, South Ukraine (Orchidaceae). J Europäischer Orchideen 44:199–206

[CR43] Fateryga AV, Kreutz CAJ (2014) Checklist of the orchids of the Crimea (Orchidaceae). J Europäischer Orchideen 46:407–436

[CR47] Fateryga VV, Kreutz CAJ, Fateryga AV, Reinhardt J (2013) *Epipactis muelleri* Godfery (Orchidaceae), a new species for the flora of Ukraine. Ukrainian Bot J 70:652–654

[CR44] Fateryga AV, Kreutz KCAJ, Fateryga VV, Efimov PG (2014) *Epipactis krymmontana* (Orchidaceae), a new species endemic to the Crimean mountains and notes on the related taxa in the Crimea and bordering Russian Caucasus. Phytotaxa 172:22. 10.11646/phytotaxa.172.1.3

[CR45] Fateryga VV, Fateryga AV, Svirin SA (2015) *Epipactis leptochila* (Godfery) Godfery (Orchidaceae), a new species for the flora of Russia. Turczaninowia 18:36–40. 10.14258/turczaninowia.18.4.4

[CR46] Fateryga VV, Ivanov SP, Popovich AV, Fateryga AV (2022) On the natural interspecific hybrids of *Ophrys mammosa* Desf. s.l. and *O. oestrifera* M. Bieb. (Orchidaceae) from the Crimea and the North Caucasus. Turczaninowia 25:45–51. 10.14258/turczaninowia.25.1.5

[CR48] Fitsajlo TV, Orlov ОО (2009) *Crataegus ×** dunensis* Cin. (Rosaceae), a new species for the flora of Ukraine. Ukrainian Bot J 66:354–358

[CR49] Galera H, Sudnik-Wójcikowska B (2011) The structure and differentiation of the synanthropic flora of the botanical gardens in Poland. Acta Soc Bot Pol 73:121–128. 10.5586/asbp.2004.017

[CR50] Geltman DV, Shatko VG (2012) The discovery of *Euphorbia hirsuta* L. (Euphorbiaceae) in the Crimea. Ukrainian Bot J 69:604–606

[CR51] German DA (2022) New records and deletions of Cruciferae for Russia and some neighbouring countries. Turczaninowia 25:146–152. 10.14258/turczaninowia.25.1.14

[CR52] Gouz GV, Timoshenkova VV (2017) The first record of *Sporobolus cryptandrus* (Poaceae) for Ukraine and new records for southeastern Ukraine from Triokhizbensky steppe. Ukrainian Bot J 74:64–70. 10.15407/ukrbotj74.01.064

[CR53] Gureyeva II, Page CN (2008) The genus *Pteridium* (Hypolepidaceae) in the Northen Eurasia. Bot J 93:915–934

[CR54] Hahn W (2012) Auf Den Spuren von Christian von Steven: Orchideen- und Bestäuberuntersuchungen im Krimgebirge 2011 und 2012. Ber Arbeitskrs Heim Orchid 29:5–63

[CR55] Harpke D, Peruzzi L, Kerndorff H et al (2014) Phylogeny, geographic distribution, and new taxonomic circumscription of the *Crocus reticulatus* species group (Iridaceae). Turk J Bot 38:1182–1198. 10.3906/bot-1405-60

[CR56] Hey J (2009) Why should we care about species? Nat Educ 2:2

[CR57] Honcharenko VI (2000) New for the flora of Ukraine species of section *corylifolii* LindI. of genus *rubus* L. (Rosaceae). Novitates Systematicae Plant Vascularium 32:53–54

[CR58] iNaturalist https://www.inaturalist.org. Accessed 23 November 2025

[CR59] Jasinska AK, Iakushenko DM, Sobierajska K et al (2009) *Pinus uliginosa* G.E. Neumann ex Wimm. – a new taxon for the Ukrainian flora. Ukrainian Bot J 66:640–646

[CR60] Kajtoch Ł, Cieślak E, Varga Z et al (2016) Phylogeographic patterns of steppe species in Eastern Central Europe: a review and the implications for conservation. Biodivers Conserv 25:2309–2339. 10.1007/s10531-016-1065-2

[CR61] Kechaykin AA, Skaptsov MV, Batkin AA et al (2020) New species of the genus *Asplenium* L. (Aspleniaceae) for the flora of Europe and Russia. Turczaninowia 23:5–9. 10.14258/turczaninowia.23.4.1

[CR62] Kipriyanova LM, Shadrin NV (2017) Two species of aquatic plants new to the Crimean Peninsula. Bot J 102:1683–1689

[CR63] Kirschner J, Štěpánek J (1998) A revision of *Taraxacum* sect. *Piesis* (Compositae) Folia Geobot 33:391–414. 10.1007/BF02803642

[CR64] Kirschner P, Záveská E, Gamisch A et al (2020) Long-term isolation of European steppe outposts boosts the biome’s conservation value. Nat Commun 11:1968. 10.1038/s41467-020-15620-2 32327640 10.1038/s41467-020-15620-2 PMC7181837

[CR65] Knyasev MS (2011) The new hybrid species of the genus *Hedysarum* (Fabaceae) from the East Europe. Bot J 96:1122–1126

[CR66] Kobiv Y, Koutecký P, Štech M, Pachschwöll C (2022) First records of *Calamagrostis purpurea* (Poaceae) in the Carpathians, a relict species new to the flora of Slovakia, Ukraine, and Romania. Biologia. 10.1007/s11756-022-01083-x

[CR67] Konaikova V, Peregrym M (2023) The escape of alien species from botanical gardens: a new example from Ukraine. Biologia 78:1415–1423. 10.1007/s11756-023-01384-9

[CR68] Konaikova VО, Peregrym MM, Gubar LМ (2015) Addition to a list of spontaneous flora of O. V. Fomin Botanic Garden of Taras Schevchenko National University of Kyiv. Studia Biologica 9:159–168. 10.30970/sbi.0902.407

[CR69] Korotchenko I, Peregrym M (2012) Ukrainian steppes in the past, at present and in the future. In: Eurasian Steppes. Ecological problems and livelihoods in a changing world. pp 173–196

[CR70] Krahulec F, Kirschner J, Krahulcová A (2023) *Valeriana dacica*, a distinctive tetraploid in the Eastern Carpathians. Phytotaxa 629:210–222. 10.11646/phytotaxa.629.3.3

[CR71] Krassovskaja LS (2008) On two species of genus *Rubus* L. (Rosaceae) new to the flora of Ukraine. Novitates Systematicae Plant Vascularium 40:84–88

[CR72] Krassovskaja LS, Kagalo AA, Gubareva IY (2001) *Rubus bertramii* (Rosaceae) – a new species for Ukrainian flora. Bot J 86:126–128

[CR73] Krassovskaya LS (1998) On the two species of the genus *Rubus* (Rosaceae) section *Corylifolii* new for the flora of Eastern Europe. Bot J 83:66–68

[CR74] Kreutz CAJ, Fateryga AV (2012) Two taxa of the genus *Epipactis* Zinn (Orchidaceae) new for the flora of Ukraine. Ukrainian Bot J 69:713–716

[CR75] Kucherevskyi VV (2005) A new for science species of *Astragalus* from the Right Bank Black Sea area. Ukrainian Bot J 62:399–403

[CR76] Kucherevskyi VV, Tashev OM, Sirenko TV, Shol GN (2009) New for Ukraine species *Klasea bulgarica* (Acht. et Stoj.) Holub. and its distribution in Europe. Ukrainian Bot J 66:825–832

[CR77] Kuzemko AA, Didukh Ya, Onyshchenko V, Sheffer Y (eds) (2018) National habitat catalogue of Ukraine. FOP Klymenko Yu.Ya., Kyiv

[CR78] Kuzemko AA, Yavorska OG, Kovtoniuk AI (2019) *Cephalaria gigantea* (Caprifoliaceae), a new alien species in the flora of Ukraine. Ukrainian Bot J 76:548–553. 10.15407/ukrbotj76.06.548

[CR79] Kuziarin OT (2003) *Sesleria caerulea* (L.) Ard. (Poaceae) – the new species of the Ukrainian flora from Voroniany (The North-Western Podillia). Ukrainian Bot J 60:182–188

[CR80] Kuziarin OT (2009) Rare anthropophytes for the territory of Lviv region. In: Proceedings of the scientific conference. Shatsk, pp 57–59

[CR81] Kuziarin OT (2012) *Trichophorum alpinum* (L.) Pers. (Cyperaceae), a new species in the flora of Ukraine. Ukrainian Bot J 69:708–712

[CR82] Lazkov G (2011) *Marrubium anisodon* C.Koch (Labiatae), a new species for the Eastern Europe flora. Novitates Systematicae Plant Vascularium 43:84–86

[CR83] Levichev IG (1998) New species of the genus *Gagea* (Liliaceae) from typical section. Bot J 83:110–112

[CR84] Levichev IG (2008) A new species of the genus *Gagea* Salisb. (Liliaceae) from the Crimean Yaila. Novitates Systematicae Plant Vascularium 40:39–46

[CR85] Lindqwister L (2023) What makes a new species a new species? In: California Academy of Sciences. https://www.calacademy.org/phenomena/what-makes-a-new-species-a-new-species. Accessed 1 Feb 2024

[CR86] Ljubka T, Lovas-Kiss Á, Takács A, Molnár VA (2014) *Epipactis albensis* (Orchidaceae) in Ukraine – new data on occurrence and ecology. Acta Bot Hungarica 56:399–408. 10.1556/ABot.56.2014.3-4.14

[CR87] López-Guillén E, Herrera I, Bensid B et al (2024) Strengths and challenges of using iNaturalist in plant research with focus on data quality. Diversity 16:42. 10.3390/d16010042

[CR88] Martin C (2023) New species described by Missouri Botanical Garden scientists in 2023. In: Missouri Botanical Garden. https://discoverandshare.org/2023/12/18/new-species-described-by-missouri-botanical-garden-scientists-in-2023/. Accessed 1 Feb 2024

[CR89] Martin T, Haelewaters D (2023) The art of taxonomy: how is a new species described? In: Operation Wallacea. https://www.opwall.com/article/how-is-a-new-species-described/. Accessed 1 Feb 2024

[CR90] Mátis A, Malkócs T, Kuhn T et al (2023) Hiding in plain sight: integrative analyses uncover a cryptic *Salvia* species in Europe. Taxon 72:78–97. 10.1002/tax.12818

[CR91] Mayorov S (2018) *Datura wrightii* Regel (Solanaceae) – a new alien species for the flora of Russia. Proc KarRC RAS 154–155. 10.17076/bg730

[CR92] Melnikov D (2013) New taxa of the genus *Clinopodium* L. (Lamiaceae). Novitates Systematicae Plant Vascularium 44:174–205

[CR93] Melnyk VI (2012) Dionizy Miklair. To 250 anniversary of birthday. Plant Introduction 55:103–110. 10.5281/ZENODO.2541666

[CR94] Melnyk VI, Goncharenko VI, Savchuk RI (2013) New alien species in the flora of Ukraine, *Tulipa sylvestris* L. (Liliaceae) and *Rubus laciniatus* Willd. (Rosaceae). Ukrainian Bot J 70:232–235

[CR95] Mesaglio T, Callaghan CT (2021) An overview of the history, current contributions and future outlook of iNaturalist in Australia. Wildl Res 48:289–303. 10.1071/WR20154

[CR96] Mesaglio T, Sauquet H, Cornwell WK (2025) Citizen science records are fuelling exciting discoveries of new plant species. Am J Bot e70048. 10.1002/ajb2.70048 10.1002/ajb2.70048 PMC12186130 40375312

[CR97] Mosyakin SL (2017) The first record of *Salsola paulsenii* (Chenopodiaceae) in Ukraine, with taxonomic and nomenclatural comments on related taxa. Ukrainian Bot J 74:409–420. 10.15407/ukrbotj74.05.409

[CR98] Mosyakin SL, Fedoronchuk MM (1999) Vascular plants of Ukraine: A nomenclatural checklist. M. G. Kholodny Institute of Botany, National Academy of Sciences of Ukraine, Kiev

[CR99] Mosyakin SL, Mandák B (2020) *Chenopodium ucrainicum* (Chenopodiaceae / Amaranthaceae sensu APG), a new diploid species: a morphological description and pictorial guide. Ukrainian Bot J 77:237–248. 10.15407/ukrbotj77.04.237

[CR100] Mosyakin SL, Mosyakin AS (2021) Lockdown botany 2020: some noteworthy records of alien plants in Kyiv City and Kyiv Region. Ukrainian Bot J 78:96–111. 10.15407/ukrbotj78.02.096

[CR101] Mosyakin SL, Moysienko II (1999) *Cardaria chalepensis* (L.) Hand.-Mazz. – a new alien species in Ukraine. Ukrainian Bot J, 56:163–166

[CR102] Moysienko II (1998) *Potentilla virgata* Lehm. – a new alien species for the flora of Ukraine. Ukrainian Bot J 55:255–257

[CR103] Moysienko II (2005) *Polygonum alpestre* C.A. May. (Polygonaceae) is a new adventive species for the flora of Eastern Europe. Ukrainian Bot J 62:218–222

[CR104] Moysienko II, Mosyakin SL (2008) *Amaranthus viridis* L. (Amaranthaceae) is a new alien species of flora of Ukraine. Chornomorski Bot J 4:123–127

[CR105] Moysienko II, Yena AV (2006) *Veronica arguteserrata* Reg. et Schmalh. – a new alien species for Ukrainian flora. Chornomorski Bot J 2:104–107

[CR107] Moysiyenko II, Umanets OYu, Dengler J et al (2021) *Torilis pseudonodosa* Bianca (Apiaceae) – new species for the flora of Ukraine. Chornomorski Bot J 17:331–338. 10.32999/ksu1990-553X/2021-17-4-3

[CR106] Moysiyenko II, Shynder OI, Levon AF et al (2023) Notes to vascular plants in Ukraine I. Chornomorski Bot J 19:76–93. 10.32999/ksu1990-553X/2023-19-1-3

[CR108] Nachychko VO (2014) The genus *Thymus* L. (Labiatae Juss.) in the Ukrainian Carpathians’ flora: systematics and taxonomic problems. Visnyk Lviv Univ Biol Ser 64:159–169

[CR109] Nachychko VO (2016) Validation of the name *Thymus* × *pseudoalpestris*. Ann Botanici Fennici 53:401–402. 10.5735/085.053.0611

[CR110] Nachychko VO, Honcharenko VI (2016) Hybrids of *Thymus* L. (Lamiaceae) genus in flora of the Western regions of Ukraine: taxonomic composition and distribution. Biol Stud 10:163–186. 10.30970/sbi.1001.442

[CR111] Nikitin VV (2001) The new taxa of the genus *Viola* (Violaceae) in the East European flora. Bot J 86:134–147

[CR112] Nikitin VV (2002) Generis *Viola* L. (Violaceae) taxa Nova. Novitates Systematicae Plant Vascularium 34:125–129

[CR113] Nikitin VV (2003) Generis *Viola* L. (Violaceae) species et nothospecies novae. Novitates Systematicae Plant Vascularium 35:135–146

[CR115] Nobis M, Nowak A, Nobis A et al (2014) Contribution to the flora of Asian and European countries: new national and regional vascular plant records. Acta Bot Gallica 161:81–89. 10.1080/12538078.2013.871209

[CR114] Nobis M, Marciniuk J, Marciniuk P et al (2020) Contribution to the flora of Asian and European countries: new national and regional vascular plant records, 9. Turkish J Bot 44:455–480. 10.3906/bot-1908-41

[CR116] Novák P, Zukal D (2018) *Galium divaricatum* Pourr. ex Lam. (Rubiaceae) – a new species for the flora of Ukraine. Acta Bot Croatica 77:193–196. 10.2478/botcro-2018-0008

[CR117] Novikov A, Sup-Novikova M, Pachschwöll C (2020) *Stellaria ruderalis* M. Lepší, P. Lepší, Z. Kaplan et P. Koutecký, a new species record for the flora of Ukraine. Webbia J Plant Taxonomy Geogr 75:355–358. 10.36253/jopt9613

[CR118] Olshanskyi IG, Orlov OO (2013) *Juncus dichotomus* Elliott (Juncaceae), a new alien species for the flora of Ukraine. Ukrainian Bot J 70:769–771

[CR119] Orlov OO (2008) The first find of *Trifolium spryginii* Belyaeva & Sipliv. (Fabaceae) in Ukraine. Ukrainian Bot J 65:871–875

[CR120] Orlov OO, Gubar LM (2009) The first cases of becoming wild of *Macleaya cordata* (Willd.) R. Br. (Papaveraceae) in Ukraine. Ukrainian Bot J 66:550–553

[CR121] Orlov OO, Iakushenko DM (2013) *Lemna turionifera* Landolt (Araceae), a new species for the flora of Ukraine. Ukrainian Bot J 70:224–231

[CR123] Orlov OO, Shevera MV (2020) *Ionoxalis tetraphylla* (Oxalidaceae), a new ephemerophyte in the Ukrainian flora. Chornomorski Bot J 16:282–289. 10.32999/ksu1990-553X/2020-16-4-1

[CR124] Orlov OO, Shevera MV (2021) *Rudbeckia fulgida* (Asteraceae), a new ergasiophygophyte of Ukrainian flora. Biol Syst 13:100–104

[CR125] Orlov OO, Shevera MV, Bronskov OI (2014) *Impatiens balfourii* (Balsaminaceae), a new alien species of the Ukrainian flora. Ukrainian Bot J 71:45–49

[CR122] Orlov OO, Iakushenko DM, Májeková J et al (2019) *Galeopsis angustifolia* (Lamiaceae), a new alien species in the flora of Ukraine. Ukrainian Bot J 76:542–547. 10.15407/ukrbotj76.06.542

[CR126] Orlov OO, Shynder OI, Vorobjov EO, Gryb OV (2022) New floristic finds in the Forest-Steppe part of Zhytomyr region. Ukr Bot J 79:6–26. 10.15407/ukrbotj79.01.006

[CR127] Ostapko VM (2018) A new species of Milk Vine – *Vincetoxicum svetlanae* Ostapko. Industrial Bot Proc 18:4–9

[CR128] Ostapko VM (2020) New species of bedstraw – *Galium ×jubilaeare* Ostapko (Rubiaceae). Industrial Bot Proc 20:4–7

[CR129] Ostapko VM, Boyko AV, Mosyakin SL (2010) Vascular plants of the south-east of Ukraine. Noulidzh, Donetsk

[CR130] Panchenko SM, Mosyakin SL (2005) *Axyris amaranthoides* L. (Chenopodiaceae Vent.) – a new adventive species in the flora of Ukraine. Ukrainian Bot J, 62:213–217

[CR131] Parnikoza IYu, Celka Z (2016) *Botrychium simplex* E. Hitchc. (Ophioglossaceae) – a new species for the native flora of Ukraine. Biodivers Res Conserv 43:7–12. 10.1515/biorc-2016-0015

[CR132] Pavlov VV (2001) New species of the flora of Ukraine – *Potamogeton filiformis* Pers. (Potamogetonaceae). Ukrainian Bot J 58:610–611

[CR133] Pennisi E (2021) Itching to discover a new species? Follow this map. Science. 10.1126/science.abi644034914527

[CR134] Peregrym M, Koopman J (2023) *Carex × takhtadjanii* (*Carex diluta* × *C. distans*; Cyperaceae), a new hybrid for the flora of Ukraine. Hacquetia 22:91–96. 10.2478/hacq-2022-0011

[CR135] Peregrym M, Kuzemko A (2010) New infrageneric hybrid in the flora of Ukraine × *Dactylocamptis uechtritziana *(Hausskn.) M.Peregrym et Kuzemko, comb. nov. (Orchidaceae). Ukrainian Bot J 67:655–662

[CR136] Peregrym M, Kuzmichova O, Konaikova V (2016) *Veronica cardiocarpa* (Kar. & Kir.) Walp. (Plantaginaceae Juss.) in the O.V. Fomin Botanical Garden (Kyiv, Ukraine). Bull Taras Shevchenko Natl Univ Kyiv Ser Introduction Conserv Plant Divers 34:26–28

[CR137] Peregrym Yu, Bronskov O, Peregrym M (2013) *Astragalus calycinus* M. Bieb. (Fabaceae), a new species in the flora of Ukraine. Ukrainian Bot J 70:642–645

[CR138] Peterson A, Harpke D, Peruzzi L et al (2009) Hybridization drives speciation in *Gagea* (Liliaceae). Plant Syst Evol 278:133–148. 10.1007/s00606-008-0102-3

[CR139] Petryk SP (1992) Synantropic flora of the sea ports of the North-Western Black Sea Area. The dissertation on a scientific degree of the candidate of biological sciences on a specialty Botany, I.I. Mechnykov Odesa National University

[CR140] Plantarium https://www.plantarium.ru. Accessed 23 November 2025

[CR141] Plants of the World Online https://powo.science.kew.org. Accessed 23 November 2025

[CR142] Podda L, Lallai A, Calvia G et al (2025) Alien plants in the Hortus Botanicus Karalitanus: current and future threats to the biodiversity of Sardinia, Italy. J Zoological Bot Gardens 6:27. 10.3390/jzbg6020027

[CR143] Popova ОМ (2015) Finds of *Cephalanthera damasonium* and *Platanthera × hybrida* (Orchidaceae) in National Nature Park Tuzlovski Limany, the status of their populations and conservation perspectives. Ukrainian Bot J 72:357–363

[CR144] Prots B (2009) *Epipactis albensis* Nováková et Rydlo. In: Didukh YP (ed) The Red Data Book of Ukraine. Plant Kingdom. Globalconsulting, Kyiv, p 175

[CR145] PubMed https://pubmed.ncbi.nlm.nih.gov. Accessed 23 November 2025

[CR146] Punina YO, Mordak YV, Timukhin IN, Litvinskaja SA (2010) Synopsis of notospecies of the genus *Paeonia* L. (Paeoniaceae) of the Caucasus and Crimea. Novitates Systematicae Plant Vascularium 42:120–131

[CR150] Raab-Straube EV, Raus T (eds) (2019b) Euro + Med-Checklist Notulae, 10. Willdenowia 49:95. 10.3372/wi.49.49111

[CR149] Raab-Straube EV, Raus T (eds) (2019a) Euro + Med-Checklist Notulae, 11. Willdenowia 49:421. 10.3372/wi.49.49312

[CR148] Raab-Straube EV, Raus T (eds) (2017) Euro + Med-Checklist Notulae, 8. Willdenowia 47:293–309. 10.3372/wi.47.47311

[CR147] Raab-Straube EV, Raus T (eds) (2015) Euro + Med-Checklist Notulae, 5. Willdenowia 45:449–464. 10.3372/wi.45.45312

[CR155] Raab-Straube EV, Raus T (eds) (2024) Euro + Med-Checklist Notulae, 17. Willdenowia 54:5–45. 10.3372/wi.54.54101

[CR154] Raab-Straube EV, Raus T (eds) (2023) Euro + Med-Checklist Notulae, 16. Willdenowia 53:57–77. 10.3372/wi.53.53104

[CR151] Raab-Straube EV, Raus T (eds) (2020) Euro + Med-Checklist Notulae, 12. Willdenowia 50:305. 10.3372/wi.50.50214

[CR153] Raab-Straube EV, Raus T (eds) (2021b) Euro + Med-Checklist Notulae, 13. Willdenowia 51:. 10.3372/wi.51.51112

[CR152] Raab-Straube EV, Raus T (eds) (2021a) Euro + Med-Checklist Notulae, 14. Willdenowia 51:. 10.3372/wi.51.51304

[CR156] Romo A, Didukh YP, Boratynski A (2004) *Thesium* (Santalaceae) in Crimea, Ukraine. Ann Botanici Fennici 41:273–281

[CR157] Rostanski K, Tokhtar VK, Shevera MV (1997) New species of the genus *Oenothera* L. for the flora of Ukraine. Ukrainian Bot J 54:173–177

[CR158] Rudenko LH (ed) (2007) National atlas of Ukraine. DNVP Kartografiya, Kyiv

[CR159] Ryff LE (2011) *Cyclospermum leptophyllum* (Pers.) Sprague ex Britton et P. Wilson (Apiaceae) is a new adventive plant for the territory of Ukraine. Ukrainian Bot J 68:581–584

[CR160] Ryff LE (2013) *Asplenium lepidum* C. Presl subsp. *Haussknechtii* (Godet et Reut.) Brownsey (Aspleniaceae), a new taxon of ferns in the flora of Eastern Europe. Ukrainian Bot J 70:519–521

[CR161] Ryff LE, Svirin SA, Yevseenkov PE, Voloshyn RR (2013) *Avena clauda* (Poaceae) – a new species of flora in Eastern Europe. Bot J 98:1282–1287

[CR162] Ryff LE, Svirin SA, Yevseyenkov PE (2023) *Gelasia villosa* Cass. (Asteraeeae), a representative of a new genns for the flora of the Crimea. Bull State Nikitsky Bot Garden 146:84–95. 10.36305/0513-1634-2023-146-84-95

[CR163] Santini A, Maresi G, Richardson DM, Liebhold AM (2023) Collateral damage: military invasions beget biological invasions. Front Ecol Environ 21:469–478. 10.1002/fee.2640

[CR164] Scopus. https://www.scopus.com. Accessed 23 November 2025

[CR165] Sennikov AN, Tikhomirov VN (2024a) Atlas florae Europaeae notes, 33. Taxonomic synopsis of East European species of the *Cytisus ratisbonensis* group (Fabaceae). Phytokey 238:157–197. 10.3897/phytokeys.238.118031 10.3897/phytokeys.238.118031 PMC10907954 38435133

[CR166] Sennikov AN, Tikhomirov VN (2024b) Atlas florae Europaeae notes, 34. Distributions and two conservation profiles of East European species of the *Cytisus ratisbonensis* group (Fabaceae). Biodivers Data J 12:e118034. 10.3897/BDJ.12.e118034 38434751 10.3897/BDJ.12.e118034 PMC10907953

[CR168] Seregin AP (2004) New and rare species of the genus *Allium* L. (Alliaceae) of the Crimean flora and some questions of taxonomy of representatives of the genus. Byulleten’ Moskovskogo Obshchestva Ispytatelei Prirody Otdel Biologicheskii 109:43–47

[CR169] Seregin AP (2008) Contribution to the vascular flora of the Sevastopol area (the Crimea): a checklist and new records. Flora Mediterranea 18:5–81

[CR170] Seregin AP (2009) *Parentucellia* (Scrophulariaceae), a new genus for Eastern Europe, and notes on the flora of the Sevastopol area. Bot J 94:892–895

[CR171] Seregin AP (2010) Two new alien species for the flora of Eastern Europe from the Crimea. Byulleten’ Moskovskogo Obshchestva Ispytatelei Prirody Otdel Biologicheskii 115:79

[CR167] Seregin AP (2012) *Allium tarkhankuticum* (Amaryllidaceae), a new species of section Oreiprason endemic to the Crimean steppe. Ukraine Phytotaxa 42:9. 10.11646/phytotaxa.42.1.2

[CR172] Seregin AP, Anačkov G, Friesen N (2015a) Molecular and morphological revision of the *Allium saxatile* group (Amaryllidaceae): geographical isolation as the driving force of underestimated speciation: A revision of the *Allium saxatile* group. Bot J Linn Soc 178:67–101. 10.1111/boj.12269

[CR173] Seregin AP, Yevseyenkov PE, Svirin SA, Fateryga AV (2015b) Second contribution to the vascular flora of the Sevastopol area (the Crimea). Wulfenia 22:33–82

[CR174] Shalimov AP (2022) First record of *Selaginella kraussiana* (Kunze) A. Braun in Western Ukraine (Eastern Europe). Turczaninowia 25:221–225. 10.14258/turczaninowia.25.3.21

[CR175] Shevera MV, Orlov OO, Volutsa OD, Kish RY (2020) *Rudbeckia triloba* (Asteraceae), a new alien species in Ukrainian flora. Chornomorski Bot J 16:135–143. 10.32999/ksu1990-553X/2020-16-2-3

[CR176] Shipunov AB (1996) Some new and rare species of the genus *Plantago* L. (Plantaginaceae) from the differen regions of the former USSR. Byulleten’ Moskovskogo Obshchestva Ispytatelei Prirody Otdel Biologicheskii 101:67–69

[CR177] Shipunov AB (1997) New data about distribution of some species of *Plantago* L. and *Psyllium* Mill. (Plantaginaceae) in Eastern Europe. Byulleten’ Moskovskogo Obshchestva Ispytatelei Prirody Otdel Biologicheskii 102:64

[CR178] Shipunov AB (2000) Species of the genera *Plantago* L. and *Psyllium* Mill. (Plantaginaceae) in the flora of Eastern Europe. Novitates Systematicae Plant Vascularium 32:139–152

[CR179] Shiyan NM (2018) *Drosera filiformis* Raf. – an example of the deliberate introduction of a species in Kyiv Polissia. Collection of scientific papers. PP Ruta, Zhytomyr, pp 375–376

[CR180] Shiyan NM, Orlov OO, Iakushenko DM (2024) *Wolffia globosa* (Araceae s.l. / Lemnaceae s.str.), a new aquatic alien species in the flora of Ukraine. Ukrainian Bot J, 81:40–51. 10.15407/ukrbotj81.01.040

[CR181] Shumilova AV (2002) *Artemisia latifolia* Ledeb. (Asteraceae) – a new species for the flora of Ukraine. Ukrainian Bot J 59:724–728

[CR182] Shumilova AV (2014) *Calligonum aphyllum* (Polygonaceae), an interesting find in the historical collection of I.K. Boyko. Ukrainian Bot J 71:250–253

[CR183] Shynder OI (2019) Spontaneous flora of M.M. Gryshko National Botanical Garden of the National Academy of Sciences of Ukraine (Kyiv). 3. Escaped plants. Plant Introduction 83:14–29. 10.5281/zenodo.3404102

[CR188] Shynder OI, Negrash Y, Glukhova S et al (2020) Alien species of the genus *Lonicera* (Caprifoliaceae) in the flora of right-bank Ukraine. NaUKMA Res Papers Biology Ecol 3:58–65. 10.18523/2617-4529.2020.3.58-65

[CR185] Shynder OI, Doiko NM, Glukhova SA et al (2022a) New information about the flora of plant introduction institutions in Kyiv and Bila Tserkva (Kyiv region). Chornomorski Bot J 18:25–51. 10.32999/ksu1990-553X/2022-18-1-2

[CR186] Shynder OI, Kolomiychuk VP, Melezhyk OV (2022b) Spontaneous flora of O.V. Fomin Botanical Garden of Taras Shevchenko National University of Kyiv, Ukraine. Environmental & Socio-economic Studies 10:38–56. 10.2478/environ-2022-0004

[CR187] Shynder OI, Kostruba T, Chorna G, Kolomiychuk V (2022c) New and additional information on the flora of the Middle Dnieper. NaUKMA Res Papers Biology Ecol 5:64–75. 10.18523/2617-4529.2022.5.64-75

[CR184] Shynder OI, Davydov DA, Olshanskyi IG et al (2024) New floristic records in Kyiv City and its environs. Ukrainian Bot J 81:100–144. 10.15407/ukrbotj81.02.100

[CR189] Šlapeta J (2013) Ten simple rules for describing a new (parasite) species. Int J Parasitology: Parasites Wildl 2:152–154. 10.1016/j.ijppaw.2013.03.005 10.1016/j.ijppaw.2013.03.005 PMC3862503 24533329

[CR190] Sołtys-Lelek A, Oliіar H (2016) The species of the genus *Crataegus* L. in the National Nature Park ‘Podilskyi Tovtry’ (Podolian Hills, Western Ukraine). Biodivers Res Conserv 44:25–34. 10.1515/biorc-2016-0019

[CR191] Sova TV, Mosyakin SL (2002) *Vulpia octoflora* (Walt.) Rydb. (Poaceae) is a new adventive species of flora of Ukraine. Ukrainian Bot J, 59:542–546

[CR192] Steen B, Adde A, Schlaepfer MA et al (2024) Distributions of non-native and native plants are not determined by the same environmental factors. Ecol Sol Evid 5:e12374. 10.1002/2688-8319.12374

[CR193] Štěpánek J, Kirschner J (2014) A revision of names in *Taraxacum* sect. *Erythrocarpa* and *T*. sect. *Erythrosperma* (*Asteraceae*: *Cichorieae*) published by C. E. Sonck from Greece, with nomenclatural comments. Willdenowia 44:137–144. 10.3372/wi.44.44114

[CR194] Štěpánek J, Kirschner J (2018) Taxonomic revision of selected species in *Taraxacum* sect. *Erythrosperma* (*Asteraceae*: *Cichorieae*) from the E Mediterranean region. Willdenowia 48:365–369. 10.3372/wi.48.48304

[CR195] Štěpánek J, Kirschner J (2022a) *Taraxacum* sect. *Erythrocarpa* in Europe in the Alps and eastwards: A revision of a precursor group of relicts. Phytotaxa 536:7–52. 10.11646/phytotaxa.536.1.2

[CR196] Štěpánek J, Kirschner J (2022b) A distinctive group of species allied to *Taraxacum danubium* (*T.* sect. *Erythrosperma*, Compositae-Crepidinae): a taxonomic revision. Folia Geobot 57:269–301. 10.1007/s12224-023-09425-6

[CR197] Štěpánek J, Kirschner J, Uhlemann I (2023) A survey of the oreophytic species of *Taraxacum* in the Carpathians reveals a very limited overlap with the flora of the Alps. Preslia 95:475–591. 10.23855/preslia.2023.475

[CR198] Takács A, Zsólyomi T, Molnár VA et al (2020) Evidence of hybridization between *Galatella villosa* and *G. linosyris*, and a taxonomic reap-praisal of the hybrid *G. ×subvillosa*. Preslia 92:375–390

[CR199] Tatanov IV (2003a) On the distribution of *Bolboschoenus glaucus* (Cyperaceae) in the East Europe. Bot J 88:106–111

[CR200] Tatanov IV (2003b) De speciebus *Bolboschoenum desoulavii* (Drob.) A.E. Kozhevnikov et *Bolboschoenum yagara* (Ohwi) Y.C. Yang et M. Zhan (Cyperaceae). Novitates Systematicae Plant Vascularium 35:51–62

[CR202] The European Nature Information System https://eunis.eea.europa.eu. Accessed 23 November 2025

[CR201] The Creative Commons Attribution–ShareAlike 3.0 license. Accessed 23 November 2025 https://creativecommons.org/licenses/by-sa/3.0

[CR203] Thomson JA (2004) Towards a taxonomic revision of *Pteridium*. (Dennstaedtiaceae) Telopea 10:793–803

[CR204] Tikhomirov VN (2002) The genus *Pilosella* Hill. (Asteraceae) in the flora of Ukraine. I. *Pilosella aurantiaca* (L.) F. Schultz et Sch. Bip. and hybrids with participation of this species. Ukrainian Bot J 59:267–271

[CR205] Tikhomirov VN (2015) *Chamaenerion danielsii* (D.Löve) Czerep. (Onagraceae) in the flora of Eastern Europe. Novitates Systematicae Plant Vascularium 46:147–156

[CR206] Travnicek В (1998) Notes on the taxonomy of *Pseudolysimachion* sect. *Pseudolysimachion* (Scrophulariaceae) in Europe. I. *P. incanum* and *P. spicatum*. Preslia 70:193–223

[CR207] TUBS (2025) The map Ukraine, administrative divisions. https://upload.wikimedia.org/wikipedia/commons/f/f2/Ukraine%2C_administrative_divisions_-_Nmbrs.svg. Accessed 23 November 2025

[CR208] Tyshchenko OV, Tyshchenko VM, Kucheryava LF (2013) Finding of *Celastrus scandens* L. (Celastraceae) in the Rizany Yar protected tract (Cherkasy Region). Ukrainian Bot J 70:646–648

[CR209] Tzvelev NN (2000) Notes on some genera of the family Caryophyllaceae *sensu lato* in Eastern Europe. Novitates Systematicae Plant Vascularium 32

[CR210] Tzvelev NN (2001a) De generibus tribus *Sileneae* DC. (Caryophyllaceae) in Europa Orientali. Novitates Systematicae Plant Vascularium 33:90–113

[CR219] Tzvelev NN (ed) (2001b) Flora Europae Orientalis. Mir i semia, Saint Petersburg

[CR211] Tzvelev NN (2002) On some genera of Caryophyllaceae family in East Europe. Bot J 87:120–130

[CR212] Tzvelev NN (2003a) De genere *Dryopteris* Adans. (Dryopteridaceae) in Europa Orientali. Novitates Systematicae Plant Vascularium 35:7–20

[CR213] Tzvelev NN (2003b) De Brassicaceis nonnullis Europae orientalis. Novitates Systematicae Plant Vascularium 35:95–108

[CR220] Tzvelev NN (ed) (2004) Flora Europae orientalis. Oficina editoria KMK, Moscow-Saint Petersburg

[CR214] Tzvelev NN (2005) The genus *Pteridium* (Hypolepidaceae) in the Eastern Europe and the Northern Asia. Bot J 90:891–896

[CR215] Tzvelev NN (2006) Brief summary of cereals (Poaceae) of Eastern Europe: the beginning of the system (tribes Bambuseae – Bromeae). Novitates Systematicae Plant Vascularium 38

[CR216] Tzvelev NN (2007) De genere *Epilobium* L. (Onagraceae) in Europa orientali. Novitates Systematicae Plant Vascularium 39

[CR217] Tzvelev NN (2009) On the species of the section *Stenopoci* Dumort. of genus Bluegrass (*Poa* L., Poaceae) in Eastern Europe. Novitates Systematicae Plant Vascularium 41:18–52

[CR218] Tzvelev NN (2012) On species of the genus *Melica* sect. *Melica* (Poaceae) in Russia. Bot J 97:252–257

[CR221] Tzvelev NN, Geltman DV (eds) (2012) Conspectus florae Europae orientalis. Tovarishchestvo nauchnykh izdaniĭ KMK, Sankt-Petersburg/Moskow

[CR222] Umanets OYu (2000a) *Elytrigia striatula* (Poaceae), a new species for Eastern Europe. Bot J 85:129–130

[CR223] Umanets OYu (2000b) A new species of the genus *Helichrysum* (Asteraceae) from the Black Sea coast. Bot J 85:112–113

[CR224] Vasjukov VM (2016) New species of *Thymus* L (Lamiaceae). Novitates Systematicae Plant Vascularium 108–115. 10.31111/novitates/2016.47.108

[CR225] Vasyljeva TV, Kovalenko SG (2000) New species in the adventive flora of some black sea cities. Ukrainian Bot J 57:544–547

[CR226] Web of Science https://www.webofscience.com. Accessed 23 November 2025

[CR227] Wolf PG, Rowe CA, Kinosian SP et al (2019) Worldwide relationships in the fern genus *Pteridium* (bracken) based on nuclear genome markers. Am J Bot 106:1365–1376. 10.1002/ajb2.1365 31545874 10.1002/ajb2.1365 PMC6856829

[CR228] Yena AV (2008) *Caulinia graminea* (Delile) Tzvelev (Najadaceae) – a new species of Ukrainian flora. Ukrainian Bot J 65:73–76

[CR230] Yena AV, Shevera MV (2011) Critical notes on systematic of pinophyta in Ukrainian flora. Chornomorski Bot J 7:113–118

[CR229] Yena AV, Korzhenevsky VV, Ryff LE (2006) *Bifora testiculata* (L.) Spreng. (Apiaceae) - a new species of flora of Eastern Europe and other floristic finds in the Crimea. Bull Main Bot Garden 190:102–108

[CR231] Yena AV, Yevseenkov PE, Svirin SA (2011) *Sagina maritima* G. Don (Caryophyllaceae), a new species for the flora of Ukraine. Ukrainian Bot J 68:191–194

[CR232] Yeremko IO (1997) *Rumex cristatus* DC. (Polygonaceae) in Ukraine. Ukrainian Bot J 54:278–279

[CR233] Zasenko OE, Stebelsky I, Makuch A et al (2024) Ukraine. Britannica. https://www.britannica.com/place/Ukraine Accessed 1 Feb 2024

[CR234] Zavjalova LV (2008) *Aizopsis aizoon* (L.) Grulich (Crassulaceae) – a new ergasiophyte in the flora of Ukraine. Ukrainian Bot J 65:876–881

[CR235] Zhmud OI, Zhmud OV (2010) A new species for the flora of Ukraine – *Solanum retroflexum* Dunal (Solanaceae) on the territory of the Danube biosphere reserve and the issue of further synanthropization of the flora of the Kili Danube delta. Nat Almanac, 68–77

[CR236] Zhou S, Dong W, Chen X et al (2014) How many species of bracken (*Pteridium*) are there? Assessing the Chinese brackens using molecular evidence. Taxon 63:509–521. 10.12705/633.9

[CR237] Zielinski J (2004) The genus *Rubus* (Rosaceae) in Poland. Pol Bot Stud 16:1–300

[CR238] Zvyagintseva KO (2015) An annotated checklist of the urban flora of Kharkiv. V.N. Karazin Kharkiv National University, Kharkiv

