# Supplementary material for: Drivers of floristic discovery in a temperate flora: insights from three decades of vascular plant records in Ukraine
Source: Bot Stud. 2026 Mar 6;67:5. doi: 10.1186/s40529-026-00492-4 (PMC12965962; doi:10.1186/s40529-026-00492-4)
Supplement: Supplementary file 1 — Supplementary Material 1 [file 40529_2026_492_MOESM1_ESM.docx]

Supplementary material. The list of new species and subspecies of vascular plants reported for the first time for the flora of Ukraine during 1997-2024

| № | Taxon (according to original reports) | Administrative region of the finding/s | Habitat | Dates of the collection | Native (N) or alien (A) species | Notes |
| --- | --- | --- | --- | --- | --- | --- |
| **LYCOPODIOPSIDA** | | | | | | |
| ***Selaginellaceae* Willk.** | | | | | | |
| 1 | ***Selaginella kraussiana* (Kunze) A.Braun** | Transcarpathian region, Vinogradov city, southern slopes of Mount Baba-kyu (Chorna Mountain) | in dense vegetation cover | 18.08.1959 | A | (Shalimov, 2022);  POWO: ***S. kraussiana*** |
| **POLYPODIOPSIDA** | | | | | | |
| ***Aspleniaceae* Newman** | | | | | | |
| 2 | ***Asplenium caucasicum* (Fraser-Jenkins et Lovis) Viane** | Crimea, Alushta vicinity, northern macroslope of the Ayu-Dag mountain, 44°33′45,49′′N, 34°19′52,24′′E | alt. 500 m a.s.l | 20.05.2019 | N | (Kechaykin et al., 2020);  POWO: ***A. septentrionale* (L.) Hoffm. subsp. *caucasicum* Fraser-Jenk. & Lovis** |
| 3 | ***Asplenium fontanum* (L.) Bernh.** | Lviv region, Skole | - | - | N | (Bezsmertna et al., 2012);  POWO: ***A. fontanum*** |
| 4 | ***Asplenium lepidum* C. Presl subsp. *haussknechtii* (Godet et Reut.) Brownsey** | Crimea, Crimean Nature Reserve, Kozmo-Demyaniv Monastery | old wall | 09.2011 | N | (Ryff, 2013);  POWO: ***A. lepidum* subsp. *haussknechtii*** |
| ***Dennstaedtiaceae* Lotsy** | | | | | | |
|  | *Pteridium* *aquilinum* (L.) Kuhn subsp. *japonicum* (Nakai) Á.Löve & D.Löve | Kyiv | - | - | N | (Zhou et al., 2014);  POWO: *P. latiusculum* (Desv.) Hieron. subsp. *japonicum* (Nakai) Fraser-Jenk.  It was confirmed later that the analyzed sample from Ukraine belong to *P. aquilinum* subsp. *pinetorum* (C.N. Page & R.R. Mill) J.A. Thomson (Wolf et al., 2019) |
| 5 | ***Pteridium aquilinum* subsp. *pinetorum* (C.N. Page & R.R. Mill) J.A. Thomson** | Northern Ukraine | - | - | N | (Thomson, 2004);  POWO: ***P. pinetorum* C.N. Page & R.R. Mill**  It is suggested considering as a subspecies based on modern molecular data (Thomson, 2004; Wolf et al., 2019), despite of some alternative opinions (Gureyeva & Page, 2008; Tzvelev, 2005) |
|  | *Pteridium* *latiusculum* (Desv.) Hieron. ex Fries | Forest and forest-steppe regions | - |  | N | (Tzvelev, 2005);  POWO: *P. latiusculum*  This taxon was noted mistakenly, because according to the lastest data this species is considered as a subspecies (*P. aquilinum* (L.) Kuhn subsp. *latiusculum* (Desv.) Hultén) distributed within North America (Thomson, 2004; Wolf et al., 2019) |
| ***Equisetaceae* Rich. ex DC.** | | | | | | |
| 6 | ***Equisetum* ×*moorei* Newman** (= *E. hyemale* L. × *E. ramosissimum* Desf.) | 1) Kyiv, Holosiivskyi National Nature Park, 50.262368ºN, 30.571964ºE;  2) Poltava region, Poltava, behind Zaturyne railway crossing, 49.607148ºN, 34.620545ºE;  3) Crimea, Simferopolskyi district, Chaykovske village, 44.827994ºN, 34.327386ºE, 44.832529ºN, 34.354171ºE | 1) on a sedge swamp on the shore of the Shaparnia lake, near the railway;  2) on a hill near a pine forest;  3) - | 1) 06.03.2016; 2) 26.11.2020, 01.12.2020;  3) 31.07.2020, 12.12.2020 | N/A | (Shynder et al., 2024);  POWO: ***E.*×*moorei*** |
| ***Ophioglossaceae* Martinov** | | | | | | |
| 7 | ***Botrychium simplex* E. Hitchc.** | the surroundings of Kharkiv, on the Kharkiv River | moist sandy sites in the community with *Juncus ericetorum* Pollich (currently *J. capitatus* Weigel) | 12.06.1828 | N | (Parnikoza & Celka, 2016);  POWO: ***B. simple*** |
| ***Polypodiaceae* J.Presl & C.Presl** | | | | | | |
| 8 | ***Dryopteris* × *euxinensis* Fras.-Jenk. et Corley** (=*D. filix-mas* (L.) Schott × *D. caucasica* (A.Br.) Fras.-Jen) | Crimea, Monastyrskyi range | - | 08.08.1946 | N | (Tzvelev, 2003b);  POWO: ***D.*×*euxinensis*** |
| 9 | ***Dryopteris remota* (A.Br. ex Doell) Druce** | Ivano-Frankivsk region, near Yaremche | mountain forest, 800 m above s.l. | 08.09.1948 | N | (Tzvelev, 2003b);  POWO: ***D. remota*** |
| 10 | ***Dryopteris villarii* (Bellardi) Woynar ex Schinz et Thell.** | Crimea, Crimean Nature Reserve, Babuhan-Yaila, N 44°38'13.22", E 34°18'25.87" and N 44°38'06.08", E 34°18'10.98" | on stony limestone screes in the niches between the depths, alt. 1300 m | 09.07.2011 | N | (Bezsmertna, 2011);  POWO: ***D. villarii*** |
| **GYMNOSPERMAE** | | | | | | |
| ***Cupressaceae* Gray** | | | | | | |
| 11 | ***Platycladus orientalis* (L.) Franco** | Crimea: 1) near Bakchysarai; 2) near Kurortne;  3) Uzhhorod;  4) Khmelnytskyi region, Kamianets’-Podilskyi | - | - | A | it is considered as a naturalized species in Ukraine for the first time (Yena & Shevera, 2011);  POWO: ***P. orientalis*** |
| ***Pinaceae* Spreng. ex F.Rudolphi** | | | | | | |
| 12 | ***Pinus uliginosa* G.E. Neumann ex Wimm** | Ivano-Frankivsk region, Kalush district, near Osmoloda village, Gorgany Mts., N 48°40’33.2” , E 23º55’19.4” | oligotrophic peat bog with stunted trees | 06.2008 | N | (Jasinska et al., 2009);  POWO: ***P. uncinata* Ramond ex DC. subsp. *uliginosa* (G.E.Neumann) Businský** |
| **ANGIOSPERMAE** | | | | | | |
| **MAGNOLIIDS** | | | | | | |
| ***Aristolochiaceae* Juss.** | | | | | | |
| 13 | ***Aristolochia macrophylla* Lam.** | Kyiv, Stolychne Highway, sanatorium "Koncha-Zaspa", 50º17'42"N, 30º35'21"E | in park | 10.2011 | A | first cases of escape from cultivation (Burda, 2014);  POWO: ***A. macrophylla*** |
| **MONOCOTS** | | | | | | |
| ***Amaryllidaceae* J. St.-Hil.** | | | | | | |
| 14 | ***Allium × agarmyschicum* N.  Friesen &Seregin** (= *A. tarkhankuticum* Seregin × *A. marschallianum* Vved.) | Crimea, vicinity of Staryi Krym, ascent to Mount Velykyi Agarmysh from west along the ridge: 45°01′40′′N, 35°01′55′′E; 45°01′40′′N, 35°02′20′′E; 45°01′45′′N, 35°02′30′′E | petrophytic glades, elev. 640 m; shelveson rocks with herbs on the edge of xeric oak forest,elev. 700 m; lime-stone outcrops covered by small debris, elev. 710 m | 18.08.2012 | N | first described for science (Seregin, Anačkov, et al., 2015);  POWO: ***A. × agarmyschicum*** |
| 15 | ***Allium altissimum* Regel** | Kyiv, M.M. Gryshko National Botanical Garden of the National Academy of Sciences of Ukraine, exposition plots “Middle Asia” and “Rock Mountain” | - | - | A | first cases of escape from cultivation (Shynder, 2019). Later, a new case was noted from Zhytomyr region (Orlov et al., 2022);  POWO: ***A. altissimum*** |
| 16 | ***Allium caeruleum* Pall.** | Kyiv, M.M. Gryshko National Botanical Garden of the National Academy of Sciences of Ukraine, throughout the territory | - | - | A | first cases of escape from cultivation (Shynder, 2019);  POWO: ***A. caeruleum*** |
| 17 | ***Allium carolinianum* Redouté** | Kyiv, M.M. Gryshko National Botanical Garden of the National Academy of Sciences of Ukraine | - | - | A | first cases of escape from cultivation (Shynder, 2019);  POWO: ***A. carolinianum*** |
| 18 | ***Allium globosum* M. Bieb. ex Redouté** | Crimea, near Alupka and Yalta | - | 1912 | N | (Seregin, 2004);  POWO: ***A. saxatile*** M.Bieb. |
| 19 | ***Allium pallens* L. subsp. *coppoleri* (Tineo) Seregin** var. *pseudooleraceum* Seregin | Crimea, [Yalta], Artek | terrace over the sea | 19.08.1982 | N | first described for science (Seregin, 2004);  POWO: ***A. coppoleri*** Tineo |
| 20 | ***Allium ramosum* L.** | Kyiv, M.M. Gryshko National Botanical Garden of the National Academy of Sciences of Ukraine | - | - | A | first cases of escape from cultivation (Shynder, 2019);  POWO: ***A.  ramosum*** |
| 21 | ***Allium tarkhankuticum* Seregin** | Crimea:  1) Saky District, 45°22′50″N, 33°06′30″E, S shore of Lake Donuzlav, W outskirts of Novoozerne;  2) Chornomorske District: between Aqmeçit [Chornomorske] and Qunan [Krasnosilske];  3) Tarkhankut [Tarkhan Qut] Peninsula, Dzhangul locality near Olenivka;  4) Dzhangul Coast;  5) Tarkhankut Peninsula, Dzhangul locality;  6) Tarkhankut Cape, ca. 5 km to S from Olenivka;  7) Tarkhankut Peninsula, 10 km to N from Olenivka, Dzhangul locality;  8) 45°23ʹ30ʺN, 32°29ʹ50ʺE, 3 km to WNW from Olenivka;  9) 45°24ʹ40ʺN, 32°30ʹ00ʺE, 4.5 km to NW from Olenivka;  10) Saky District: Caǧa Quşçı [Okhotnykove], on the N tip of Lake Sasıq Sıvaş;  11) S shore of Lake Saky;  12) locus classicus, S shore of Lake Donuzlav, W outskirts of Novoozerne;  13) locus classicus, 45°22ʹ50ʺN, 33°06ʹ30ʺE, S shore of Lake Donuzlav, W outskirts of Novoozerne;  14) Pervomayske [Kurman] District: near Boz [Tykhonivka];  15) Aybar [Voykove];  16) Krasnogvardiyske [Kurman] District: near Tilençi [Dokuchayeve];  17) Simferopol District: to N from Simferopol, Baǧça Eli [part of Simferopol] – Ana Eli [part of Zahirske];  18) Bilohirske District: near Boçala [Bochala / Udarne] | 1) petrophytic steppe, alt. 10 m;  2) stony steppe;  3) -  4) petrophytic steppe;  5) -  6) steppe above coastal cliff;  7) steppe;  8) steppe (*Artemisia santonica*, *Stipa capillata*, *Festuca valesiaca*) on stony soil, elev. 20 m;  9) sparse petrophytic steppe (*Artemisia santonica*, *Stipa capillata*, *Festuca valesiaca*, *Thymus sp.*) near the sea, elev. 20 m;  10) stony slopes;  11) grass-and-wormwood steppe;  12) *Festuca valesiaca* steppe  13) petrophytic steppe (*Artemisia taurica*, *Festuca valesiaca*, *Agropyron cristatum*, etc.), elev. 10 m  14) virgin steppe;  15) virgin steppe;  16) feather grass steppe;  17) virgin stony (Sarmatian) plot;  18) steppe | 1) 19.08.2008;  2) 08.08.1943;  3) 12.10.1979;  4) 08.06.1982;  5) 17.08.1987;  6) 28.09.1987;  7) 18.08.2000;  19.08.2000;  8) 14.08.2008;  9)14.08.2008;  10) 06.09.1931;  11) 20.08.1935;  12) 15.09.2001;  13) 15.08.2008;  14) 24.08.1917;  15) 08.07.1922;  16) 07.07.1895;  17) 25.07.1922;  18) 24.08.1895 | N | first described for science (Seregin, 2012);  POWO: ***A. tarkhankuticum*** |
| 22 | ***Allium tuberosum* Rottler ex Spreng.** | Kyiv, M.M. Gryshko National Botanical Garden of the National Academy of Sciences of Ukraine | - | - | A | first cases of escape from cultivation (Shynder, 2019);  POWO: ***A. tuberosum*** |
| ***Araceae* Juss.** | | | | | | |
| 23 | ***Lemna turionifera* Landolt** | 1) Volyn region, Liubeshiv district, 1 km to N from Birky village, Tsyr River within the National Nature Park “Prypiat-Stokhid”;  2) Zhytomyr region, Yemilchyne district, Yemilchyne, Ubort River;  3) Chernihiv region, Mena district, 2 km to N from Komarivka village, drying system “Kyselivka” | Communities of *Lemnetea* de Bolós et Masclans 1955 | - | A | (Orlov & Iakushenko, 2013);  POWO: ***L. turionifera*** |
| 24 | ***Wolffia globosa* (Roxb.) Hartog & Plas** | Zhytomyr region: 1) Zhytomyr district, Khoroshiv village community, 1.4 km southeast of Radychi village, 50°40'22.51"N, 28°31'32.87" E;  2) Zvyahel district, Bronyky village community, 4.5 km north of Uzhachyn village, 50°39'47.57" N, 27°46'01.16" E | The association *Lemno-Spirodeletum polyrhizae* of the alliance *Lemnion minoris* of the class *Lemnetea*  1) open warm shallow water area;  2) in a shallow lake | 1) 11.07.2023;  2) 24.06.2023 | A | (Shiyan et al., 2024);  POWO: ***W. globosa*** |
| ***Asparagaceae* Juss.** | | | | | | |
| 25 | ***Muscari armeniacum* H.J.Veitch** | 1) Kyiv, M.M. Gryshko National Botanical Garden of the National Academy of Sciences of Ukraine;  2) Kyiv region, Bila Tserkva city, Arboretum “Olexandria” | - | 1) 21.04.2021;  2) 28.04.2021 | A | first cases of escape from cultivation (Shynder, Doiko, et al., 2022);  POWO: ***M. armeniacum*** |
| 26 | ***Ornithogalum navaschinii* Agap.** | Crimea: 1) from Sudak to Novyi Svit;  2) Koktebel (Planerske), under the Echki-Dag Mount;  3) over the Royal Bay in Novyi Svit | 1) dry slopes;  2) dry rocky slopes;  3) dry slopes | 1) 19.04.1972;  2) 24.04.1972;  3) 20.04.1972 | N | first described for science (Agapova, 1998);  POWO: ***O. navaschinii*** |
| 27 | ***Puschkinia scilloides* Adams** | Kyiv, M.M. Gryshko National Botanical Garden of the National Academy of Sciences of Ukraine | - | - | A | first cases of escape from cultivation (Shynder, 2019);  POWO: ***P. scilloides*** |
| ***Cyperaceae* Juss.** | | | | | | |
| 28 | ***Bolboschoenus glaucus* (Lam.) S.G. Smith** | Odesa region:1) Izmail;  2) Reni, Orlivka, in Danube River Valley;  Crimea: 3) Inkerman;  4) Koktebel;  5) Koktebel;  6) [Sevastopol] Baidarska Valley, near Chorna River;  7) Baidarska Valley, near Baibary-Khaitu stream;  8) Baidarska Valley, Orlyne;  9) [Bilohirskyi district] along Biyuk-Karasu River  10) Simferopol;  11) Eastern Crimea, [Sudak], Chalka River, near Echki-Dag  12) [Alushta], near Luchyste village;  13) Bakhchysarai district, Sadove village, on river Kacha;  14) [Sevastopol] Balaklava district, Ayazma tract | on swampy silt-sandy places, usually saline, and along the banks of reservoirs  1) -  2) meadows  3) -  4) -  5) pond  6) -  7) -  8) -  9) meadows;  10) -  11) -  12) -  13) -  14) - | 1) -  2) 28.07.1958;  3) VII.1897;  4) 06.07.1903;  5) 30.06.1912;  6) 12.07.1906;  7) 02.08.1906;  8) 24.05.1962;  9) 17.07.1924;  10) 04.06.1926;  11) 26.06.1929;  12) 05.07.1959;  13) 08.07.1968;  14) 10.06.1985 | N | (Tatanov, 2003b);  POWO: ***B. glaucus*** |
| 29 | ***Bolboschoenus yagara* (Ohwi) Y.C. Yang et M. Zhan** | 1) Sumy region, Shepetivka district, “Mykhailivska hora”;  2) Vinnytsia region, near Haisyn town;  3) near Poltava;  4) Luhansk region, near Starobilsk | wetlands | - | N | (Tatanov, 2003a);  POWO: ***B. yagara*** |
| 30 | ***Carex brunnescens* (Pers.) Poiret** | Sumy region, Stara Huta district, Stara Huta forestry, about 500 m from Stara Huta village, quarter #112 and #113 | derived communities of forest cultural phytocenoses of *Pinus sylvestris* L. |  | N | (Danylyk & Panchenko, 2001);  POWO: ***C. brunnescens*** |
| 31 | ***Carex ×takhtadjanii* Jac. Koopman & Więcław** (= *C. diluta* M. Bieb. × *C. distans* L.) | Luhansk region, in the River Aidar valley within Starobilsk town, 49.280051°N, 38.927307°E | wet saline meadows: *Bolboschoenetum maritime* Eggler 1933, *Scirpion maritimi* Dahl et Hadač 1941, *Bolboschoenetalia maritimi* Hejný in Holub et al. 1967, *Bolboschoenetea maritimi* Vicherek et Tx. in Tx. et Hül-busch 1971 | 13.06.2021 | N | (M. Peregrym & Koopman, 2022);  POWO: ***C. ×takhtadjanii*** |
| 32 | ***Schoenoplectus pungens* (Vahl) Palla** | Volyn region, Shatsk district, Smolyary-Svityazki village, Prybych Lake, N 51°24’47” , E 23º46’36 | coastal-aquatic vegetation with dominance of *S. pungens* | 27.06.2006 | N (?) | (Danylyk & Honcharenko, 2009);  POWO: ***S. pungens*** |
| 33 | ***Trichophorum alpinum* (L.) Pers.** | Volyn region, Shatsk district, 1.5 km to S from Melnyky village, N 51°31'95.1’’, E 23°56'18.6” | mesotrophic grass-moss bog: *Caricion lasiocarpae* Vanden Bergh. ap. Lebrun et al. 1949, *Scheuchzerietalia palustris* Nordh. 1937, *Sсheuchzerio-Caricetea nigrae* (Nordh. 1936) R. Tx. 1937, alt. 158 m | 12.06.2011 | N | (Kuziarin, 2012);  POWO: ***T. alpinum*** |
| 34 | ***Trichophorum cespitosum* (L.) Hartm.** | Transcarpathian region, near Khust | - | 24.05.1861 | N | (Danylyk et al., 2007);  POWO: ***T. cespitosum*** |
| ***Hydrocharitaceae* Juss.** | | | | | | |
| 35 | ***Caulinia graminea* (Delile) Tzvelev** | Crimea, Krasnoperekopsk district, near Ishun’ settlement | in rice paddies | summer 2006 | A | (Yena, 2008);  POWO: ***Najas graminea* Delile** |
| 36 | ***Egeria densa* Planch.** | Crimea, near Sevastopol, natural boundary “Maksymova Dacha” | in ponds | 07.2001 | A | (Byalt & Orlova, 2003);  POWO: ***Elodea densa* (Planch.) Casp.** |
| 37 | ***Elodea nuttallii* (Planch.) St. John** | Kyiv region: 1) near Pereyaslav-Khmelnytskyi, Kaniv reservoir (Kuryache Horlo tract);  2) Pereyaslav-Khmelnytskyi district, near Tsybli village, the Dnieper River | 1) and 2) in the bay | 1) 17.06.2004;  2) 05.06.2005 | A | (Chorna et al., 2006);  POWO: ***E. nuttallii*** |
| ***Juncaceae* Juss.** | | | | | | |
| 38 | ***Juncus dichotomus* Elliott** | Zhytomyr region: 1) Olevsk disrtrict, Zhubrovychi villiage; 2) Baranivka district, Zeremlia villiage, in Zeremlianske Forestry (state enterprise « Baranivka Hunting Forestry»);  3) Volyn Region, Liubeshiv district, Velyka Hlusha villiage | 1) and 3) -  2) in the forest cutting in the association *Carpinieto-Quercetum Coryloso-Caricetum (brizoides)* | 1) 15.08.2007;  2) 31.07.2008;  3) 08.08.2013 | A | (Olshanskyi & Orlov, 2013);  POWO: ***J. dichotomus*** |
| ***Iridaceae* Juss.** | | | | | | |
| 39 | ***Crocus danubensis* Kernd., Pasche, Randjel. & V.**°**Randjel.** | - | - | - | N | (Harpke et al., 2014);  POWO: ***C. danubensis*** |
| 40 | ***Iris foetidissima* L.** | Crimea: 1) Yalta vacinity, Nyzhnia Oreanda, 44.46412°N, 34.146639°E;  2) Foros, Foroskyi Park;  3) Miskhor, Miskhorskyi park;  4) surroundings of Nikita, 44°30'56"N, 34°14'03"E  5) Magarach, 44°30'31"N, 34°13'06"E | 1) forest;  2) - ;  3) near stream;  4) 230 m, disturbed ashoak forest;  5) 125 m, disturbed ash-oak forest | 1) 05.09.2003;  2) 30.12.2012;  3) 07.11.2020;  4) 07.06.2023, 11.07.2023;  5) 25.07.2023 | A | The cases of the species’ naturalization (Raab-Straube & Raus, 2024);  POWO: ***I. foetidissima*** |
| ***Liliaceae* Juss.** | | | | | | |
| 41 | ***Gagea aipetriensis* Levichev** | Crimea, near the top of the Ai-Petri yaila | yaila | 13.04.1989 | N | first described for science (Levichev, 1998);  POWO: ***G. aipetriensis*** |
| 42 | ***Gagea microfistulosa* Levichev** | Crimea, yaila near the top of the Ai-Petri | on the slope of the karst funnel | 31.04.1989 | N | first described for science (Levichev, 2008). Now it is accepted as a synonym to *G. polidorii* J.-M. Tison (Peterson et al., 2009);  POWO: ***G. × polidorii*** |
|  | *Tulipa sylvestris* L. | Rivne region, Dubrovytsia district, on the western outskirts of Hrani village, blok #27 of the Tryputnianske Forestry | in the ancient park | 2012 | A | (Melnyk et al., 2013);  POWO: *T. sylvestris*;  It cannot be accepted as a new species for the country, because early known species (*T. biebersteiniana* Schult. & Schult.f., *T. graniticola* (Klokov & Zoz) Klokov, *T. hypanica* Klokov & Zoz, *T. ophiophylla* Klokov & Zoz, *T. quercetorum* Klokov & Zoz, *T. scythica* Klokov & Zoz) consider as synonyms to *T. sylvestris* subsp. *australis* in POWO. |
| ***Nymphaeaceae* Salisb.** | | | | | | |
| 43 | ***Nuphar pumila* (Timm.) DC.** | Zhytomyr region: 1) Olevsk district, near Maidan-Kopyschenskyi village, the Ubort river;  2) Ovruch district, near Selezivka village, the Ubort river; | river: *Nupharetum pumilii* Oberd. 1953, *Nymphaeion* Oberd. 1953, *Potametalia* Koch 1926, *Potametea* Klika 1941 | 1) 01.08.2004;  2) 01.07.2004; | N | (M. Didukh et al., 2010)  POWO: ***Nuphar pumila*** |
| ***Orchidaceae* Juss.** | | | | | | |
| 44 | ***Anacamptis morio* (L.) R.M. Bateman, Pridgeon & M.W. Chase subsp. *caucasica* (K. Koch) H. Kretzschmar, Eccarius & H. Dietr.** | Crimea, NE from Alushta | grazed meadow | 18.05.2011 | N | (Hahn, 2012);  POWO: ***A. morio* subsp. *caucasica*** |
| 45 | ***Anacamptis ×simorrensis* (E.G. Camus) H. Kretzschmar, Eccarius & H. Dietr. nothosubsp. *ticinensis* (Gsell) Fateryga & Kreutz** (= *A. coriophora* (L.) R.M. Bateman, Pridgeon & M.W. Chase subsp. *coriophora* × *A. pyramidalis* (L.) Rich.) | Crimea, Baydary Valley, vicinity of Kyzylove village | - | 03.06.2010 | N | New taxonomical combination, but this hybrid has never noted for Ukraine (A. V. Fateryga & Kreutz, 2014);  POWO: ***A. × simorrensis*** |
| 46 | **×*Dactylocamptis uechtritziana* (Hausskn.) M. Peregrym et Kuzemko** (= *Dactylorhiza incarnata* (L.) Soó × *Anacamptis palustris* (Jacq.) R.M. Bateman, Pridgeon et M.W. Chase) | Kharkiv region, Zmiiv district, near Tymchenky village, in the floodplain of the left bank of the Mzha River, 49,7465°N, 36,1962°E | meadows: *Festucetum pratensis* Soó 1938, *Festucion pratensis* Sipaylova, Mirkin, Shelyag & V. Solomakha 1985, *Arrhenatheretalia* Pawł. 1928, *Molinio-Arrhenatheretea* R. Tx. 1937. | 06.2009 | N | (M. Peregrym & Kuzemko, 2010);  POWO: **×*D. uechtritziana*** |
| 47 | ***×Dactylocamptis uechtritziana* (Hausskn.) B. Bock ex M. Peregrym & Kuzemko nothosubsp. *magyarii* (Soó) Fateryga & Kreutz** (= *Anacamptis palustris* (Jacq.) R.M. Bateman, Pridgeon & M.W. Chase subsp. *elegans* (Heuff.) R.M. Bateman, Pridgeon & M.W. Chase × *Dactylorhiza incarnata* (L.) Soó) | Crimea, Baydary Valley, vicinity of Tylove village | - | 10.06.2010 | N | New nothosubspecies and taxonomical combination (A. V. Fateryga & Kreutz, 2014);  POWO: ***×Dactylocamptis uechtritziana* nothosubsp. *magyarii*** |
| 48 | ***Epipactis albensis* Nováková et Rydlo** | In Prots (2009) [but see Ljubka et al. (2014)]:  Transcarpathian region:  1) [Berehove district], 5 localities at the flood of the Borzhava River between villages Velyki Berehy, Shalanky and Kvasovo  2) [Uzhhorod district], 3 localities at the flood of the Latorytsya River near villages Mali Heivtsi and Chomonyn,  3) [Uzhhorod district] at the flood of the Uzh River, at the borders of Uzhhorod;  In Ljubka et al. (2014):  Transcarpathian region, Berehove district:  4) in vicinity of Chetfalva, N 48.12154°, E 22.78957°;  5) Fanchykovo, N 48.08708°, E 22.91501°;  6) Drotyntsi, N 48.09599°, E 22.98899° | 1) – 3) in low-lying floodplain oak-ash forests at the boundaries of 100–140 m a.s.l.: *Fraxino pannonicae-Ulmetum*, *Carici remotae-Fraxinetum*, *Fraxino pannonicae-Carpinetum*, less often – *Fraxino angustifoliae-Alnetum glutinosae* (*Alnion incanae, Querco-Fagetea*) and Salici-Populetum (*Salicetea purpureae*);  4) Poplar plantation  5) Riverine willow-poplar woodland  6) Riverine willow-poplar woodland | 1) – 3) 1997 [– 2009];  4) – 6) 2012 | N | (Prots, 2009). Ljubka et al. (Ljubka et al., 2014) claim that the occurrence of *E. albensis* in Ukraine has been remained unproven until their publication, because the published photograph illustrates *E. helleborine* (L.) Crantz in the mentioned study above, as well as there are not any references to Ukrainian publications and herbaria. According to them, in August 2012 three populations of *E. albensis* were found in the Transcarpathian Lowland;  POWO: ***E. albensis*** |
| 49 | ***Epipactis condensata* Boisser ex Young** | Crimea: 1) Mt Karadag;  2) Alupka;  3) Zelenohirya, riv. Arpat  4) Laspi | - | 1) 25.6.1926;  2) 6.1876;  3) 2.6. 1990;  4) 22.6.[year - unknown] | N | (Efimov, 2008). The presence of *E. condensata* in the Crimea is questionable: most specimens previously identified as *E. condensata* were in fact *E. krymmontana*, so the only remaining historic basis for the presence of *E. condensata* in the Crimea is the collection from Mt Karadag; however, the recent collection from Mt Lapata in the Crimea might also be this species (A. V. Fateryga et al., 2014);  POWO: ***E. condensata*** |
| 50 | ***Epipactis condensata* Boiss. ex D.P. Young subsp. *condensata*** | Crimea: 1) Mt Karadag;  2) Yalta Mountain Forest Reserve, Mt. Lapata | - | 1) 25.06.1926  2) 27.07.2013 | N | (A. V. Fateryga et al., 2014);  POWO: this species is accepted, but this subspecies is not mentioned |
| 51 | ***Epipactis helleborine* subsp. *levantina* Kreutz, Óvári & Shifman** | Crimea, near Orlyne | In a forest | 30.05.2012 | N | (Hahn, 2012);  POWO: ***E. helleborine* subsp. *tremolsii* (Pau) E.Klein** |
| 52 | ***Epipactis helleborine* (L.) Crantz subsp. *orbicularis* (C. Richt) E. Klein** | Crimea, Yalta district, Lopata Mt. | pine forest | 13.07.2005 | N | (Kreutz & Fateryga, 2012);  POWO: ***E. helleborine* subsp. *helleborine*** |
| 53 | ***Epipactis krymmontana* Kreutz, Fateryga & Efimov** | Crimea:1) Bilohirsk district, Zemlianychne [Ortalan]  2) Alupka;  3) Bakhchysarai district, near Kokkoz [Sokolyne];  4) Bakhchysarai district, Chaanbair riv. [Kokkozka basin, near Sokolyne];  5) vicinity of Yalta, near waterfall Uchansu;  6) vicinity of Yalta, Miskhor;  7) Staryi Krym;  8) vicinity of Yalta, Koreiz;  9) E Baidary Gates [pass through Mt Baidarska Yaila];  10) Bilohirsk district, Zemlianychne [Ortalan];  11) Bakhchysarai district, near Sokolyne;  12) Simferopol district, near Perevalne | Beech forests, often mixed with oak and hornbeam, on calcareous soils, 350–1250 m | 1) 27.06.2012;  2) VI.1876;  3) 22.07.1897;  4) 22.07.1897;  5) 24.07.1909;  6) 20.06.1914;  7) 27.06.1915;  8) 13.06.1951;  9) 02.06.1981;  10) 23.06.2012;  23.06.2013;  11) 29.06.2013;  12) 05.07.2013 | N | first described for science (A. V. Fateryga et al., 2014);  POWO: ***E. krymmontana*** |
| 54 | ***Epipactis leptochila* (Godfery) Godfery** | Crimea: 1) Sevastopol, near Kyzylove, 44°24′45′′N, 33°46′21′′E;  2) Baydary valley, near Rodnikovske village, Bossa River, 44°27′14′′N, 33°50′41′′E;  3) Bakhchysarai district, Aidymytryi tract, 44°30′22′′N, 33°54′41′′E, and 44°30′21′′N, 33°54′35′′E | in shady beech-hornbeam-ash, oak, oak-hornbeam, and oak-dogwood forests at an altitude of 350–700 m above sea level | 1) 01.07.2015;  2) 16.07.2015;  3) 17.07.2015, 20.07.2015 | N | (V. V. Fateryga et al., 2015);  POWO: ***E. leptochila*** |
| 55 | ***Epipactis muelleri* Godfery** | Crimea: 1) Dolhorukivska Yaila Mt., 44°50´20´´N, 34°21´37´´E and 44°50´25´´N, 34°21´54´´E;  2) Babuhan Yaila Mt., 44°35´14´´N, 34°16´50´´E | a) west slope of, oak forest, 750 m above sea level, as well as beech forest, 840 m above sea level;  b) south slope, pine forest, 850 m above sea level | 1) 05.07.2013;  2) 07.07.2013 | N | (V. V. Fateryga et al., 2013);  POWO: ***E. muelleri*** |
| 56 | ***Epipactis persica* (Soó) Hausskn. ex Nannf. subsp. *taurica* (Fateryga & Kreutz) Fateryga & Kreutz** | Crimea: 1) Yalta district, Yalta Mountain-Forest Nature Reserve, slopes of Lopata Mountain;  2) Simferopol district, vicinity of Ayan Reservoir | 1) open, well-illuminated two-layer pine forest with monodomination of *Pinus nigra* J.F. Arnold var. *pallasiana* (Lamb.) Holmboe;  2) meadow with xerothermal vegetation on a western slope | 1) 09.07.2011;  2) 28.06.2005 | N | A new combination (A. V. Fateryga & Kreutz, 2014), but these plants were first described for science as *E. taurica* Fateryga & Kreutz (A. V. Fateryga & Kreutz, 2012). However, *E. persica* was noted by Yena (2012) the first time for Ukraine, but he specified that data about this finding is in press, and it is absent still. We guess that mentioned later *E. persica* subsp. *persica* by Fateryga and Kreutz without any specification (A. V. Fateryga & Kreutz, 2014) may be concerned to this case;  POWO: ***E. persica*** |
| 57 | ***Epipactis turcica* Kreutz** | Crimea: 1) Yalta district, Nikita, «Mys Martyan» Reserve;  2) Sarych Cape | formations of *Juniperus excelsa* M. Bieb. and *Quercus pubescens* Willd. | 1) 09.06.2011;  2) 08.06.2009 | N | (Kreutz & Fateryga, 2012);  POWO: ***E. helleborine* subsp. *tremolsii* (Pau) E.Klein** |
| 58 | ***Neotinea × dietrichiana* (Bogenh.) H. Kretzschmar, Eccarius & H. Dietr.** (= *N. tridentata* (Scop.) R.M. Bateman, Pridgeon & M.W. Chase × *N. ustulata* (L.) R.M. Bateman, Pridgeon & M.W. Chase) | Crimea, Mt. Tyrke Yaila. | - | 11.06.2012 | N | (A. V. Fateryga & Kreutz, 2014);  POWO: ***N. × dietrichiana*** |
| 59 | ***Ophrys × aghemanii* Renz** (*O. mammosa* Desf. subsp. *mammosa* × *O. oestrifera* M. Bieb.) | Crimea, vicinity of Zelenohirya, Pananyan-Uzen River valley, 44°53′01′′N, 34°42′31′′E | ca. 390 m. a. s. l., rocky slope to river, among shrubs | 28.05.2020 | N | (V. V. Fateryga et al., 2022);  POWO: ***O. × aghemanii*** |
| 60 | ***Orchis × beyrichii* Kern. nothosubsp. *mackaensis* (Kreutz) Fateryga & Kreutz** (*O. militaris* L. subsp. *stevenii* (Rchb. f.) B. Baumann, H. Baumann, R. Lorenz & Ruedi Peter × *O. simia* Lam.) | Crimea, in the vicinity of Perevalne village, Ayan Natural Landmark | - | 19.5.2011 | N | This hybrid was listed before as *O. militaris* × *O. simia*, but here is its new nothosubspecies and taxonomical combination (A. V. Fateryga & Kreutz, 2014);  POWO**: *O. × beyrichii* nothosubsp. *golestanica* (Renz) H.Kretzschmar, Eccarius & H.Dietr.** |
| 61 | ***Orchis militaris* L. subsp. *stevenii* (Rchb.f.) B. Baumann, H. Baumann, R. Lorenz & Ruedi Peter** | Crimea, all know location of *O. militaris* | - | - | N | It is established that *O. militaris* is only represented by this subspecies in Crimea (A. V. Fateryga & Kreutz, 2014);  POWO: ***O. militaris* subsp. *stevenii*** |
| 62 | ***Orchis × penzigiana* A. Camus** (*O. mascula* (L.) L. × *O. provincialis* Balb.ex Lam. & DC.) | Crimea, near Simeiz in vicinity of Yalta. | - | 30.04.2013 | N | (A. V. Fateryga & Kreutz, 2014);  POWO: ***O. × penzigiana*** |
| 63 | ***Platanthera × hybrida* Brügger** (= *P. bifolia* (L.) Rich. × *P. chlorantha* (Custer) Rchb.). | 1) Crimea, very usual hybrid at numerous localities, like an example: Simferopol district, vicinity of Perevalne village, Kurlukbash Natural Landmark;  2) Odesa region, Tatarbunary district, near Lebedivka village, National Nature Park “Tuzlivski Lymany” | 1) oak forest;  2) artificial forest plantations of *Quercus robur* | 1) 17.06.2011;  2) 05.2015 | N | 1) (A. V. Fateryga & Kreutz, 2014);  2) (Popova, 2015);  POWO: ***P. × hybrida*** |
| ***Poaceae* Barnhart** | | | | | | |
| 64 | ***Aegilops peregrina* (Hack.) Maire & Weiller** | Crimea: 1) Sevastopol district, Balaklava vicinity, Vitmerova balka (Vitmer’s ravine), 44°29'55.64"N, 33°37'45.45"E;  2) Varnautska valley, Honcharne vicinity, 44°28'13"N, 33°42'08"E | 1) 260  m, conglomerate hill, dry grasslands on the edge of woodlands of *Juniperus excelsa* M. Bieb. and *Quercus pubescens* Willd.;  2) 270 m, dry grassland on abandoned farmland | 1) 16.05.2017, 10.06.2019;  2) 26.05.2017 | A | (Raab-Straube & Raus, 2020, p. 12);  POWO: ***A. peregrina*** |
| 65 | **× *Agrotrigia hajastanica* (Tzvelev) Tzvelev** (= *Agropyron cristatum* (L.) Gaertn. s.l. × *Elytrigia repens* (L.) Desv. ex Nevski) | Crimea, Sevastopol: 1) S corner of Omega Bay, 44°35’45”N, 33°26’55”E;  2) S side of Komyshova Bay, 44°34’20”N, 33°25’30”E, near entrance to ferry terminal | 1) stony seashore, elev. 0 m;  3) parking area for trucks, elev. 10 m | 1) 29.07.2014;  2) 31.07.2014 | N | (Seregin, Yevseyenkov, et al., 2015);  POWO: **× *Agrotrigia hajastanica*** (an unplaced name) |
| 66 | ***Anthoxanthum aristatum* Boiss.** | Chernivtsi city, the railway station, 48.30095ºN, 25.93103ºE | between the platform and the railway track, alt. 168 m, | 24.06.2020 | A | (Moysiyenko et al., 2023)  POWO: ***A. aristatum*** |
| 67 | ***Avena clauda* Durieu** | Crimea: 1) near Hurzuf, 44°33′N, 34°17′E;  2) Sevastopol; Soliona Bay and Mayachnyi Peninsula, 44°34′N, 33°24′E | - | 1) 1996;  2) 2011-2012 | N (?) | (Ryff et al., 2013);  POWO: ***A. clauda*** |
|  | *Brachypodium pinnatum* (L.) P. Beauv. subsp. *juzepczukii* Tzvelev | Crimea, in the reserve near the Chuchel road | in a pine forest | 23.07.1948 | N | first described for science (Tzvelev, 2006), but it considers in POWO only as *B. pinnatum*, which is already know in the country (Mosyakin & Fedoronchuk, 1999) |
| 68 | ***Brachypodium sylvaticum* (Huds.) P.Beauv. subsp. *spryginii* Tzvelev** | Crimea: 1) Alupka – Ai-Petri;  2) Ai-Petri, Yailin highway barracks | 1) yaila;  2) - | 1) 08.07.1914;  2) 03.08.1922 | N | first described for science (Tzvelev, 2006), but it considers in POWO as ***B. s.* subsp. *sylvaticum***. However, this subspecies was known in the flora of Ukraine before, but has not been mentioned by Mosyakin and Fedoronchuk (1999) |
|  | *Bromopsis inermis* (Leyss.) Holub subsp. *aristata* (Schur) Tzvelev | Chernivtsi region, Putyla district, Konyatyn village, the valley of the Bilyi Cheremosh River | - | 13.07.1961 | N | first described for science (Tzvelev, 2006), but it considers in POWO only as *B. inermis,* which is already know in the country (Mosyakin & Fedoronchuk, 1999). Also, two other subspecies of this species were reported for the flora of Ukraine (*B. i.* subsp. *inermis* and *B. i.* subsp. *australis* (Zherebina) Soskov et Sinjakov) early, but the first one is considering as *B. inermis* too, the second one – not mentioned in POWO. |
| 69 | ***Calamagrostis purpurea* (Trin.) Trin.** | Ivano-Frankivsk Region, Chornohora Mts, peat bog Tsybulnyk between Mt. Breskul and Mt. Pozhyzhevska, 48°09′15.4′′N, 24°31′48.1′′E | peat bog, 1375 m a.s.l. | 28.07.2020 | N | (Kobiv et al., 2022);  POWO: ***C. purpurea*** |
| 70 | ***Diplachne fasticularis* (Lam.) P. Beauv.** | Odesa region, Izmail district, near Vylkove town, Danube Biosphere Reserve, Nova Zemlia Spit | alluvial areas | 02.10.2002 | A | (Dubyna et al., 2003);  POWO: ***D. fusca* (L.) P. Beauv. ex Roem. & Schult. subsp. *fascicularis* (Lam.) P.M.Peterson & N.Snow** |
| 71 | ***Elytrigia atherica* (Link) Kerguélen** | Crimea, Sevastopol: 1) Kruhla Bay (Omega Bay), 44°35'43"N, 33°26'54"E;  2) Komyshova Bay, 44°34'23.34"N, 33°25'34.82"E, 44°34'23"N, 33°25'39"E;  3) Kozacha Bay, 44°34'08"N, 33°24'49"E;  4) Striletska Bay, 44°35'39"N, 33°28'06"E | 1) 1 m, sea shore;  2) 1 m, sea shore;  3) 1 m, sea shore;  4) 2–3 m, sea coast | 1) 2.08.2014, 13.06.2018, 19.07.2020;  2) 26.03.2020, 19.07.2020;  3) 19.07.2020;  4) 19.07.2020 | N | (Raab-Straube & Raus, 2024);  POWO: ***Elymus athericus* (Link) Kerguélen** |
| 72 | ***Elytrigia striatula* (Runemark) Holub** | 1) Kherson region, Hola Prystan district, the Tendra island;  2) Mykolaiv region, Ochakiv district, coast of the Kinburn Peninsula from the side of the Yagorlytski Bay, west to the village Pokrovka | 1) and 2) marine sand and shell sediments within the coastal bar | a) 30.06.1997, 16.07.1999;  b) 10.08.1999 | N | (Umanets, 2000b);  POWO: ***Thinopyrum junceum* (L.) Á.Löve** |
| 73 | ***Eragrostis virescens* J. Presl** | Crimea, Yalta vicinity, Nikita, Nikitsky Botanical Garden, Upper Park, 44°30'41''N, 34°13'56''E | 150 m, in flower bed under regular watering with planting of chrysanthemums | 19.07.2021 | A | (Raab-Straube & Raus, 2021b);  POWO: ***E. virescens*** |
| 74 | ***Festuca galiciensis* Bednarska** | Ivano-Frankivsk region, Halych district: 1) 1.5 km to E from the Kuropatnyky village: 49°17’03”N, 24°40’12”E;  2) between village Ozeryany and v. Kuropatnyky;  3) v. Kuropatnyky, 49°17’1.98”N; 24°40’8.87”E;  4) v. Ozeryany | 1) . steppe rock in gypsum on the south-western slope;  2) hill outcrops of gypsum strata with semi-arid steppe and petrophilic vegetation;  3) rock-steppe on the gypsum outcrops;  4) pasture meadow near gypsum rocks | 1) 30.05.2010;  2) 12.09.2005; 20.05.2006;  3) 30.05.2010, 02.07.2011, 08.06.2012, 09.06.2013, 19.07.2014;  4) 09.06.2013 | N | first described for science (Bednarska & Brazauskas, 2017);  POWO: ***F. galiciensis*** |
| 75 | ***Festuca* × *polovina* Bednarska** (=*F. polesica* Zapał. × *F. ovina* L.) | Western Polissya | on sandy substrate in sparse pine forests, on forest edges, often in transformed areas | - | N | first described for science (Bednarska, 2009);  POWO: ***F.*× *polovina*** (an unplaced name) |
| 76 | ***Lolium × elongatum* (Ehrh.) Banfi, Galasso, Foggi, Kopecký & Ardenghi** (= *L. perenne* L. × *L. pratense* (Huds.) Darbysh.) | Kyiv, M.M. Gryshko National Botanical Garden of the National Academy of Sciences of Ukraine | - | 25.11.2020, 6.09.2021 | A | first cases of escape from cultivation (Shynder, Doiko, et al., 2022);  POWO: ***L. × elongatum*** |
| 77 | ***Lolium* × *holmbergii* (Dörfl.) Banfi, Galasso, Foggi, Kopecký & Ardenghi** (= *L. arundinaceum* (Schreb.) Darbysh. × *L. perenne*) | Kyiv, Holosiivskyi district, near the path, behind Feofania Park, 50.346980ºN, 30.494778ºE | on the meadows | 07.04.2020 | N | (Shynder et al., 2024);  POWO: ***L.*× *holmbergii*** |
| 78 | ***Melica smirnovii* Tzvelev.** | west of the country including Prykarpattia | ? | - | N | first described for science (Tzvelev, 2012);  POWO: ***M. smirnovi*** |
| 79 | ***Poa egorovae* Tzvelev** | Transcarpathian region: 1) Svydovets Mts., Hereshaska Mt.;  2) Tiachiv district, 1.5 km above Ruska Mokra village, the gorge of the Yakovets River;  3) Svydovets Mts., Blyznytsia Mt., 18-22 km to SW from Yasynya village | 1) limestone slopes, alt. 1800 m;  2) -;  3) mountain meadows | a) 07.1964;  b) 15.09.1949;  c) 29.07.1975 | N | first described for science (Tzvelev, 2009);  POWO: ***P. egorovae*** |
| 80 | ***Poa klokovii* Tzvelev** | Kherson province, floodplains of the Dniester near Yasky village (now – Odesa region, Odesa district) | - | 23.05.1905 | N | first described for science (Tzvelev, 2009);  POWO: ***P. klokovii*** |
| 81 | ***Poa paczoskii* Tzvelev** | Kherson region, Beryslav district, near Tiahynka village, along the Tiahynka River | along the high bank | 06.06.1910 | N | first described for science (Tzvelev, 2009);  POWO: ***P. paczoskii*** |
| 82 | ***Polypogon maritimus* Willd.** | Crimea, Sevastopol area, near Balaklava, 11^th^ km of the highway | ? | 12.07.2017 | N | (Bondareva et al., 2018);  POWO: ***P. maritimus*** |
| 83 | ***Sesleria caerulea* (L.) Ard.** | Lviv region, Zolochiv district, near Zarvanytsya village, the valley of the Zolochivka River | carbonate marshes and peat meadows | 08.1990 | N | (Kuziarin, 2003);  POWO: ***S. caerulea*** |
| 84 | ***Sporobolus cryptandrus* (Torr.) A. Gray** | Luhansk region, Slovyanoserbsk (now- Shchastia) district, near Triokhizbenka village, the branch “Triokhizbenskyi Steppe” of the Luhansk Nature Reserve | loosely packed areas of sand, as part of communities with the participation of *Artemisia abrotanum* L., *Calamagrostis epigeios* (L.) Roth, *Koeleria sabuletorum* (Domin) Klokov | 2010 | A | (Gouz & Timoshenkova, 2017);  POWO: ***S. cryptandrus*** |
| 85 | ***Vulpia octoflora* (Walt.) Rydb.** | Luhansk region, Stanytsia Luhanska district, between Tranzytna and Kondrashivka-Nova railway stations | along the railway | 24.04.2001 | A | (Sova & Mosyakin, 2002);  POWO: ***Festuca octoflora* Walter** |
| ***Potamogetonaceae* Bercht. & J.Presl** | | | | | | |
| 86 | ***Althenia orientalis* (Tzvelev) Garcia-Mur. et Talavera** | Crimea, Lenine district: 1) northeastern edge of the Shcholkine city, northern separated reservoir of the Aktash lake system, 45°26’02.4”N, 35°49’48.6”E;  2) 4.6 km to E from Kalynivka village, a small reservoir (50 × 100 m) to S from the Aktash lake, 45°20’03.6”N, 35°48’16.4”E | 1) salt water, at a depth of 15-20 cm;  2) salt water | 1) and 2) 10.08.2015; | N | (Kipriyanova & Shadrin, 2017);  POWO: ***A. orientalis*** |
| 87 | ***Groenlandia densa* (L.) Fourr.** | Lviv region, Boryslav, pound | in shallow water | 08.2007 | N | (Borsukevych, 2010);  POWO: ***G. densa*** |
| 88 | ***Potamogeton* × *angustifolius* J. Presl** (*P. gramineus* L. × *P. lucens* L.) | Crimea, Baydarska valley, pond to N from Novobobrovske, 44°30’15”N, 33°51’00”E | pond, in water, elev. 330 m | 02.08.2014 | N | (Seregin, Yevseyenkov, et al., 2015);  POWO: ***P.*× *angustifolius*** |
| 89 | ***Potamogeton filiformis* Pers.** | Kherson region, Henichesk district, between Semykhatka and Chervonopraporne villages, Syvash Lake | a shallow water area | 05.07.2000 | N | (Pavlov, 2001);  POWO: ***Stuckenia filiformis* (Pers.) Börner** |
| ***Ruppiaceae* Horan.** | | | | | | |
| 90 | ***Ruppia drepanensis* Tineo** | Crimea, Dzhankoi district, Toganash village (now – Solone Ozero) | In a salt lake | 17.08.1886 | N | (Kipriyanova & Shadrin, 2017);  POWO: ***R. drepanensis*** |
| ***Typhaceae* Juss.** | | | | | | |
| 91 | ***Typha austro-orientalis* Mavrodiev** | Crimea, Vicinity of Liubymivka | - | 26.07.2014 | N | (Seregin, Yevseyenkov, et al., 2015);  POWO: ***T. austro-orientalis*** |
| **EUDICOTS** | | | | | | |
| ***Aizoaceae* Martinov** | | | | | | |
| 92 | ***Mesembryanthemum ×vascosilvae* (Gideon F.Sm., E. Laguna, F. Verloove & P.P. Ferrer) Sáez & Aymerich** (= *M. cordifolium* L. f. × *M. haeckelianum* A.Berger) | Kyiv, at the margin of the Syrets (Syretskyi) Park | in and near an abandoned or neglected flower bed | 14.10.2019 | A | first cases of escape from cultivation (Mosyakin & Mosyakin, 2021);  POWO: no data |
| ***Amaranthaceae* Juss.** | | | | | | |
| 93 | ***Axyris amaranthoides* L.** | Sumy region, Seredena-Buda district: a) Ochkine village, about 30 m from a pig farm;  b) Novovasylivka village | a) near the fence of the homestead;  b) in the center of the village | a) 27.07.2000;  b) 10.08.2002 | A | (Panchenko & Mosyakin, 2005);  POWO: ***A. amaranthoides*** |
| 94 | ***Amaranthus viridis* L.** | Kherson, Suvorovskyi district, Spartakivskyi lane (two blocks north of the central market) | a weedy front garden on the street | 28.08.2000 | A | (Moysienko & Mosyakin, 2008);  POWO: ***A. viridis*** |
| 95 | ***Chenopodium ucrainicum* Mosyakin & Mandák** | 1) Kyiv, Shevchenkivskyi District, Tatarka historical neighborhood, Baggovutivska Street, near (behind) the Promenada Mall, 50.470357 N, 30.479655 E;  2) city of Kyiv (two sites);  3) Kyiv Region, Vasylkiv District, Hrebinky (occurring in several localities in the area);  4) Rivne region, Ostrog | 1) shady areas under trees on slopes with ruderal vegetation;  2), 3), 4) - | 1) 02.10.2019;  2), 3) - ;  4) 14.07.2003 | N (?) | first described for science (Mosyakin & Mandák, 2020);  POWO: no data |
| 96 | ***Dysphania ambrosioides* (L.) Mosyakin & Clemants** | Kyiv, M.M. Gryshko National Botanical Garden of the National Academy of Sciences of Ukraine, in and near the New Crops Lab area, N 50.417194, E 30.557558 | - | 2019, 2020 | A | first cases of escape from cultivation (Mosyakin & Mosyakin, 2021);  POWO: ***D. ambrosioides*** |
| 97 | ***Salsola paulsenii* Litv.** | Kyiv, in the northwestern part of the city, Obolon, near the grain elevator and brewery facilities: N 50° 30' 34.1879", E 30° 28' 47.0765" | along railway tracks | 18.09.2017 | A | (Mosyakin, 2017);  POWO: ***S.paulsenii*** |
| ***Apiaceae* Lindl.** | | | | | | |
| 98 | ***Bifora testiculata* (L.) Spreng.** | Crimea, eastern outskirts of Hurzuf, the tract "Mertva Dolyna" | herbaceous-semi-shrub vegetation of the Mediterranean type | 1990, 2004 | N (?) | (Yena et al., 2006);  POWO: ***B. testiculata*** |
| 99 | ***Bupleurum veronense* Turra** | Crimea, Sevastopol: 1) below roman citadel on Mt Vysota Gorna;  2) vicinity of Maximova Dacha estate, 44°33’16”N, 33°32’26”E;  3) between 5^th^ km of Balaklava highway and Maximova Dacha estate, 44°33’10”N, 33°32’30”E;  4) N edge of Maximova Dacha estate, 44°33’45”N, 33°32’25”E, upper part of Maximova Balka valley | 1) -  2) *Cercis* cultures;  3) steppe plot on plateau, elev. 160 m;  4) steppe slope, elev. 130 m | 1) 18.06.2013;  2) 08.06.2014  3) 28.07.2014;  4) 28.07.2014 | A (?) | (Seregin, Yevseyenkov, et al., 2015);  POWO: ***B. veronense*** |
| 100 | ***Cyclospermum leptophyllum* (Pers.) Sprague ex Britton et P. Wilson** | Crimea, Yalta, Nikita Botanical Garden, arboretum | in cracks between paving slabs and near curbs on park paths | summer 2009 | A | probably, this species escaped from cultivation in the garden, but there is no evidence (Ryff, 2011);  POWO: ***C. leptophyllum*** |
| 101 | ***Daucus guttatus* Sm.** | Crimea: 1) Chornomorskyi district, 3 km WNW of Olenivka, 45°23'30''N, 32°29'50''E  2) Sevastopol, Balaklava, 44°31'58''N, 33°36'58''E;  3) Sevastopol, S of Orlivka, 44°43'09.3''N, 33°34'45.2''E;  4) Sevastopol, Chornorichcha vicinity, 44°32'35.6''N, 33°39'46.1''E;  5) Saky district, Novoozerne, 45°23'49.9''N, 33°08'12.0''E;  6) Sevastopol, N slope of Mount Sapun, E of Khomutov ravine, 44°33'22.8''N, 33°33'42.5''E;  7) Sevastopol, near Generala Zhidilova str., 44°35'32.0''N, 33°35'24.4''E | 1) 20 m, steppe with *Artemisia santonica* Lam., *Festuca valesiaca* Gaudin and *Stipa capillata* L. on rocky ground;  2) 95 m, dry grassland;  3) 60 m, dry grassland;  4) 45 m, dry grassland;  5) 100 m, steppe;  6) 170 m, disturbed dry grassland habitat on side of road,  7) 165 m, dry grassland | 1) 14.08.2008;  2) 4.07.2018, 23.07.2018;  3) 20.06.2020;  4) 27.06.2020;  5) 4.07.2020;  6) 19.07.2020;  7) 8.07.2021 | N | (Raab-Straube & Raus, 2023);  POWO: ***D. guttatus*** |
| 102 | ***Pimpinella tragium* Vill.** | Crimea: near Sevastopol, Foros, Sudak and Feodosia, the Ak-Kaya Mount near Bilohirsk | on limestone, clay and shale outcrops | - | N | (Tzvelev, 2004);  POWO: ***P. tragium*** |
| 103 | ***Torilis pseudonodosa* Bianca** | Kherson region, Skadovsk district, near the village of Zaliznyi Port, “Potiivska” section of the Black Sea Biosphere Reserve, N 46.133507°; E 32.229562° | a saline steppe, close to the Black Sea coast, -2 m a.s.l. | 28.05.2021 | N | (Moysiyenko et al., 2021);  POWO: ***T. pseudonodosa*** |
| ***Apocynaceae* Juss.** | | | | | | |
| 104 | ***Apocynum cannabinum* L.** | Kyiv, M.M. Gryshko National Botanical Garden of the National Academy of Sciences of Ukraine | - | - | A | first cases of escape from cultivation (Shynder, 2019);  POWO: ***A. cannabinum*** |
| 105 | ***Nerium oleander* L.** | Crimea: 1) Sevastopol, Laspi bay, 44°23'47"N, 34°43'29"E;  2) Yalta vicinity, Hurzuf, Pushkin embankment, 44°32'12"N, 34°16'25"E;  3) Yalta vicinity, Nikitsky Botanical Garden, Lower Park, Palm Alley, 44°30'32"N, 34°14'02"E | 1) 5 m, boulder-block beach, single plant in generative stage;  2) 8 m, in asphalt cracks near a fence and wall;  3) 100  m, irrigated flower bed | 1) 5.07.2017;  2) 16.07.2020, 02.02.2021;  3) 09.02.2021 | A | first cases of escape from cultivation (Raab-Straube & Raus, 2021a);  POWO: ***N. oleander*** |
| 106 | ***Vincetoxicum svetlanae* Ostapko** | Donetsk region, Amvrosiivka district, local reserve “The tract Prystenske”, 47°48ʹ40,3ʺN, 38°32ʹ28,5ʺE | oak forest | 25.06.2006 | N | first described for science (Ostapko, 2018);  POWO: ***V. svetlanae*** |
| ***Asteraceae* Bercht. & J. Presl** | | | | | | |
| 107 | ***Artemisia latifolia* Ledeb.** | Luhansk region, Milove district, “Striltsivskyi Step” | in the thalweg of the ravine | 1952 | N | (Shumilova, 2002);  POWO: ***A. latifolia*** |
| 108 | ***Carduus acicularis* Bertol.** | Crimea, Yalta region, vicinity of the Holuba Zatoka settlement, Limeny valley, territory of Yalta Nature Reserve: 1) 44°24'19"N, 33°58'52"E;  2) recreational complex near sea coast, 44°24'07"N, 33°59'17"E | 1) 140 m, *Pinus* plantation, side of fire-fighting country road;  2) 15 m, roadside | 1) 9.06.2023;  2) 28.06.2023 | A | (Raab-Straube & Raus, 2024)  POWO: ***C. acicularis*** |
| 109 | ***Centaurea dealbata* Willd.** | Kyiv, M.M. Gryshko National Botanical Garden of the National Academy of Sciences of Ukraine | - | - | A | first cases of escape from cultivation (Shynder, 2019);  POWO: ***Psephellus dealbatus* (Willd.) K. Koch** |
| 110 | ***Eclipta prostratа* (L.) L.** | Odesa region, Izmail district, near Vylkove town, Danube Biosphere Reserve, Zhelanna Spit | alluvial areas | 28.07.1997 | A | (Dubyna et al., 2003);  POWO: ***E. prostratа*** |
| 111 | ***Erigeron bilbaoanus* (J. Rémy) Cabrera** | Crimea: 1) SW outskirts of Hurzuf, “Zhemchuzhina Kryma” recreation complex;  2) road near Korovin Holiday House;  3) seashore between Hurzuf and Ay-Danil, “Lagoon” complex under construction | Roadsides, asphalt and concrete places | 1) 01.09.2013;  2) 14.09.2013;  3) 13.10.2013 | A | (Raab-Straube & Raus, 2017, p. 8);  POWO: ***E. floribundus* (Kunth) Sch.Bip.** |
| 112 | ***Erigeron karvinskianus* DC** | Crimea, Yalta, Lomonosova Str., Uchan-Su River bed, 44°29'22"N, 34°08'30"E | 50 m, in cracks of retaining wall | 10.09.2020 | A | first cases of escape from cultivation (Raab-Straube & Raus, 2021a);  POWO: ***E. karvinskianus*** |
| 113 | ***Erigeron sumatrensis* Retz.** | Crimea: 1) Simeiz, Krasnomayakska Str.;  2) Yalta, bus station;  3) Yalta, Lomonosova Str.;  4) Dolossy, Yaltinski Mountain Forest natural reserve, disturbed area within *Pinus pallasiana* forest;  5) Nikita, Nikitsky Botanical Garden;  6) Ay-Danil (Danylivka);  7) Hurzuf, “Zhemchuzhyna Kryma” recreation complex;  8) Hurzuf, Leninhradska Str. | Streets, pavements, roadsides, asphalt and concrete areas, abandoned flowerbeds, vineyard edges, pond shores | 1) 09.12.2014;  2) 23.08.2013;  3) 24.05.2014;  4) 24.08.2013;  5) 16.09.2013;  6) 29.09.2013;  7) 01.09.2013;  8) 08.10.2017 | A | (Raab-Straube & Raus, 2017, p. 8);  POWO: ***E. sumatrensis*** |
| 114 | ***Galatella ×feketegaborii* A. Takács, Sennikov et Sramkó** (= *G. villosa* (L.) Rchb. f. × *G. linosyris* (L.) Rchb. f.) | Mykolaiv region, Antonivka, Yelanetskyi Step | a slope in a valley with steppe vegetation surrounded by arable fields | 01.09.2019 | N | first described for science (Takács et al., 2020);  POWO: - |
| 115 | ***Gelasia villosa* Cass. subsp. *columnae* (Guss.) Bartolucci, Galasso & F.Conti** | Crimea: 1) Sevastopol, Mount Vysota Horna, 44°31'22.74"N, 33°32'58.06"E; 44°31'21.05"N, 33°33'06.01"E, 44°31'22"N, 33°33'00"E;  2) Sevastopol, in the Mramorna Beam area, 44°30'27.32"N, 33°31'34.36"E | open clayey and rocky limestone slopes, class *Festuco-Brometea* Br.-Bl. et Tx ex Soó 1947, less often – in shrub communities of the *Crataego-Prunetea* Tx 1962 nom. conserv. propos. | 1) 31.05.2020, 21.06.2020, 19.07.2020;  2) 26.06.2020 | A | (Ryff et al., 2023);  POWO: ***G. villosa* Cass. subsp. *columnae*** |
| 116 | ***Helichrysum tenderiense* Umanets** | Kherson region, Hola Prystan district, the Tendra island | sandy littoral of the Black Sea | 29.06.1997 | N | first described for science (Umanets, 2000a);  POWO: ***H. tenderiense*** |
| 117 | ***Heliopsis helianthoides* (L.) Sweet subsp. *scabra* (Dunal) T.R.Fisher** | Kyiv, M.M. Gryshko National Botanical Garden of the National Academy of Sciences of Ukraine | - | - | A | first cases of escape from cultivation (Shynder, 2019);  POWO: ***H. helianthoides*** var. *scabra* (Dunal) Fernald |
| 118 | ***Lactuca sibirica* (L.) Maxim.** | Kyiv, Symon Petlura Str., 1, O.V. Fomin Botanical Garden of the Taras Shevchenko National University of Kyiv | - | - | A | first cases of escape from cultivation (Shynder, Kolomiychuk, et al., 2022);  POWO: ***L. sibirica*** |
| 119 | ***Klasea bulgarica* (Acht. et Stoj.) Holub.** | Dnipropetrovsk region, Apostolove district, near Tokivske village, in the valley of the Kamianka River | wet meadows | 30.05.2001, 16.05.2002, 15.07.2002 | N | (Kucherevskyi et al., 2009);  POWO: ***K. bulgarica*** |
| 120 | ***Pilosella* × *rubripilosella* (G. Schneider) Soják** [=*P. aurantiaca* (L.) F. Schultz et Sch. Bip. × *P. lactucella* ([Wallr.](https://en.wikipedia.org/wiki/Karl_Friedrich_Wilhelm_Wallroth)) P.D.Sell & C.West × *P. officinarum* Vaill.] | Carpathians: Beskydy and low mountain meadows | mountain meadows | - | N | (Tikhomirov, 2002);  POWO: ***P. stoloniflora* (Waldst. & Kit.) F.W.Schultz & Sch.Bip.** |
| 121 | ***Rudbeckia fulgida* Ait.** | 1) Zhytomyr region, Zhytomyr district, 0.2 km to W from a dacha massif in the village Barashivka;  Transcarpathian region, Uzhhorod: 2) on the Orthodox embankment;  3)Str. March, 8;  4) Sriblyasta Str.;  5) M. Yokoi Str.;  Transcarpathian region, Uzhhorod district: 6) near Barvinok village;  7) near Mynai village | 1) on the littered edge of a pine forest;  2) near the front garden of a private house;  3) under the fence of an abandoned building;  4) in a small ditch in front of the fence of a private house;  5) -;  6) on the territory of the cemetery between the graves and on the path;  7) on an abandoned plot of fruit orchard | 1) 13.09.2015;  2) 11.09.2021;  3) 13.09.2021;  4) 23.09.2021;  5) 30.10.2021;  6) 12.09.2021;  7) 2021 | A | first cases of escape from cultivation (Orlov & Shevera, 2021);  POWO: ***R. fulgida*** |
| 122 | ***Rudbeckia triloba* L.** | Kharkiv, Klochkivska Str., "Komsomolets" brick factory | along the road | 17.09. 2010 | A | (Zvyagintseva, 2015). A little later, several new localities of the species were discovered in different regions of the country (Shevera et al., 2020);  POWO: ***R. triloba*** |
| 123 | ***Symphyotrichum* × *salignum*** (Willd.) G.L.Nesom | Kyiv, M.M. Gryshko National Botanical Garden of the National Academy of Sciences of Ukraine | - | - | A | first cases of escape from cultivation (Shynder, 2019);  POWO: ***S.*× *salignum*** |
| 124 | ***Symphyotrichum squamatum* (Spreng.) G. L. Nesom** | Crimea: 1) Yalta vicinity, Lower Masandra, near hotel complex “Yalta-Inturyst”;  2) Yalta, Kotelnikova Str., 44°29'29"N, 34°08'56"E;  3) Yalta, 44°29'20.47"N, 34°09'44.85"E;  4) Yalta, Rudanskogo Str., 44°29'51"N, 34°10'21"E;  5) Hurzuf vicinity;  6) Hurzuf, 44°31'55"N, 34°16'22"E, recreation complex “Zhemchuzhyna Kryma”;  7) Alushta, 44°40'40"N, 34°25'15"E;  8) Sevastopol, upper Komyshova bay, 44°34'40"N, 33°26'05"E;  9) Sevastopol, Komyshova bay, 44°34'33.68"N, 33°26'10.10"E | 1) and 2) - ;  3) embankment, 5 m a.s.l.;  4) in the square, 14 m a.s.l.;  5) - ;  6) embankment, 5 m a.s.l.;  7) embankment, 5 m a.s.l.;  8) shipyard, 0 – 2 m a.s.l.;  9) shipyard | 1) 11.11.1974;  2) 7.10.2011;  3) 18.09.2014;  4) 15.09.2018;  5) 17.10.2010;  6) 16.09.2018;  7) 24.10.2014;  8) 29.07.2014;  9) 17.09.2014 | A | (Raab-Straube & Raus, 2019a);  POWO: ***S. subulatum* (Michx.) G.L. Nesom** var. *squamatum* (Spreng.) S.D.Sundb. |
| 125 | ***Taraxacum acervatulum* Rail.** | Lviv: 1) High Castle, 49º50’44”N, 24º02’06”E;  2) Lychakiv Cemetery, 49º49’56”N, 24º03’11”E | 1) urban lawn;  2) lawns at cemetery avenues | 1) 09.05.2017;  2) 08.05.2017 | N | (Nobis et al., 2020);  POWO: ***T. acervatulum*** |
| 126 | ***Taraxacum aequilobum* Dahlst.** | Ivano-Frankivsk region, Fraga village, 49º28’04’’N, 24º26’48’’E | roadside (on limestone soil) | 12.05.2017 | N | (Nobis et al., 2020);  POWO: ***T. aequilobum*** |
| 127 | ***Taraxacum amplum* Markl.** | Lviv: 1) city park, 49º50’13’’N, 24º01’28’’E;  2) Lychakiv Cemetery, at cemetery avenues, 49º49ʹ56ʺN, 24º03ʹ11ʺE;  3) Lviv region, Lelekhivka village, Yavorivskyi National Park, 49º57’01’’N, 24º41’25’’E  4) Ivano-Frankivsk region, Fraga village, 49º28’04’’N, 24º26’48’’E | 1) lawn;  2) lawns;  3) meadow;  4) roadside (on limestone soil) | 1) 08.05.2017;  2) 08.05.2017;  3) 11.05.2017  4) 12.05.2017 | N | (Nobis et al., 2020);  POWO: ***T. amplum*** |
| 128 | ***Taraxacum ancistrolobum* Dahlst.** | Lviv region, Khodorkivtsi village, 49º35’00.1’’N, 24º16’46.0’’E | marshy meadow | 12.05.2017 | N | (Nobis et al., 2020);  POWO: ***T. ancistrolobum*** |
| 129 | ***Taraxacum bellicum* Sonck** | Lviv region, Lelekhivka village, Yavorivskyi National Park, 49º56’45’’N, 23º41’21’’E | roadside in the forest | 11.05.2017 | N | (Nobis et al., 2020);  POWO: ***T. bellicum*** |
| 130 | ***Taraxacum collarispinulosum* Uhlemann** | Lviv, Czerniowiecka street, 49º50’13’’N, 24º00’00’’E | lawn | 09.05.2017 | N | (Nobis et al., 2020);  POWO: ***T. collarispinulosum*** |
| 131 | ***Taraxacum copidophyllum* Dahlst.** | Lviv region: 1) between Lozyno and Dubrovytsia, 49º57’21.4’’N, 23º48’45.7’’E;  2) Hodorkivtsi village, 49º35’00.1’N, 24º16’46.0’’E | 1) wet meadow;  2) wet pasture | 1) 11.05.2017;  2) 12.05.2017 | N | (Nobis et al., 2020);  POWO: ***T. copidophyllum*** |
| 132 | ***Taraxacum corynodes* G.E.Haglund** | Lviv region, between Lozyno and Dubrovytsia, 49º57’21.4’’N, 23º48’45.7’’E | wet meadow | 11.05.2017 | N | (Nobis et al., 2020);  POWO: ***T. corynodes*** |
| 133 | ***Taraxacum crocelliforme* Doll ex Štěpánek et Kirschner** | 1) Transcarpathian region, Chornohora Mts, E. slopes of Mt Dančer, near Mt Sheshul above Kvasy;  Ivano-Frankivsk region: 2) Chornohora Mts, in the valley of Breskulec below Pozhyzhevska Mts;  3) Chornohora Mts, in the valley of Breskulec below Pozhyzhevska Mts, above the chalet of the Ukrainian Academy of Science | humid habitats on the shores of alpine lakes, near springs and along brooks;  1) 1600–1700 m;  2) ~1800 m;  3) ~1500 m | 1) 06.1990;  2) 06.1990;  3) 06.1990 | N | first described for science (Štěpánek et al., 2023);  POWO: - |
| 134 | ***Taraxacum dentatum* Kirschner & Štĕpánek** | Lviv region: 1) between Lozyno and Dubrovytsia, 49º57’21’’N, 23º48’45’’E;  2) Ivano-Frankove village, 49º54’36’’N, 23º44’33’’E, in the Vereshchytsia River valley;  3) Novosilky village, 49º38’42’’N, 24º01’19’’E | 1) wet meadow;  2) meadow;  3) wet pasture | 1) 11.05.2017;  2) 11.05.2017;  3) 08.05.2017 | N | (Nobis et al., 2020);  POWO: ***T. dentatum*** |
| 135 | ***Taraxacum egnatiae* Sonck** | Crimea, Yalta, slopes above the town, near “Gruševaja poljana”, | along road in pine woodlands | 24.05.1989 | N | (Štěpánek & Kirschner, 2018, 2022a);  POWO: ***T. egnatiae*** |
| 136 | ***Taraxacum gelertii* Raunk** | 1) Lviv region, Lelekhivka village, 49º56’45’’N, 23º41’21’’E;  2) Stradch village, 49º54’02’’N, 23º45’34’’E;  3) Lviv, Lyczakiv Cemetery, 49º49’58’’N, 24º03’12’’E | 1) marshy meadow;  2) meadow;  3) lawn | 1) 11.05.2017;  2) 11.05.2017  3) 08.05.2017 | N | (Nobis et al., 2020);  POWO: ***T. gelertii*** |
| 137 | ***Taraxacum* *gracilens* Dahlst.** | Crimea: E margin of Simferopol city, near Balaja village | calcareous slope | 02.05.1984 | N | (Štěpánek & Kirschner, 2014);  POWO: ***T. gracilens*** |
| 138 | ***Taraxacum infuscatum* H. Øllg.** | Lviv: 1) Lychakiv Cemetery, 49º49’56’’N, 24º03’11’’E;  2) High Castle, 49º50’44’’N, 24º02’06’’E;  3) Lviv region, Lelekhivka village, Yavorivskyi National Park, 49º57’01’’N, 24º41’25’’E | 1) lawn;  2) lawn;  3) forest road | 1) 08.05.2017;  2) 09.05.2017;  3) 11.05.2017 | N | (Nobis et al., 2020);  POWO: ***T. infuscatum*** |
| 139 | ***Taraxacum ingens* Palmgr** | Lviv region, Stradch village, 49º50’44’’N, 24º02’06’’E | meadow | 11.05.2017 | N | (Nobis et al., 2020);  POWO: ***T. ingens*** |
| 140 | **Taraxacum jailae Štěpánek & Kirschner** | Crimea, Alushta district, 44.716667°N, 34.433334°E | jaila | 26.05.1989 | N | first described for science (Štěpánek & Kirschner, 2022b);  POWO: no data |
| 141 | ***Taraxacum lucidum* Dahlst.** | Lviv region, between Lozyno and Dubrovytsia, 49º57’21.4’’N, 23º48’45.7’’E | wet meadow | 11.05.2017 | N | (Nobis et al., 2020);  POWO: ***T. lucidum*** |
| 142 | ***Taraxacum × mesohalobium* Kirschner et Štěpánek** (= *T. bessarabicum* (Hornem.) Hand.-Mazz. *× T. salsum* Kirschner et Štěpánek) | Crimea, isthmus of Arabatska Strilka, in the vicinity of Strilkove village, 100-200 m from the Azov Sea coast | salt meadows | 1989 | N | first described for science (Kirschner & Štěpánek, 1998);  POWO: ***T. × mesohalobium*** |
| 143 | ***Taraxacum obesum* Štěpánek et Kirschner** | Ivano-Frankivsk region: 1) Chornohora Mts, Mt Pozhyzhevska, left from Breskul Mt, 48°8'39"N, 24°31'23"E;  2) Chornohora Mts, Mt Pozhyzhevska, next to the station;  3) Chornohora Mts, Mt Breskul, waterfall I [on Prut R.] | moist subalpine and alpine stony grasslands and along streams, at the elevation between 1500 and 1800 m. | 1) 08.1923;  2) 6.08.1925;  3) 29.08.1925 | N | first described for science (Štěpánek et al., 2023);  POWO: - |
| 144 | ***Taraxacum paucilobum* Hudziok** | Lviv region: 1) Novosilky village, 49º38’42’’N, 24º01’19’’E;  2) Lelekhivka village, 49º56’45’’N, 23º41’1’’E;  3) Stradch village, 49º53’58’’N, 23º45’19’’E;  4) between Lozyno and Dubrovytsia, 49º57’21’’N, 23º48’45’’E;  5) Ivano-Frankove village, 49º54’36’’N, 23º44’33’’E, in the Vereshchytsia River valley | 1) wet pasture;  2) wet meadow;  3) wet meadow;  4) wet meadow;  5) wet meadow | 1) 08.05.2017;  2) 11.05.2017;  3) 11.05.2017;  4) 11.05.2017;  5) 11.05.2017 | N | (Nobis et al., 2020);  POWO: ***T. paucilobum*** |
| 145 | ***Taraxacum pawlowskii* van Soest** | Ivano-Frankivsk region, Chornohora Mts, Mt Breskul | humid, stony alpine grasslands along shores of lakes, at the foot of mountain slopes, along tracks and paths, in the vicinity of mountain chalets; usually above 1,800 m but occasionally occurs at lower altitudes along brooks | 17.06.1925 | N | (Štěpánek et al., 2023);  POWO: ***T. pawlowskii*** |
| 146 | ***Taraxacum perenne* Kirschner et Štěpánek** | Crimea, Sevastopol, in the valley of Baidarska dolyna, near Orlyne village | subhalophilous communities | 1989 | N | first described for science (Kirschner & Štěpánek, 1998);  POWO: ***T. perenne*** |
| 147 | ***Taraxacum plumbeum* Dahlst.** | Lviv region: 1) Velyka Volya village, 49º32’13’’N, 24º01’14’’E;  2) Stradch village, 49º53’58’’N, 23º45’19’’E;  3) Lelekhivka village, Yavorivskyi National Park, 49º56’45’’N, 23º41’21’’E | 1) dry grassland with *Orchis morio*;  2) meadow;  3) meadow and sandy roadside in the forest | 1) 10.05.2017;  2) 11.05.2017;  3) 11.05.2017 | N | (Nobis et al., 2020);  POWO: ***T. plumbeum*** |
| 148 | ***Taraxacum portentosum* Kirschner & Štĕpánek** | Lviv region: 1) Novosilky village, 49º38’42’’N, 24º01’19’’E;  2) Stradch village, 49º54’02’’N, 23º45’34’’E;  3) between Lozyno and Dubrovytsia, 49º57’02’’N, 23º48’45’’E | 1) wet pasture;  2) wet meadow;  3) wet meadow | 1) 08.05.2017;  2) 11.05.2017;  3) 11.05.2017 | N | (Nobis et al., 2020);  POWO: ***T. portentosum*** |
| 149 | ***Taraxacum salsum* Kirschner et Štěpánek** | 1) Crimea, isthmus of Arabatska Strilka, in the vicinity of Strilkove village, 100-200 m of the Azov Sea coast;  2) Kherson region, Henichesk district, Strilkove village, shore of Syvash salt lakes | 1) and 2): flat open grassy shores with soils of high salinity, rich in halophilous vegetation | 1) 1989;  2) 1989 | N | first described for science (Kirschner & Štěpánek, 1998);  POWO: ***T. salsum*** |
| 150 | ***Taraxacum sinuatum* Dahlst.** | Lviv region, between Lozyno and Dubrovytsia, 49º57’21.4’’N, 23º48’45.7’’E | wet meadow | 11.05.2017 | N | (Nobis et al., 2020);  POWO: ***T. sinuatum*** |
| 151 | ***Taraxacum subhuelphersianum* M.P.Chr** | Lviv, Lychakiv Cemetery, 49º49’56’’N, 24º03’11’’E | lawn | 08.05.2017 | N | (Nobis et al., 2020);  POWO: ***T. subhuelphersianum*** |
| 152 | ***Taraxacum telmatophilum* Kirschner & Štĕpánek** | Lviv region, Novosilky village, 49º38’42’’N, 24º01’19’’E | waterlogged pasture | 08.05.2017 | N | (Nobis et al., 2020);  POWO: ***T. telmatophilum*** |
| 153 | ***Taraxacum undulatiforme* Dahlst.** | Lviv region, Velyka Volya village, 49º32’13’’N, 24º01’14’’E | dry grassland with *Orchis morio* | 10.05.2017 | N | (Nobis et al., 2020);  POWO: ***T. undulatiforme*** |
| 154 | ***Taraxacum undulatum* H.Lindb. & Marklund** | Lviv region: 1) Stradch village, 49º54’02.2’’N, 23º45’34.0’’E;  2) Ivano-Frankove village, 49º54’36’’N, 23º44’33’’E, over the Vereshchytsia River;  3) between Lozyno and Dubrovytsia, 49º57’21.4’’N, 23º48’45.7’’E;  4) Khodorkivtsi village, 49º35’00.1’’N, 24º16’46.0’’E | 1) grazed meadow;  2) meadow;  3) wet meadow;  4) wet pasture | 1) 11.05.2017;  2) 11.05.2017;  3) 11.05.2017;  4) 12.05.2017 | N | (Nobis et al., 2020);  POWO: ***T. undulatum*** |
| 155 | ***Taraxacum ursinum* Štěpánek et Kirschner** | Ivano-Frankivsk region, Chornohora Mts, Mt Breskul | rocky and stony alpine slopes, along mountain paths, in subalpine grasslands, more often on limestone, less often on granite, usually between (1300–) 1600–2000 m | 20.08.1990 | N | first described for science (Štěpánek et al., 2023);  POWO: - |
| 156 | ***Taraxacum venustum* Dahlst.** | Ivano-Frankivsk region: 1) Chornohora Mts, summit area of Pip Ivan Chornohorsky, 48°2'50"N, 24°37'39"E;  2. Chornohora Mts, between Mt Turkul and Mt Tomnatek | 1) 2026 m;  2) ~1900 m | 1) 1991;  2) 30.06.1991 | N | The species is considered as an aggregate for the flora of Ukraine, without detalization (Štěpánek et al., 2023);  POWO: ***T. venustum*** |
| 157 | ***Taraxacum vidlense* Doll** | Ivano-Frankivsk region, Chornohora Mts: 1) between Chornohora and Stohorec;  2) valley of Breskulec Brook, below Mt Pozhyzhevska | alpine and subalpine grasslands, humid scree, margins of paths and also tall-herb communities along streams | 1) 6.06.1913;  2) 06.1990 | N | (Štěpánek et al., 2023);  POWO: ***T. vidlense*** |
| 158 | ***Tyrimnus leucographus* (L.) Cass.** | Crimea, Sevastopol: 1) detour highway, 1 km from Balaklava junction, 44°32’05”N, 33°34’00”E;  2) Karanska Balka valley | 1) crumbling slope undercut during highway construction, elev. 160 m;  2) - | 1) 28.07.2014;  2) 2010 | N | (Seregin, Yevseyenkov, et al., 2015);  POWO: ***T. leucographus*** |
| ***Balsaminaceae* A.Rich.** | | | | | | |
| 159 | ***Impatiens balfourii* Hook. f.** | 1) Zhytomyr region, Yemilchyne district, near Kyianka village;  2) Transcarpathian region, in Vuzlove village (“Batiovo” railway station);  3) Transcarpathian region, Tiachiv district, in Neresnytsya village, along the Luzhanka River | 1) thinned and well-drained alder forest;  2) near private residences;  3) on the river bank | 1) 15.10.2012;  2) 17.08.2013;  3) 18.07. 2013 | A | (Orlov et al., 2014);  POWO: ***I. balfourii*** |
| ***Berberidaceae* Juss.** | | | | | | |
| 160 | ***Berberis* *vulgaris* subsp. *spryginii* Tzvelev** | Crimea, near Sevastopol, near Omeha settlement | on the clay seashore | 23.08.1962 | N | first described for science (Tzvelev, 2001b);  POWO: ***B. spryginii* (Tzvelev) Tatanov & Vasjukov** |
| ***Boraginaceae* Juss.** | | | | | | |
| 161 | ***Amsinckia calycina* (Moris) Chater** | Odesa, harbor | on dense soil with stains of oil products | 1992 – 1998 (?) | A | (Vasyljeva & Kovalenko, 2000); POWO: ***A. calycina*** |
| 162 | ***Buglossoides incrassata* (Guss.) I.M. Johnst. subsp. *incrassata*** | Crimea, Sevastopol, Cape Aya Nature Reserve: 1) Biller ridge, Demir-Capu tract;  2) Inzhyr tract;  3) SW slope of Biller ridge;  4) Sevastopol, Balaklava vicinity, Vitmer ravine, 44°30'03"N, 33°37'44"E;  5) Sevastopol, Balaklava vicinity, Vitmer ravine, 44°30'04"N, 33°37'33"E;  6) Sevastopol, Mekenzi mountains, 44°39'15.30"N, 33°41'22.06"E | 1) and 2) - ;  3) 600 m, gravelly slope;  4) 175 m, open *Juniperus excelsa* M. Bieb. forest;  5) 140 m;  6) clearing in *Quercus pubescens* Willd. and *Carpinus orientalis* Mill. forest | 1) 24.04.2010;  2) 13.04.2012;  3) 28.04.2012;  4) 10.06.2019;  5) 27.04.2021;  6) 2.04.2023 | N | (Raab-Straube & Raus, 2024);  POWO: ***B. incrassata* subsp. *incrassata*** |
| 163 | ***Buglossoides incrassata* (Guss.) I.M. Johnst. subsp. *splitgerberi* (Guss.) E. Zippel & Selvi** | 1) Ternopil;  2) Cherkasy region, Uman’;  3) near Lviv | 1) field;  2) in the fields;  3) - | 1) 09.05.1993;  2) 30.05.1990;  3) 1860 | N | (Raab-Straube & Raus, 2020, p. 12);  POWO: ***B. incrassata* subsp. *splitgerberi*** |
| 164 | ***Eritrichium nanum*** (L.) Gaudin | Ivano-Frankivsk region, Kalush district, Skeli Dovbusha, 49.04240º N, 23.68305º E | on rock outcrops, alt. 639 m | 15.07.2014 | A | only 3 plants, probably intentional release (Moysiyenko et al., 2023);  POWO: ***E. nanum*** |
| ***Brassicaceae* Burnett** | | | | | | |
| 165 | ***Arabis procurrens* Waldst. & Kit.** | Kyiv, Symon Petlura Str., 1, O.V. Fomin Botanical Garden of the Taras Shevchenko National University of Kyiv | - | - | A | first cases of escape from cultivation (Shynder, Kolomiychuk, et al., 2022);  POWO: ***A. procurrens*** |
| 166 | ***Cardamine marholdii* Tzvel.** | Transcarpathian region: 1) Tiachiv district, Gorgany range, the Perednia Mount;  2) the Hoverla Mount  3) Svydovets;  4) Rakhiv district, along the Bila River;  5) Chornohora, the Gesa Mount;  6) the Blyznytsya Mount;  7) the Turkul Mount, the Nesamovyte Lake | 1) swampy stream bank, 1400 m above s.l.;  2) 1300 m above s.l.;  3) -;  4) along the river;  5) hillside, 1400 m above s.l.;  6) 1750 m above s.l.;  7) lake shore | 1) 12.08.1949;  2) 22.07.1940, 20.07.1959;  3) 23.07.1947;  4) 24.05.1947;  5) 11.09.1949;  6) 13.09.1949;  7) 05.08.1977 | N | first described for science (Tzvelev, 2003a);  POWO: ***C. marholdii*** |
| 167 | ***Cardamine occulta* Hornem.** | 1) Cherkasy region, Uman district, Sharyn, 48.617397ºN, 30.236494ºE;  2) Chernivtsi region, Chernivtsi, Herzena [Volodymyra Velykoho] St., 48.282222ºN, 25.952500ºE;  3) Chernivtsi region, Chernivtsi, Ruska St., 35, in the yard of the Consistory of the Chernivtsi-Bukovyna Diocese, 48.290889ºN 25.943944ºE;  4) Chernivtsi region, Dnistrovskyi district, the outskirts of the village Ivanivtsi, Ivanivtsi basic nursery, Sokyriany forestry, 48.472250ºN 27.043306ºE;  5) Kyiv, Tereshchenkivska St., 50.442567ºN, 30.514793ºE | 1) near the highway, in a garden and flower arrangement, on gravel, abundantly;  2) a flower bed with decorative gravel;  3) on the flowerbeds and in the cracks between the pavement tiles;  4) on agrofibre between pots with seedlings;  5) in a flower pot | 1) 28.04.2023;  2) 11.10.2022;  3) 03.11.2022;  4) 03.12.2022;  5) 11.11.2021, 10.11.2022 | A | (Shynder et al., 2024);  POWO: ***C. occulta*** |
| 168 | ***Cardamine submatthioli* (Tzvel.) Tzvel.** | Regions: Chernivtsi, Chernihiv, Ivano-Frankivsk, Lviv, and Transcarpathian | wet meadows | - | N | (Tzvelev, 2003a);  POWO: ***C. pratensis* L. subsp. *submatthioli* Tzvelev** |
| 169 | ***Cardaria chalepensis* (L.) Hand.-Mazz.** | Kherson, on the territory of the cotton mill | on the asphalt site next to the railway tracks | 18.08.1996, 07.08.1997 | A | (Mosyakin & Moysienko, 1999);  POWO: ***Lepidium chalepense* L.** |
| 170 | ***Diplotaxis catholica* (L.) DC.** | Kyiv, M.M. Gryshko National Botanical Garden of the National Academy of Sciences of Ukraine | - | 21.05.2020 | A | first cases of escape from cultivation (Shynder, Doiko, et al., 2022);  POWO: ***D. catholica*** |
| 171 | ***Goldbachia torulosa* DC.** | Kyiv, railway station “Kyiv-Tovarnyi” | along railway | 15.05.1990 | A | This species was collected and identified as *G. laevigata* (M. Bieb.) DC. by S. Mosyakin, but according to German (German, 2022) the identification was incorrect and these found plants belong to *G. torulosa*; consequently, *G. torulosa* should be listed in the flora of Ukraine, and *G. laevigata* should be excluded; however, these plants reported only once, then disappeared (Mosyakin & Fedoronchuk, 1999);  POWO: ***G. torulosa*** |
| 172 | ***Malcolmia graeca* Boiss. et Sprun.** | Crimea, near Feodosia | open rocky or gravelly places | early 20^th^ century | A | (Tzvelev & Geltman, 2012)  POWO: ***M. graeca*** |
| 173 | ***Olimarabidopsis pumila* (Stephan) Al-Shehbaz & al.** | Crimea, Sovetskiy district, 8 km N of Dmytrivka, coast of Syvash gulf at the border of “Prysyvashskyi”preserve, 45°33'53"N, 35°03'47"E | saline land, 0 m a.s.l. | 21.04.2019 | N | (Raab-Straube & Raus, 2019b);  POWO: ***O. pumila*** |
|  | *Velarum leiocarpum* (DC.) Tzvel | Uzhhorod | - | 04.07.1960 | N | (Tzvelev, 2003a);  POWO: *Sisymbrium officinale* (L.) Scop., already known for Ukraine (Mosyakin & Fedoronchuk, 1999) |
| ***Cactaceae* Juss.** | | | | | | |
| 174 | ***Opuntia engelmannii* Salm-Dyck ex Engelm. var. *lindheimeri* (Engelm.) B.D. Parfitt & Pinkava** | southern coast of Crimea | *Asplenietea trichomanis* (Br.-Bl. in Meier et Br.-Bl 1934) Oberdorfer 1977, *Koelerio­Corynephoretea* Klika in Klika et Novák 1941, *Festuco­Brometea* Br.-Bl. et Tx. ex Soó 1947, *Artemisietea vulgaris* Lohmeyer et al. in Tx. ex von Rochow 1951 | - | A | first cases of the species naturalization (Bagrikova & Perminova, 2022; Yena, 2012);  POWO: ***O. engelmannii*** |
| 175 | ***Opuntia fragilis* (Nutt.) Haw.** | southern coast of Crimea: Haspra | *Koelerio-Corynephoretea*, *Cisto-Micromerietea julianae* Oberdorfer 1954 | - | A | first cases of the species naturalization (Bagrikova et al., 2021; Bagrikova & Perminova, 2022; Bagrikova & Ryff, 2014);  POWO: ***O. fragilis*** |
|  | *Opuntia fulgida* Engelm. | Crimea | - | - | A | first cases of the species naturalization (Yena, 2012), but this species was later reidentified as *O. fragilis* (Bagrikova & Ryff, 2014);  POWO: *Cylindropuntia fulgida* (Engelm.) F.M. Knuth |
| 176 | ***Opuntia macrorhiza* Engelm.** | southern coast of Crimea | *Cisto­Micromerietea* *julianae*, *Koelerio ­ Corynephoretea*, *Festuco­Brometea* | - | A | first cases of the species naturalization (Bagrikova et al., 2021; Bagrikova & Perminova, 2022; Bagrikova & Ryff, 2014);  POWO: ***O. macrorhiza*** |
| 177 | ***Opuntia phaeacantha* Engelm.** | southern coast of Crimea: near Kurortne | *Pegano harmalae­Salsoletea vermiculatae* Br.-Bl. et de. Bols 1958, *Thlaspietea rotundifolii* Br.-Bl. 1948, *Onosmo polyphyllae ­ Ptilostemonetalia* Korzhenevsky 1990 | - | A | first cases of the species naturalization (Bagrikova & Perminova, 2022; Bagrikova & Ryff, 2014; Yena, 2012);  POWO: ***O. phaeacantha*** |
| 178 | ***Opuntia polyacantha* Haw.** | southern coast of Crimea | - | - | A | first cases of the species naturalization (Bagrikova et al., 2021; Bagrikova & Perminova, 2022; Bagrikova & Ryff, 2014);  POWO: ***O. polyacantha*** |
| 179 | ***Opuntia tortispina* Engelm. et J.M. Bigelow** | southern coast of Crimea | - | - | A | first cases of the species naturalization (Bagrikova et al., 2021; Bagrikova & Perminova, 2022; Bagrikova & Ryff, 2014);  POWO: ***O. tortispina*** |
| 180 | ***Opuntia tunoidea* Gibbes.** | southern coast of Crimea: it is found quite often from Foros to Malorichynske, rarely in the vicinity of the Meganom Peninsula | *Koelerio­Corynephoretea*, *Cisto­Micromerieteajulianae*, *Thero­Brachypodietea* Br.-Bl. ex O. de Bolòs y Vayreda 1950 | - | A | first cases of the species naturalization (Bagrikova et al., 2021; Bagrikova & Perminova, 2022; Bagrikova & Ryff, 2014);  POWO: ***O. tunoidea*** |
| ***Campanulaceae* Juss.** | | | | | | |
| 181 | ***Legousia perfoliata* (L.) Britt.** | Odesa, Khlibna harbor, near the grain elevator | near the wall of the house | 06.1994 – 1996 (?) | A | (Vasyljeva & Kovalenko, 2000);  POWO: ***Triodanis perfoliata* (L.) Nieuwl.** |
| ***Cannabaceae* Martinov** | | | | | | |
| 182 | ***Celtis caucasica*** Willd. | Odesa: 1) 46.444784°N, 30.764291°E, outside Botanical Garden of I.I. Mechnikov Odesa National University;  2) 46.44079°N, 30.76807°E, Botanical Garden of I.I. Mechnikov Odesa National University, in arboretum;  3) Uzhhorod, 48.618504°N, 22.305502°E, between Botanical Garden of Uzhhorod National University and Uzhhorod children railway;  4) Kyiv, M.M. Gryshko National Botanical Garden | 1) 46 m, along fence;  2) - ;  3) in anthropogenic habitats;  4) - | 1) 16.09.2022;  2) 17.09.2022;  3) 5.09.2022, 16.09.2022, 04.06.2023;  4) 16.08.2023 | A | The species was reported before only as a cultivated within Ukraine, so it is the first documented facts of its escaping (Raab-Straube & Raus, 2024);  POWO: ***C. caucasica*** |
| 183 | ***Celtis occidentalis* L.** var. *pumila* (Pursh) A. Gray | 1) Chernivtsi region, near Khreshchatyk village, along the Dniester River;  2) Khmelnytskyi region, Kamianets-Podilskyi district, near Ustya village, along the Dniester and the Smotrych rivers (3 locality) | 1) and 2) steep river banks | - | A | The species was reported before only as a cultivated within Ukraine, so it is the first documented facts of its escaping (Ya. Didukh & Boratynski, 2002);  POWO: ***C. occidentalis*** |
|  | *Celtis planchoniana* K.I. Chr | 1) Odesa, Chkalov Sanatorium Park, 46.445385°N, 30.768454°E;  2) Odesa, 46.445112°N, 30.768772°E | 1) in underwood;  2) top of slope above sea near park | 1) 17.09.2022;  2) 26.09.2022 | N / A | The cases of escaping from cultivation have been reported (Raab-Straube & Raus, 2024), however this species is know as a native one from Crimea under the name *C. glabrata* Steven ex Planch. (Mosyakin & Fedoronchuk, 1999)  POWO: *C. glabrata* |
| 184 | ***Humulus japonicus* Siebold. et Zucc.** | 1) Uzhhorod, Park “Pidzamkovyi”, along the Uzh River;  2) Kyiv, Syrets;  3) Kyiv, Zvirynets;  4) Kyiv region, Bila Tserkva, Dendrological Park “Olexandriia” | ruderal places | 1) 16.05.2002;  2) 15.09. 1944;  3) 26.08.1944-45;  4) 11.10.1955 | A | first cases of escape from cultivation (Andryk et al., 2010);  POWO: ***H. scandens* (Lour.) Merr.** |
| ***Caprifoliaceae* Juss.** | | | | | | |
| 185 | ***Cephalaria gigantea* (Ledeb.) Bobrov** | Vinnytsia, Park “Podillya”, 49°12′40.921″N, 28°24′58.869″E | on the edge of a forest massif with dominance of *Fraxinus excelsior* L. | 07.2016 | A | (Kuzemko et al., 2019);  POWO: ***C. gigantea*** |
| 186 | ***Lomelosia brachiata* (Sm.) Greuter et Burdet** | Crimea, Sevastopol, Horna Height, Berman’s ravine | - | 31.05.2017 | N | (Bondareva et al., 2018);  POWO: ***L. brachiata*** |
| 187 | ***Lomelosia divaricata* (Jacq.) Greuter et Burdet** | Crimea, Sevastopol, Yukharina ravine | - | 31.05.2017 | N | (Bondareva et al., 2018);  POWO: ***L. divaricata*** |
| 188 | ***Lonicera* *maackii* (Rupr.) Maxim.** | Kyiv: 1) M.M. Gryshko National Botanical Garden of the National Academy of Sciences of Ukraine, 50.419397°N, 30.556205°E;  2) O,V, Fomin Botanical Garden of the Taras Shevchenko National University of Kyiv, 50.441413°N, 30.505529°E | 1), 2) - | 1) 08.06.2022;  2) 26.10.2022 | A | first cases of escape from cultivation (Shynder et al., 2024);  POWO: ***L. maackii*** |
| 189 | ***Lonicera × notha* Zabel** (= *L. ruprechtiana* Regel × *L. tatarica* L.) | Kyiv: 1) M.M. Gryshko National Botanical Garden of the National Academy of Sciences of Ukraine: 50.4119, 30.5628, 16.05.2019; 50.4124, 30.5666; 50.41178, 30.56620; 50.41115, 30.55977;  2)Kyiv, Syrets Arboretum;  Kyiv region, Bila Tserkva city, Arboretum “Olexandria”: 49.81648, 30.07003; 49.81019, 30.06653; 49.80446, 30.05657 | 1), 2) and 3) thickets of shrubs | 1) 13, 14 and 16.05.2019;  2) - ;  3) 22.05.2019 | A | first cases of escape from cultivation (Shynder et al., 2020);  POWO: ***L. × muendeniensis* Rehder** (= *L. morrowii* A. Gray × *L. ruprechtiana* × *L. tatarica*) |
| 190 | ***Lonicera ruprechtiana* Regel** | Kyiv: 1) M.M. Gryshko National Botanical Garden of the National Academy of Sciences of Ukraine, exposition plots “Far East”, “Caucasus”, “Middle Asia”, “Altai and West Siberia”;  2) Syrets’ Arboretum | 1) and 2) thickets of shrubs | - | A | first cases of escape from cultivation (Shynder et al., 2020);  POWO: ***L. ruprechtiana*** |
|  | *Valeriana dacica* Porcius | Ukrainian Carpathians | - | - | N | There is no confirmed data about the growth of the species within Ukraine, only the author’s suggestion (Krahulec et al., 2023);  POWO: *V. tripteris* L. subsp. *tripteris* |
| ***Caryophyllaceae* Juss.** | | | | | | |
| 191 | ***Arenaria martrinii* Tzvel.** | Transcarpathian region, Velykyi Bereznyi district, to 12-18 km to NW from the settlement | on sandstone outcrops | 1985 | N | The new replacement name is reported here, however the species has never been mentioned under other names for the flora of Ukraine. (Tzvelev, 2000);  POWO: ***A. serpyllifolia* L. subsp. *serpyllifolia*** |
| 192 | ***Holosteum* *umbellatum* L. subsp. *klopotovii* Tzvelev** | Crimea, Kerch Peninsula | barrows | 05.04.1906 | N | first described for science (Tzvelev, 2004);  POWO: ***H. klopotovii* (Tzvelev) Tzvelev** |
|  | *Oberna behen* (L.) Ikonn. subsp. *carpatica* (Zapał.) Tzvelev | Carpathians | Above 1000 m above s.l. | - | N | first described for science (Tzvelev, 2002);  POWO: *Silene behen* L., already known for Ukraine (Mosyakin & Fedoronchuk, 1999) |
| 193 | ***Otites × klopotovii* Tzvelev** (= *O. borysthenica* (Grun.) Klok. × *O. densiflora* D’Urv. Grossh.) | Crimea, Kerch Peninsula, Katerlez | on limestone rocks | 22.06.1905 | N | first described for science (Tzvelev, 2001a);  POWO: *O. × klopotovii* (an unplaced name) |
|  | *Otites sibiricus* (L.) Raf. subsp. *kleopovii* Tzvelev | Odesa region | - | - | N | first described for science (Tzvelev, 2001a);  POWO: *Silene sibirica* (L.) Pers., already known in Ukraine (Mosyakin & Fedoronchuk, 1999) |
| 194 | ***Sagina maritima* G. Don** | Crimea, Sevastopol, on the northwestern shore of the Kozacha Bay | on a narrow sandy strip under a low abrasion ledge | 2010 | N | (Yena et al., 2011);  POWO: ***S. maritima*** |
| 195 | ***Sagina schiraevskii* Tzvelev** | 1) Luhansk region, near Starobilsk;  2) Kherson region, Khola Prystan | 1) on wet sand;  2) - | 1) 08.1904;  2) 03.07.1906 | N | first described for science (Tzvelev, 2002);  POWO: ***S. micropetala* Rauschert** |
| 196 | ***Spergularia syvaschica* Tzvelev** | 1) Crimea, Lenin district, Plavni village (between Pisochne village and Shcholkine);  2) Kherson region, Henichesk district, the Kuyuk-Tuk Island | 1) on the sands;  2) along the banks of the Sivash | 1) 13.07.1954;  2) 24.06.1931 | N | first described for science (Tzvelev, 2000);  POWO: ***S. syvaschica*** |
| 197 | ***Stellaria ruderalis* M. Lepší, P. Lepší, Z. Kaplan et P. Koutecký** | Lviv region, Pustomyty district, Dmytre village, near the cemetery, 49.60507°N, 23.87046°E | at roadsides and arable field edges in a semiruderal habitat of the alliance *Aegopodion podagrariae*, alt. 270 m a.s.l | 20.05.2020 | N | (Novikov et al., 2020);  POWO: ***S. ruderalis*** |
| ***Celastraceae* R.Br.** | | | | | | |
| 198 | ***Celastrus orbiculatus* Thunb.** | Lviv region: 1) Sokal district, 3 km to W from Hirnyk setllemet;  2) western outskirts of the town Sokal; the left bank of the Zakhidnyi Buh River | 1) in the pine forest near the path;  2) on the dam | 1) 23.07. 2008  2) 03.07. 2009 | A | (Kuziarin, 2009);  POWO: ***C. orbiculatus*** |
| 199 | ***Celastrus scandens* L.** | Cherkasy region, Korsun-Shevchenkivskyi district, on the territory of the Rizanyi Yar protected tract | an ancient oak forest of natural origin | 09.2012, 05.2013 | A | The first case of escape from cultivation (Tyshchenko et al., 2013);  POWO: ***C. scandens*** |
| ***Cleomaceae* Airy Shaw** | | | | | | |
| 200 | ***Polanisia dodecandra* (L.) DC. subsp. *trachysperma* (Torr. & A.Gray) Iltis** | Kyiv, Symon Petlura Str., 1, O.V. Fomin Botanical Garden of the Taras Shevchenko National University of Kyiv | - | - | A | first cases of escape from cultivation (Shynder, Kolomiychuk, et al., 2022);  POWO: there is no data about this subspecies, but this species is noted as ***Cleome dodecandra* L.** |
| ***Crassulaceae* J.St.-Hil.** | | | | | | |
| 201 | ***Aizopsis aizoon* (L.) Grulich** | Chernihiv, Sviate tract | in the ditch of the forest road | 24.06.2007 | A | (Zavjalova, 2008);  POWO: ***Phedimus aizoon* (L.) 't Hart** |
| 202 | ***Sedum sediforme* (Jacq.) Pau** | Crimea:1) near Sevastopol;  2) S coast of Crimea, near Foros, Sevastopol–Yalta highway, 44.39°N, 33.78°E;  3) S coast of Crimea, near Foros, Sevastopol–Yalta highway, 44.40°N, 33.80°E;  4) E outskirts of Foros, 44.40°N, 33.80°E;  5) above Foros, old abandoned road to Baydarskyi pass, 44.39°N, 33.78°E;  6) vicinity of Sevastopol, foot of Mount Gasfort, near Sevastopol–Yalta highway, 44.53°N, 33.66°E;  7) vicinity of Foros, between Sanatornoye and Oliva, near Sevastopol–Yalta highway, 44.41°N, 33.84°E | 1) - ;  2) 165  m, roadside;  3) 165  m;  4) 60 m, roadside;  5) 180 m, roadside;  6) 70  m;  7) 210 m | 1) - ;  2) 19.09.2018;  3) 05.07.2020;  4) 12.07.2020;  5) 12.05.2021;  6) 11.07.2021;  7) 23.08.2021 | A | first cases of escape from cultivation (Raab-Straube & Raus, 2021b);  POWO: ***Petrosedum sediforme* (Jacq.) Grulich** |
| ***Droseraceae* Salisb.** | | | | | | |
| 203 | ***Drosera filiformis* Raf.** | To N from Kyiv | in the swamp near the reservoir | - | A | It is reported an unauthorized case of this species cultivation within the natural ecosystem with its successful reproductivity (Shiyan, 2018);  POWO: ***D. filiformis*** |
| ***Euphorbiaceae* Juss.** | | | | | | |
| 204 | ***Euphorbia glyptosperma* Engelm.** | Crimea, Dzhankoy district, railway station Solone Ozero, 45°53'00"N, 34°26'57"E | on railway tracks, 12 m a.s.l. | 02.10.2011 | A | (Raab-Straube & Raus, 2019a);  POWO: ***E. glyptosperma*** |
| 205 | ***Euphorbia hirsuta* L.** | Crimea, peninsula Meganom, Kapselska bay | on the sandy beach | 15.09.2004 | N | (Geltman & Shatko, 2012);  POWO: ***E. hirsuta*** |
| 206 | ***Euphorbia nutans* Lag.** | Crimea: 1) Bakhchysarai district, Mostove village, near railway bridge across Kacha river, 44°43'28''N, 33°48'47''E;  2) Bakhchysarai district, Zaliznychne village, 44°43'27.90''N, 33°48'46.98''E | 1) on railway tracks and railway embankment;  2) on edge of railway | 1) 11.09.2021;  2) 12.09.2021, 20.11.2022 | A | (Raab-Straube & Raus, 2023);  POWO: ***E. nutans*** |
| 207 | ***Euphorbia prostrata* Aiton** | Crimea, Yalta vicinity, Nikita, Nikitsky Botanical Garden, Nyzhniy Park, Palm Alley, 44°30'32.00"N, 34°14'02.60"E | shaded flower bed under regular watering, 105 m a.s.l. | 19.07.2018 | A | (Raab-Straube & Raus, 2019a);  POWO: ***E. prostrata*** |
| 208 | ***Euphorbia serpens* Kunth** | 1) Crimea, Livadia, 44.47589ºN,34.15524ºE;  2) Kherson region, Beryslav district, Shylova ravine, 46.81980ºN, 33.33314ºE;  3) Odesa region, Odesa city, Malyi Fontan, Shampanskyi line, 46.452317ºN, 30.758983ºE | 1) alt. 14 m;  2) roadside, alt. 15 a.s.l.;  3) alt. 47 m a.s.l. | 1) 20.07.2021;  2) 18.08.2021;  3) 26.09.2022 | A | (Moysiyenko et al., 2023);  POWO: ***E. serpens*** |
| ***Fabaceae* Lindl.** | | | | | | |
| 209 | ***Astragalus calycinus* M. Bieb.** | Luhansk region, Krasnodon, several locations: 48.28861°N, 39.77198°E; 48.29660°N, 39.78003°E; 48.29203°N, 39.77981°E; 48.28771°N, 39.77924°E | steppe areas with limestone outcrops | 05.05.2013 | N | (Yu. Peregrym et al., 2013);  POWO: ***A. calycinus*** |
| 210 | ***Astragalus visunicus* Kuczerevskij** | Mykolaiv region, Bereznehuvate district, between settlements Bereznehuvate and Vysuns’k, on the right bank of the Vysun River | steppes | 21.06.2001, 29.05.2003 | N | first described for science (Kucherevskyi, 2005);  POWO: ***A. visunicus*** |
| 211 | ***Cytisus polonicus* Sennikov et Val.N. Tikhom.** | 1) Lviv city, Holosko;  2) Lviv region, Lviv district, near Zhovkva town;  3) Lviv region, Zolochiv district, near Koltiv village, slopes of the Billa Hora hill;  4) Lviv region, Zolochiv district, the Lypytsia Mount near Shopky village;  5) Lviv region, Zolochiv district, Chervone village, Lysa Hora tract;  6) Ternopil region, Zalishchyky district, Kolodribka village;  7) Ivano-Frankivsk region, Tlumach district, Bratyshiv village near Nyzhniv village | in dry meadows or on calcareous denudations, on open slopes of hills and mountain foothills | 1) 08.05.1926, 15.05.1927;  2) 29.04 and 14.05.1914;  3) 22.05.1942;  4) 21.05.1988;  5) 19.05.1984;  6) 06.06.1984;  7) 22.04.1924 | N | first described for science (Sennikov & Tikhomirov, 2024a, 2024b);  POWO: ***-*** |
| 212 | ***Gleditsia triacanthos* L.** | Kyiv, M.M. Gryshko National Botanical Garden of the National Academy of Sciences of Ukraine | - | - | A | first cases of escape from cultivation (Shynder, 2019);  POWO: ***G. triacanthos*** |
| 213 | ***Hedysarum × smirnovii* Knjasev** (= *H. grandiflorum* Pall. × *H. biebersteinii* Žertova) | 1) Donetsk region, near Bakhmut;  2) Donetsk region, near the Sviatohirskyi monastery;  3) Luhansk region, Derkul experimental station, near Tretiyakove village;  4) Donetsk region, near Bohorodychne;  5) Luhansk region, Milove district, Striltsivskyi steppe;  6) Luhansk region, Slovyanoserbsk district, near Stakhanovka settlement;  7) Luhansk region, Lysychansk (now – Siverodonetsk) region, near Nyzhnie village | chalk slopes | 1) 22.06.1896;  2) 10.08.1912;  3) 17.05.1950;  4) 13.07.1951;  5) 24.05.1952;  6) 13.06.1973;  7) 13.06.1979 | N | first described for science (Knyasev, 2011);  POWO: ***H. × smirnovii*** |
|  | *Trifolium spryginii* Belyaeva & Sipliv. | Zhytomyr region, Zhytomyr district, 0.5 km to S from Ivanivka village | meadow-swamp vegetation | 13.08.2006 | N | (Orlov, 2008);  POWO: *T. lupinaster* L., already known for Ukraine (Mosyakin & Fedoronchuk, 1999) |
| ***Juglandaceae* DC. ex Perleb** | | | | | | |
|  | *Juglans ailantifolia* Carrière | Kyiv: 1) Feofania; 2) Metrolohichna Str., 12-b; 3) Akademika Lebedieva Str., 1; 4) Akademika Zabolotnoho Prospect, 156; 5) Right bank of Horikhuvatskyi Creek; 6) Syretskyi Park; 7) Syrestskyi Gai | park and forests | - | A | first cases of escape from cultivation (Burda & Koniakin, 2018); POWO: *J. mandshurica* var. *sachalinensis* (Komatsu) Kitam. |
| 214 | ***Juglans cinerea* L.** | 1) Kyiv, Right bank of Orekhovatrskii Creek;  2) Cherkasy region, Uman’ city, National Dendrological Park “Sofievka” of the NAS of Ukraine, Grekova Balka | park and forests | 1) - ;  2) 10.2017 | A | it was mentioned as a cultivated species (Mosyakin & Fedoronchuk, 1999), however several cases of escaping from its cultivation have been recently confirmed (Burda & Koniakin, 2018);  POWO: ***J. cinerea*** |
| 215 | ***Juglans mandshurica* Maxim.** | Kyiv: 1) the Syretskaya Grove; 2) Feofania; 3) Right bank of Orekhovatrskii Creek; 4) Syrestskaya Roshcha site  5) Donetsk, Donetsk Botanical Garden of the National Academy of Sciences of Ukraine | park and forests | 1) 1999;  2), 3), 4), 5) - | A | it was mentioned as a cultivated species (Mosyakin & Fedoronchuk, 1999), however several cases of escaping from its cultivation have been recently confirmed (Burda & Koniakin, 2018);  POWO: ***J. mandshurica*** |
| 216 | ***Juglans nigra* L.** | Kyiv: 1) Feofania; 2) Right bank of Orekhovatrskii Creek;  3) Dendrological Park “Olexandria” of the National Academy oZf Sciences of Ukraine;  4) Cherkasy region, Uman’ city, National Dendrological Park “Sofievka” of the National Academy of Sciences of Ukraine, Grekova Balka | park and forests | 1) 1999;  2), 3) - ;  4) 10.2017 | A | it was mentioned as a cultivated species (Mosyakin & Fedoronchuk, 1999), however several cases of escaping from its cultivation have been recently confirmed (Burda & Koniakin, 2018);  POWO: ***J. nigra*** |
|  | *Juglans subcordiformis* Dode | Kyiv: 1) Feofania; 2) Metrologicheskaya Str., 12-b; 3) Akademika Lebedeva Str., 1; 4) Akademika Zabolotnogo Prospect, 156; 5) Right bank of Orekhovatrskii Creek; | park and forests | - | A | first cases of escape from cultivation (Burda & Koniakin, 2018);  POWO: *J. mandshurica* var. *cordiformis* (Makino) Kitam. |
| ***Lamiaceae* Martinov** | | | | | | |
| 217 | ***Clinopodium caucasicum* Melnikov** | Transcarpathian Region, Uzhgorod city, village Hluboke, 20 km from the city | oak forest on a hill | 10.07.1958 | N | first described for science (Melnikov, 2013);  POWO: ***C. caucasicum*** |
| 218 | ***Galeopsis angustifolia* Ehrh. ex Hoffm.** | Zhytomyr region: 1) Olevsk district, Novi Bilokorovychi village, the railway station “Bilokorovychi”;  2) Korosten district, 2 km to S from the town, the railway station “Cholivka”;  3) Transcarpathian region, Chop, the railway station | 1) and 2) on the crushed stone between the tracks;  3) synanthropic communities | 1) 21.06.2016, 05.07.2016;  2) 26.07.2017;  3) 25.09. 2018 | A | (Orlov et al., 2019);  POWO: ***G. angustifolia*** |
| 219 | **Marrubium anisodon C. Koch** | Crimea: 1) Sudak;  2) Kurban-Kaya valley;  3) Sudak district, near Uyutne village | 1) and 2) -;  3) dry steppe ravine slope | 1) 20.07.1893, 24.06.1907;  2) 26.08.1926;  3) 11.08.1960 | A (?) | (Lazkov, 2011);  POWO: **M. anisodon** |
| 220 | ***Nepeta racemosa* Lam.** | Kyiv, M.M. Gryshko National Botanical Garden of the National Academy of Sciences of Ukraine | - | - | A | first cases of escape from cultivation (Shynder, 2019);  POWO: ***N. racemosa*** |
| 221 | ***Salvia revelata* Mátis & A.Z.Szabó** | Odesa region: 1) Illinka, 46.695175, 30.633318;  2) Illinka, 46.692993, 30.664467;  3) Nahirne, 45.465561, 28.436617;  4) Roksolany, 46.160131, 30.478081;  5) Pivdenne, 46.277749, 30.149614;  6) Sukhyi Lyman, 46.411445, 30.638949;  Mykolaiv region: 7) Mikhailovskaya steppe, 47.41983, 31.6248;  8) Velyke Artakove, 47.228333, 33.059667;  9) Mygiya, 48.011984, 30.979863;  10) Mykolaiv, 46.975033, 31.994583;  11) Kyriakivka, 47.028374, 31.845003;  12) Mykhailo-Laryne, 47.11206386, 32.20910289;  13) Barativka, 46.938057, 32.785337;  Kherson region: 14) Mala Oleksandrivka × Davydiv Brid, ravine Rusova, 47.268716, 33.240579;  15) Maryine, across river Ingulets, 47.477418, 33.344718;  16) Mylove, 47.054864, 33.580233;  17) Mylove, 47.082612, 33.640868;  Zaporizhzhia region: 18) Kalchynivka, 47.353057, 37.129435;  19) Balka Partiyanska, 47.8738889, 34.9747222;  20) Soniachne, 47.8905278, 35.0250833;  21) Soniachne, 47.870681, 34.973453;  Dnipropetrovsk region: 22) Matiuchenkove, 48.536615, 34.176329;  Kharkiv region: 13) Kumy, 49.344972, 35.389118 | petrophilous steppe habitats on loess and clay, between 40 and 120m altitude | 1) 14.05.2021; 2) 14.05.2021; 3) 17.05.2021; 4) 21.05.2021; 5) 22.05.2021; 6) 27.05.2021; 7) 25.04.2018; 8) 06.05.2018; 9) 16.05.2018; 10) 06.05.2017; 11) 13.07.2020; 12) 16.05.2021; 13) 24.05.2021; 14) 04.05.2018; 15) 05.05.2018; 16) 23.05.2021; 17) 03.06.2021; 18) 02.06.2002; 19) 01.05.2013; 20) 13.05.2016; 21) 20.05.2021; 22) 16.05.2009; 23) 13.05.2015 | N | first described for science (Mátis et al., 2023);  POWO: - |
| 222 | ***Thymus × goginae* Vasjukov** (= *Th. marschallianus* Willd. × *Th. ucrainicus* (Klokov et Des.-Shost.) Klokov.) | North part of Ukraine | on meadow slopes, forest glades and forest | - | N | first described for science (Vasjukov, 2016);  POWO: ***Th. × porcii* Borbás** |
| 223 | ***Thymus × pseudoalpestris* Ronniger ex Nachychko** (= *Th. alpestris* (Čelak.) A. Kern. × *Th. pulegioides* L.) | Ivano-Frankivsk region, Yaremche, Yavir mountain valley, 48°25´03.9´´N, 24°30´19.34´´E | among thick grass on the forest edge, 1124 m a.s.l. | 10.07.2012 | N | Validation of this name (Nachychko, 2016);  POWO: ***Th. × pseudoalpestris*** |
| 224 | ***Thymus alpestris* Tausch ex A.Kern × *T. pulcherrimus* Schur** | Transcarpathian region, Rakhiv district, urban type settlement Yasinia, rocky hollow above the lake Herashaska, N 48°16′19,9′′, E 24°09′50,6′′ | on the stones, 1640 m asl | 12.07. 2015 | N | first described for science  (Nachychko, 2014);  POWO: - |
| 225 | ***Thymus alternans* Klokov × *T. pulegioides* L.** | Transcarpathian region: 1) Rakhiv district, Kvasnyi tract; 2) Rakhiv district, Bohdan village, 48°01′49,3′′N, 24°21′25,7′′E; 3) Tiachiv district, Dubove village, south-west spur of Mount Apetska; 4) Khust district, Horinchovo village, the right bank of the Rika River;  5) Ivano-Frankivsk region, Kosiv district, near Rozhniv village, the banks of the Rybnytsia River;  6) Chernivtsi region, Kitsman district, Brusnytsia village, the second terrace of the Cheremosh River | 1) –;  2) southeastern outskirts, on the hayfield, 891 m a.s.l.;  3) on a meadow, in a grass stand;  4) floodplain;  5) on the river bank;  6) on the bank of the river | 1) –;  2) 12.06.1985;  3) 23.07.2010;  4) 31.05.1952;  5) 30.06.1966;  6) 20.06.1952 | N | first described for science  (Nachychko, 2014; Nachychko & Honcharenko, 2016);  POWO: - |
| ***Malvaceae* Juss.** | | | | | | |
| 226 | ***Hibiscus syriacus* L.** | 1) Cherkasy, 49.425286°N, 32.054533°E, spontaneous seedling;  2) Cherkasy region, Uman, 48.750379°N, 30.223615°E;  3) Chernivtsi, 48.287608°N, 25.936161°E, Cathedral of the Holy Spirit;  4) Kyiv, 50.41437°N, 30.561165°E, M.M. Gryshko National Botanical Garden;  5) Kyiv region, Bila Tserkva city, square near railway station, 49.811361°N, 30.108871°E;  6) Odesa city, 46.404526°N, 30.751991°E;  7) Ternopil, 49.562659°N, 25.594468°E;  8) Ternopil region, Chortkiv city;  9) Transcarpathian region, Mukachevo district, Svaliava, 48.549765°N, 22.982052°E, along central street;  10) Transcarpathian region, Berehove city, territory of B. Linner Berehove District Hospital, near central entrance, near place of culture;  11) Transcarpathian region, Berehove, 48.204664°N, 22.633318°E;  12) Transcarpathian region, Berehove, 48.217651°N, 22.645113°E;  13) Uzhhorod, Botanical Garden of Uzhhorod National University;  14) Uzhhorod, between Botanical Garden of Uzhhorod National University and Uzhhorod children railway | 1) near flower beds;  2) along edges of sidewalks in several places;  3) along sidewalk;  4) in cracks of paths and foundations of buildings;  5) in cracks along edge of sidewalk;  6) in flower beds and on sides of sidewalks and roads;  7) along edges of sidewalks;  8) beyond flower gardens;  9) in crack in sidewalk;  10) - ;  11) along walls and edges of sidewalks;  12) on roadside;  13) in undergrowth;  14) flower embankment | 1) 21.08.2022;  2) 01.07.2023;  3) 05.01.2023;  4) 12.04.2020, 29.09.2020;  5) 12.08.2023;  6) 26.09.2022;  7) 23.07.2023;  8) 1–15.07.2023;  9) 30.08.2022;  10) 28.08.2022;  11) 28.08.2022;  12) 01.09.2022;  13) 15.07.2022;  14) 16.09.2022 | A | The species was reported before only as a cultivated within Ukraine (Mosyakin & Fedoronchuk, 1999), so it is the first documented facts of its escaping (Raab-Straube & Raus, 2024);  POWO: ***H. syriacus*** |
| ***Menispermaceae* Juss.** | | | | | | |
| 227 | ***Menispermum dauricum* DC.** | Kyiv, M.M. Gryshko National Botanical Garden of the National Academy of Sciences of Ukraine | - | 2017 | A | first cases of escape from cultivation (Shynder, 2019);  POWO: ***M. dauricum*** |
| ***Onagraceae* Juss.** | | | | | | |
| 228 | ***Chamaenerion danielsii* (D. Löve) Czerep.** | Crimea: the Crimean Mountains and the South Coast | - | - | N | (Tikhomirov, 2015);  POWO: ***Epilobium angustifolium*** **L. subsp. *circumvagum* Mosquin** |
|  | *Epilobium menthoides* Boiss. et Heldr. | Crimea, Karadag near Koktebel | at the source | 29.08.1907 | N | (Tzvelev, 2007);  POWO: *E. parviflorum* Schreb, already known for Ukraine (Mosyakin & Fedoronchuk, 1999) |
|  | *Epilobium villosum* Thunb. | Southern Ukraine including Crimea | - | - | N | It is indicated that this species was previously considered as part of *E. hirsutum* L. (Tzvelev, 2007);  POWO: *E. hirsutum*, already known for Ukraine (Mosyakin & Fedoronchuk, 1999) |
| 229 | ***Oenothera × wienii* Renner ex Rostański** (= *Oe. depressa* Greene × *Oe. rubricaulis* Kleb.) | 1) Donetsk region, Yasynuvata town, about 500 m from the railway station “Yasynuvata- Pasazhyrska” (in the direction of Donetsk);  2) Kyiv, at the crossroads of Nauky Avenue and Kytaivska Street | 1) on the slope of the railway embankment;  2) near the railway crossing | 1) 16.07.1995;  2) 20.06.1996 | A | (Rostanski et al., 1997);  POWO: ***Oe.  × polgari* Rostański** |
|  | *Oenothera pycnocarpa* Atk. et Bartl. | Transcarpathian region, Uzhhorod, almost opposite the children's railway station "M. Gorky Park" | between the bank of the Uzh River and the railway track | 16.07.1995 | A | (Rostanski et al., 1997);  POWO: *Oe. biennis* L., already known for Ukraine (Mosyakin & Fedoronchuk, 1999) |
| 230 | ***Ludwigia brevipes* (B.H. Long ex Britton, A. Braun & Small) Eames** | Crimea, Sevastopol, coast of Striletska Bay, 44°35'37"N, 33°28'11"E | 1) 1 m, wasteland on ruins of ancient manor | 1) 01.09.2022, 04.09.2022, 20.09.2022, 28.09.2023 | A | (Raab-Straube & Raus, 2024)  POWO: ***L. brevipes*** |
| ***Orobanchaceae* Vent.** | | | | | | |
| 231 | ***Orobanche ritro* Gren. & Godr.** | Crimea, around Sevastopol, Mys Fiolent | - | 29.06.1981 | N | (Nobis et al., 2014);  POWO: ***O. centaurina* Bertol.** |
| 232 | ***Parentucellia latifolia* (L.) Caruel** | Crimea, Sevastopol, the top of the Kozacha bay, N 44°33’40” , E 33º24’30” | transformed limestone steppe, alt. 20 m | 18.04.2007 | N | (Seregin, 2009);  POWO: ***P. latifolia*** |
| 233 | ***Phelipanche schultzioides* M. J. Y. Foley** | Crimea, Karadag Reserve, Mt Sviata | rocky scree in *Fraxinus* forest, on *Symphytum tauricum* Willd. | 20.05.2014 | N | (Raab-Straube & Raus, 2017, p. 8);  POWO: ***Orobanche gussoneana* (Lojac.) Ined.** |
| ***Oxalidaceae* R.Br.** | | | | | | |
| 234 | ***Ionoxalis tetraphylla* (Cav.) J. Rose** | Zhytomyr region, Zhytomyr vicinity, Dovzhyk village, on the territory of "Zhytomyr Forestry", Bohun Forestry, quarter 62, section 18 | pine forest, along the path | summer 2019, 23.07.2020 | A | first cases of escape from cultivation (Orlov & Shevera, 2020);  POWO: ***Oxalis tetraphylla* Cav.** |
| 235 | ***Oxalis articulata* Savigny** | Crimea, vicinity of Yalta, Nikita village, 44°30'56"N, 34°14'14"E | 250 m, ruderal habitat on roadside | 07.06.2023 | A | the first case of escape from cultivation (Raab-Straube & Raus, 2024);  POWO: ***O. articulata*** |
| 236 | ***Oxalis latifolia* Kunth** | Crimea, Yalta vicinity, Nikita, Nikitsky Botanical Garden: 1) Lower Park, Palm Alley, 44°30'32"N, 34°14'02"E;  2) Upper Park, 44°30'37"N, 34°14'01"E;  3) 44°30'41"N, 34°13'56"E | 1) 100  m, regularly watered flower bed;  2) 135  m, flower bed under old tree of *Pistacia atlantica* Desf., among cultivated *Oxalis triangularis* subsp. *papilionacea* (Hoffmanns. ex Zucc.) Lourteig;  3) 150  m, regularly watered flower bed with planted chrysanthemums | 1) 26.06.2020;  2) 16.10.2020;  3) 19 & 23.10.2020 | A | first cases of escape from cultivation (Raab-Straube & Raus, 2021a);  POWO: ***O. latifolia*** |
| ***Paeoniaceae* Raf.** | | | | | | |
| 237 | ***Paeonia ×maleevii* Kem.-Nath. ex Mordak & Punina** (= *P. daurica* Jacks. × *P. tenuifolia* L.) | Mountain Crimea | - | XX century | N | first described for science (Punina et al., 2010);  POWO: ***P. × maleevii*** |
| ***Papaveraceae* Juss.** | | | | | | |
| 238 | ***Corydalis caucasica* DC.** | Kyiv, M.M. Gryshko National Botanical Garden of the National Academy of Sciences of Ukraine | - | - | A | first cases of escape from cultivation (Shynder, 2019);  POWO: ***C. caucasica*** |
| 239 | ***Fumaria capreolata* L.** | Crimea, between Miskhor and Alupka, near the cable car station built but not put into operation | ruderal vegetation with dominance of *Ambrosia artemisiifolia* L. | 17.05.2008, 30.05.2008 | A | (Bagrikova, 2009);  POWO: ***F. capreolata*** |
| 240 | ***Fumaria petteri* Rchb.** | Crimea, Sevastopol, near Balaklava, Inzhyr plateau | cliffs | 10.05.2015 | N | (Bondareva et al., 2018);  POWO: ***F. petteri*** |
| 241 | ***Hypecoum procumbens* L.** | Odesa, Khlibna harbor, near the grain elevator | near the wall of the house | 06.1994 - 1996 | A | The plants have not been observed again since 1996 (Vasyljeva & Kovalenko, 2000);  POWO: ***H. procumbens*** |
| 242 | ***Macleaya cordata* (Willd.) R. Br.** | 1) Zhytomyr, Shevchenko Str.;  2) Kyiv, O.V. Fomin Botanical Garden, on the site of the collection of medicinal plants and beyond;  3) Zhytomyr region, Korosten district, Hrozyne village, on the site of the collection of medicinal plants of a research institute of National Academy of Agricultural Sciences | 1) ruderal communities in the area between the fence and the sidewalk;  2) and 3) - | 1) 18.06.2007;  2) 24.08.2007;  3) 20.07.2008 | A | first cases of escape from cultivation (Orlov & Gubar, 2009);  POWO: ***M. cordata*** |
| 243 | ***Papaver atlanticum* (Ball) Coss.** | 1) Zhytomyr region, Berdychiv district, N vicinity of Ruzhyn town, 49.742867°N, 29.187592°E;  2) Kyiv, M.M. Gryshko National Botanical Garden of the National Academy of Sciences of Ukraine | 1) edge of wheat field;  2) beyond the borders of the flower gardens | 1) 26.06.2023;  2) 21.05.2022 | A | first cases of escape from cultivation (Raab-Straube & Raus, 2024)  POWO: ***P. atlanticum*** |
| 244 | ***Papaver minus* (Bél.) Meikle** | Crimea: 1) Crimean foothills and S coast, between Semydvirya and Kuru-Uzen [Soniachnohirske] village;  2) vicinity of Sudak;  3) vicinity of Sudak, W of Uyutne;  4) Karadag;  5) Novyi Svit;  6) between Morske and Rybache;  7) vicinity of Kurortne, Mt Echki-Dag;  8) vicinity of Morske, Mt Zerdale-Dere;  9) Bakhchysarai district, Belbek canyon;  10) E of Koktebel, Mt Kuchuk-Yanyshar;  11) E of Balaklava, Mt Spilia (Asketi);  12) Balaklava, Genoese fortress Cembalo;  13) vicinity of Koktebel, W spurs of Mt Biyuk-Yanyshar;  14) Sudak district, near Vesyoloye village, Mt Bash-Parmak;  15) Sudak district, Mt Papaya-Kaya;  16) Arabatska strilka spit | Stony slopes, gravel screes, sandy beaches | 1) 09.05.1924;  2) 09.05.1959;  3) 10.05.1977;  4) Apr–May 1963;  5) 15.05.1965;  6) 14.06.1982;  7) 29.05.1987;  8) 29.04.1988;  9) 19.06.1997;  10) 23.04.1999;  11) 21.05.2005;  12) 22.04.2006;  13) 17.05.2014;  14) 11.06.2014;  15) 27.06.2017;  16) 28.05.2016 | N | The occurrence of *P. argemone* L. in the Crimean Peninsula is doubtful, since almost all known records are referable in fact to *P. minus* (Bél.) Meikle (Raab-Straube & Raus, 2017, p. 8);  POWO: ***P. minus*** |
| 245 | ***Papaver tichomirovii* Mikheev** | Crimea | - | - | N | This species from the aggregate of *P. dubium* L. was described from Crimea in 1981, but validated in 1993 (Egorova, 1998), however it did not mention by Mosyakin & Fedoronchuk (Mosyakin & Fedoronchuk, 1999);  POWO: ***P. tichomirovii*** |
| ***Plantaginaceae* Juss.** | | | | | | |
| 246 | ***Plantago patagonica* Jacq.** | Transcarpathian region, Mukachevo district, Shkurativtsi village, southern slope of the volcanic ridge | pasture | 07.08.1951 | A | (Shipunov, 2000). The species had reported earlier (Shipunov, 1996), but it was not listed by Mosyakin & Fedoronchuk (Mosyakin & Fedoronchuk, 1999);  POWO: ***P. patagonica*** |
| 247 | ***Pseudolysimachion × blockianum* Trávn.** | Bilcze (Podole Galic.), on the walls of the Seret River | with *Veronica* *canescens* Schrad. | 1884 | N | (Travnicek, 1998). Now it is considered as *Veronica ×blockiana* (Trávn.) Albach (= *V. incana* L. × *V. spicata* L.) (Albach, 2008);  POWO: ***V. × blockiana*** |
| 248 | ***Psyllium sempervirens* (Crantz) Soj´ak** | Crimea, Yalta district, Nikita Botanical Garden | in the flowerbed | 3.04.1973 | A | (Shipunov, 1997);  POWO: ***Plantago sempervirens* Crantz** |
| 249 | ***Psyllium squalidum* (Salisb.) Soj´ak** | Kyiv region, Bila Tserkva, Dendropark “Olexandriia” | - | 19.09.1977 | A | (Shipunov, 1997);  POWO: ***Plantago afra* L. subsp. *afra*** |
| 250 | ***Veronica arguteserrata* Reg. et Schmalh.** | Odesa, near the humanitarian building of Odesa National University | on the flower bed, very sparse ruderal vegetation | 05.2006 | A | (Moysienko & Yena, 2006);  POWO: ***V. argute-serrata*** |
| 251 | ***Veronica catenata* Pennell** | Crimea, Sevastopol, Balaklava | - | 20.06.1893 | N | (Seregin, 2008);  POWO: ***V. catenata*** |
| ***Plumbaginaceae* Juss.** | | | | | | |
| 252 | ***Limonium* *sinuatum* (L.) Mill.** | Kyiv, Borshchahivska St., 50.447217ºN, 30.472772ºE | near the road | 06.08.2012 | A | accidentally introduced or escaped from its cultivation (Shynder et al., 2024);  POWO: ***L. sinuatum*** |
| ***Polygalaceae* Hoffmanns. & Link** | | | | | | |
| 253 | ***Polygala* × *kotovii* Val.N. Tikhom.** (= *P. comosa* Schkuhr × *P. cretacea* Kotov) | south-east of Ukraine | - | - | N | (Ostapko et al., 2010)  POWO: ***P.*× *kotovii*** |
| ***Polygonaceae* Juss.** | | | | | | |
| 254 | ***Aconogonon panjutinii* (Kharkev.) Soják** | Kyiv, M.M. Gryshko National Botanical Garden of the National Academy of Sciences of Ukraine | - | - | A | first cases of escape from cultivation (Shynder, 2019);  POWO: ***Koenigia panjutinii* (Kharkev.) T.M. Schust. & Reveal** |
| 255 | ***Calligonum aphyllum* (Pall.) Gürke** | Zaporizhzhia region, near Prymors’k | on the sands | 1906-1914 (?) | N(?) | (Shumilova, 2014);  POWO: ***C. aphyllum*** |
| 256 | ***Polygonum alpestre* C.A. Mey.** | Kherson, Canning plant | on the embankment between the sleepers of the railway track | 29.10.1999; 10.11.2000; 10.10.2003 | A | (Moysienko, 2005);  POWO: ***P. cognatum* Meisn. subsp. *cognatum*** |
| 257 | ***Rumex cristatus* DC. subsp. *cristatus*** | Crimea: 1) Sevastopol, near Tylove village  2) Sevastopol;  3) Balaklava  4) near Nikita Botanical Garden | 1) shore of the pond;  2) slopes along the railway;  3) along a path on a rocky slope;  4) along the road | 1) 16.08.1995;  2) 28.08.1995;  3) 29.08.1995;  4) 07.05.1996 | N (?) | (Yeremko, 1997);  POWO: ***R. cristatus* subsp. *cristatus*** |
| 258 | ***Rumex cristatus* DC. subsp. *kerneri* (Borbas) Akeroyd & D.A. Webb** | Crimea, Simferopol | - | 25.05.1897 | N (?) | (Yeremko, 1997);  POWO: ***R. kerneri Borbás*** |
| 259 | ***Rumex patientia* L. subsp. *patientia*** | Kyiv, M.M. Gryshko National Botanical Garden of the National Academy of Sciences of Ukraine | - | - | A | first cases of escape from cultivation (Shynder, 2019);  POWO: ***R. patientia* subsp. *patientia*** |
| ***Portulacaceae* Juss.** | | | | | | |
| 260 | ***Portulaca cypria* Danin** | Transcarpathian region, Tiachiv district, Teresva station, 47°59′45′′N, 23°41′59′′E | on the embankment of the narrow-gauge railway | 19.07.1946 | A | (Bulakh et al., 2019);  POWO: ***P. cypria*** |
| 261 | ***Portulaca daninii* Galasso, Banfi & Soldano** | 1) Chernivtsi region, Hlyboka district (now – Chernivtsi district), Hlyboka village;  2) Crimea, Sudak;  3) Crimea, Dzhankoi district, Tarkhan-Sunak;  4) Crimea, Feodosiya district, Vladislavovskyi site of grain farm, area between Novo-Pokrovka village and Seit-Asan village | 1) pasture, near the railway track;  2) S slope;  3) - ;  4) melon field | 1) 11.09.1953;  2) 31.07.1886;  3) 17.08.1897;  4) 22.08.1930 | A | It has been noted for the first time for Ukraine as *P. tuberculata* (Bulakh et al., 2020), but later - as *P. daninii* (Raab-Straube & Raus, 2023);  POWO: ***P. daninii*** |
| 262 | ***Portulaca granulatostellulata* (Poelln.) Ricceri & Arrigoni** | Transcarpathian region: 1) Berehove town, railway station, 48°12′9′′N, 22°38′15′′E;  2) Uzhhorod, Borodina Str., 48°36′34′′N, 22°18′02′′E | 1) between the plates;  2) in the cracks of the stone foundation of the fence | 1) 05.09.2017;  2) 06.10.2019 | A | (Bulakh et al., 2019);  POWO: ***P. granulatostellulata*** |
| 263 | ***Portulaca macrantha* (Maire) Ricceri & Arrigoni** | Crimea: 1) Sudak;  2) Balaklava;  3) Simeiz;  4) Ayu-Dag mountain;  5) Nikita, Nikitsky Botanical Garden, 44°30'41''N, 34°13'56''E;  6) Kherson region, Tsuriupinsk district (now – Kherson district), Kozachi Laheri;  7) Zhytomyr region, Zhytomyr district, Dovzhyk village;  8) Zhytomyr region, Zhytomyr district, Hlybochytsia village, side of Zhytomyr–Kyiv highway;  9) Zhytomyr region, Zhytomyr district, Barashivka village, private market garden  10) Zhytomyr, railway station;  11) Zhytomyr region, Zhytomyr district, Holubiatyn village, railway station;  12) Zhytomyr region, Zhytomyr district, Troshcha village, market garden;  13) Zhytomyr region, Zhytomyr district, Motovylivka village, side of highway | 1) S slope;  2) -;  3) in collibus;  4) SW slope;  5) 150 m, flowerbed;  6) sand;  7) weeds in market garden;  8) - ;  9) on hill;  10) unloading yard;  11) - ;  12) - ;  13) - | 1) 31.07.1886;  2) 1896;  3) 28 Jul 1903;  4) 22.08.1981;  5) 6.12.2019;  6) 19.07.1952;  7) 31.08.2019;  8) 18.09.2019;  9) 2.10.2019;  10) 8.10.2019;  11) 14.08.2021;  12) 20.08.2021;  13) 15.09.2021 | A | (Raab-Straube & Raus, 2023)  POWO: ***P. macrantha*** |
| 264 | ***Portulaca nitida* (Danin & H.G. Baker) Ricceri & Arrigoni** | Chernivtsi: 1) Central railway station;  2) Geroiv Maidanu Str., 41, the territory of the Chernivtsi base military hospital | 1) along the tracks;  2) lawn | 1) 24.06.2020;  2) 21.09.2020 | A | (Bulakh et al., 2020);  POWO: ***P. nitida*** |
| 265 | ***Portulaca papillatostellulata* (Danin & H.G. Baker) Danin** | Transcarpathian region: 1) Svalyava district, Holubyne village, the road to the sanatorium “Kvitka polonyny”, 48°35′56′′N, 22°58′24′′E;  2) Volovets town, railway station, 48°42′48′′N, 23°00′03′′E | 1) along the road;  2) between the tracks | 1) 25.09.2017;  2) 18.09.2019 | A | (Bulakh et al., 2019);  POWO: ***P. papillatostellulata*** |
| 266 | ***Portulaca rausii* Danin** | Odessa Region, Ovidiopol district, NE part of Karolino-Bugaz village, near platform of railway station Studentska, 46°09′53.11″N, 30°33′24.87″E | at railway crossing | 28.08.2012 | A | (Raab-Straube & Raus, 2015, p. 5);  POWO: ***P. rausii*** |
| 267 | ***Portulaca sardoa* Danin, Bagella & Marrosu** | 1) Crimea, Yalta, bus station, 44°30'42''N, 34°10'14''E, 44°30'41''N, 34°10'11''E;  2) Zhytomyr, centre, near Zhytomyr Hotel;  Zhytomyr region: 3) Zhytomyr district, Teteriwka village, market garden;  4) Zhytomyr district, Radomyshl, private sector, market garden;  5) Berdychiv, railway station;  6) Zhytomyr district, Troshcha village, market garden;  7) Zhytomyr district, Velyka Volytsia village;  8) Zhytomyr district, Velyki Korovyntsi village, railway station;  9) Berdychiv district, Derganivka village, railway station | 1) 45 m, flowerbed;  2) flowerbed;  3) - ;  4) - ;  5) near the track;  6) - ;  7) wet clay on shore of pond;  8) gravel;  9) - | 1) 22.12.2019, 28.09.2021;  2) 18.09.2019;  3) 6.10.2019;  4) 9.08.2020;  5) 24.07.2021;  6) 20.08.2021;  7) 16.09.2021;  8) 19.09.2021;  9) 9.10.2021 | A | (Raab-Straube & Raus, 2023)  POWO: ***P. sardoa*** |
| 268 | ***Portulaca socotrana* Domina & Raimondo** | Crimea, Hurzuf, Podvoyskoho str., 44°32'38''N, 34°16'48''E | 50 m | 2.10.2021 | A | (Raab-Straube & Raus, 2023)  POWO: ***P. socotrana*** |
| 269 | ***Portulaca trituberculata* Danin, Domina & Raimondo** | Chernivtsi region, Hlyboka district (now – Chernivtsi district), Hlyboka village | pasture, near the railway track | 11.09.1953 | A | (Bulakh et al., 2020);  POWO: ***P. trituberculata*** |
| ***Ranunculaceae* Juss.** | | | | | | |
| 270 | ***Clematis serratifolia* Rehder** | Chernihiv region, Pryluky district, Trostianets village: 1) surroundings of Trostianets State Dendrological Park, 50.78810819°N, 32.8046822°E;  2) Trostianets State Dendrological Park, 50.788390°N, 32.805177°E | - | 1) 01.10.2022;  2) 01.10.2022 | A | first cases of escape from cultivation (Raab-Straube & Raus, 2024);  POWO: |
| 271 | ***Helleborus caucasicus* A.Braun** | Kyiv, M.M. Gryshko National Botanical Garden of the National Academy of Sciences of Ukraine | - | - | A | first cases of escape from cultivation (Shynder, 2019);  POWO: ***Helleborus orientalis* Lam. subsp. *orientalis*** |
| ***Rosaceae* Juss.** | | | | | | |
| 272 | ***Cotoneaster horizontalis* Decne** | Crimea, S outskirts of Sevastopol, vicinity of Lermontov Cape, tourist hostel, 44°30′50″N, 33°28′40″E | disturbed edge of seashore escarp; alt. 90 m | 11.09.2003 | A | relic of former cultivation (Seregin, 2008);  POWO: ***C. horizontalis*** |
| 273 | ***Cotoneaster lucidus* Schltdl.** | Kyiv, M.M. Gryshko National Botanical Garden of the National Academy of Sciences of Ukraine | - | - | A | first cases of escape from cultivation (Shynder, 2019);  POWO: ***C. acutifolius* Turcz.** |
| 274 | ***Cotoneaster multiflorus* Bunge** | Kyiv, M.M. Gryshko National Botanical Garden of the National Academy of Sciences of Ukraine, 50.40522, 30.56055 | - | 19.05.2011, 24.06.2020, | A | first cases of escape from cultivation (Shynder, Doiko, et al., 2022);  POWO: ***C. multiflorus*** |
| 275 | ***Crataegus* × *dunensis* Cin.** (= *C. lindmanii* Hrab.-Uhrova × *C. rhipidophylla* Gand.) | Zhytomyr region, Luhyny district, 0.5 km to SW from Rudnia-Povchanska village, blok #48 of the Povchans’ke Forestry | a glade in the middle of a pine forest | 2005, 2006 | N | (Fitsajlo & Orlov, 2009);  POWO: ***C.*×*dunensis*** (an unplaced name) |
| 276 | ***Crataegus* × *poplavskae* Tzvelev** (= *С. microphylla* C. Koch × *C. stevenii* Pojark.) | Crimea, Yalta Mountain-Forest Nature Reserve | in forest | 28.08.1925 | N | first described for science (Tzvelev, 2001b);  POWO: ***C.*× *poplavskae*** (an unplaced name) |
| 277 | ***Crataegus monogyna* Jacq. × *C. laevigata* (Pior.) DC. × *C. rhipidophylla* Gand.** | Khmelnytskyi region, National Nature Park “Podilski Tovtry”, Nihyn – N part, N 48°50'47'' Eº26°33'42" | - | 2013 | N | (Sołtys-Lelek & Oliіar, 2016);  POWO: - |
|  | *Malus niedzwetzkyana* Dieck | Zhytomyr region, E vicinity of Berdychiv city, 49.89703°N, 28.6577°E, edge of Trykutnyk forest | near highway | 21.06.2021 | A | the first documented case of its escaping from cultivation (Raab-Straube & Raus, 2024)  POWO: *M. domestica* (Suckow) Borkh. (the cases of the species escaping are already known for Ukraine (Mosyakin & Fedoronchuk, 1999)) |
| 278 | ***Malus toringo* (Siebold) de Vriese** | 1) Kirovohrad region, Holovanivsk district, Novoselytsia village, 48.303917°N, 30.326258°E;  2) Kyiv, M.M. Gryshko National Botanical Garden, in Syringaria plot, 50.417317°N, 30.563095°E | 1) -  2) edge of path | 1) 29.06.2021;  2) 30.06.2022 | A | first cases of escape from cultivation (Raab-Straube & Raus, 2024);  POWO: ***M. toringo*** |
| 279 | ***Potentilla virgata* Lehm.** | Kherson, on the territory of the cotton mill | on the railway among the sleepers | 07.08.1997 | A | (Moysienko, 1998);  POWO: ***P. virgata*** |
| 280 | ***Pyracantha rogersiana* (A.B. Jacks.) Coltm.-Rog.** | Crimea, Sevastopol, Strilets’ka Balka, N 44°35’30”, E 33º28’20” | tree-shrub thickets along the thalweg, alt. 5 m | 23.08.2008 | A | first cases of escape from cultivation (Seregin, 2010);  POWO: ***P. crenulata* (D.Don) M.Roem.** |
| 281 | ***Rosa gallica* L.** | Crimea, Sevastopol: 1) Baidarska valley, between the Chorna River and Urkukdusy [Urkusta];  2) near Baydarski Vorota pass;  3) Sevastopol, Mount Sapun-Gora;  4) Cape Aya reserve, Ayazma locality | 1) -;  2) open slope;  3) SE slope, limestone;  4) stony W slope, *Pinus stankewiczii* (Sukacz.) Fomin forest, alt. 200–300 m | 1) 18.06.1906;  2) 21.06.1912;  3) 16.06.1982, 26.07.1984;  4) 01.06.1985 | N | (Seregin, 2008);  POWO: ***R. gallica*** |
| 282 | ***Rubus austroslovacus* Trávn.** | Transcarpathia region: 1) near Serednie;  2) Mt. Nagyhegy near Ardó (today part of Berehove) | 1) in coppices, alt. 150 m a.s.l;  2) on the mountainside | 1) 17.07.1935;  2) 25.07.1935 | N | (Nobis et al., 2020);  POWO: ***R. austroslovacus*** |
| 283 | ***Rubus bertramii* G. Braun** | 1) Lviv region, Zolochiv district, near Chervone village, to E from the Lysa Mount  2) Rivne region, Rivne forestry | 1) on a felling area in a beech forest;  2) deciduous forest | 1) 14.07.1996;  2) 21.08.1997 | N | (Krassovskaja et al., 2001);  POWO: ***R. bertramii*** |
| 284 | ***Rubus bicolor* Opiz** | Transcarpathian region, Mukachevo: 1) Chervona Hora;  2) Mt Lovachka | 1) - ;  2) - | 1) July 1917;  2) 08.07.1935 | N | (Dudáš et al., 2022, p. 9);  POWO: ***R. bicolor*** |
| 285 | ***Rubus crispomarginatus* Holub** | Transcarpathia region, near Velikyi Bereznyi | in forests, alt. 150 m a.s.l | 18.06.1935 | N | (Nobis et al., 2020);  POWO: ***R. crispomarginatus*** |
| 286 | ***Rubus fasciculatus* P.J. Müll.** | Transcarpathian region, Berehove district, “In valle Czerni” | forest edge | 08.1912 | N | (Krassovskaja, 2008);  POWO: ***R. fasciculatus*** |
| 287 | ***Rubus gothicus* Frid. et Gelert ex E.H.L. Krause** | Transcarpathian region, Berehove district, Muzhiyevo village | along the road | 30.05.1990 | N | (Honcharenko, 2000);  POWO: ***R. gothicus*** |
| 288 | ***Rubus haesitans* Martensen et Walsemann** | Transcarpathian region, Berehove district, Muzhivo village, the Kukla Banya Mount | on the slope | 29.05.1990 | N | (Honcharenko, 2000);  POWO: ***R. haesitans*** |
| 289 | ***Rubus laciniatus* Willd.** | Kyiv region, Borodianka district, near the Makiichukove railway station, 50 m from the platform | in 50-year-old pine artificial forest | 2012 | A | (Melnyk et al., 2013);  POWO: ***R. nemoralis* P.J.Müll.** |
| 290 | ***Rubus perperus* H.E. Weber** | Transcarpathian region, Mukachevo, Pidhoriany district | - | 25.07.1928 | N | (Dudáš et al., 2022, p. 9);  POWO: ***R. perperus*** |
| 291 | ***Rubus orthostachys* G. Braun** | Chernivtsi region, Storozhynets district, near Chervona Dibrova village | on the edge of the broadleaf forest | 18.07.1996 | N | (Krassovskaya, 1998);  POWO: ***R. orthostachys*** |
| 292 | ***Rubus wimmerianus* (Sprib. ex Sudre) Sprib** | S of Lviv | - | - | N | (Zielinski, 2004). The species was noted for Transcarpathian region (near Mukachevo) later based on herbarium data from Budapest (Krassovskaja, 2008);  POWO: ***R. wimmerianus*** |
| 293 | ***Sorbus × hybrida* L.** | Kyiv, M.M. Gryshko National Botanical Garden of the National Academy of Sciences of Ukraine | - | - | A | first cases of escape from cultivation (Shynder, 2019);  POWO: ***Hedlundia hybrida* (L.) Sennikov & Kurtto** |
| ***Rubiaceae* Juss.** | | | | | | |
| 294 | ***Galium divaricatum* Pourr. ex Lam.** | Transcarpathian region, Irshava district, near the village of Siltse, the upper edge of the quarry near a footpath to the hilltop (48°17'36.1"N, 22°59'17.6"E) | open grassland containing mainly thermophilous acidotolerant therophytes: *Sedo-Scleranthetea* Br.-Bl. 1955. | 10.06.2016 | N | (Novák & Zukal, 2018);  POWO: ***G. divaricatum*** |
| 295 | ***Galium × jubilaeare* Ostapko** (= *G. cincinnatum* (Klokov) Ostapko × *G. ruthenicum* Willd.) | Donetsk, Shyrokyi sub-district, the left bank, Pishchanyi pond, 47°56ʹ43,88ʺN, 37°44ʹ53,79ʺE | steppe slope, 148 m a.s.l. | 03.07.2020 | N | first described for science (Ostapko, 2020);  POWO: no data |
| ***Santalaceae* R.Br.** | | | | | | |
| 296 | ***Thesium krymense* Romo, Didukh & Boratyński** | Crimea: 1) the Bila Skelia Mount;  2) Sevastopol, mount Sapun;  3) Bakhchysarai district, Malo-Sadove;  4) Bakhchysarai district, Krasnyi Mak;  5) Yalta district, Hursuf, Mertva dolyna;  6) Buyurlun, near Hurzuf | 1) dry grasslands in eroded margaceous soil;  2) northern slope;  3) calcareous rocks;  4) mountain summit;  5) calcareous shale;  6) calcareous shale | 1) 07.06.2001;  2) and 3) 1981;  4) 1997;  5) 1929;  6) 1930 | N | first described for science (Romo et al., 2004);  POWO: ***Th. krymense*** |
| ***Sapindaceae* Juss.** | | | | | | |
| 297 | ***Acer monspessulanum* L.** | 1) Kirovohrad region, Dolyna Community, N vicinity of Ivanivka village, 48.20802°N, 32.85166°E;  2) Mykolaiv region, vicinity of Trykraty village, Trykraty forest;  3) Odesa city, 46.384287°N, 30.74988°E; 46.384357°N, 30.749866°E, | 1) roadside;  2) - ;  3) lower part of slope above sea; on both sides of road | 1) 07.10.2022;  2) 03.05.2020;  3) 26.09.2022 | A | first cases of escape from cultivation (Raab-Straube & Raus, 2024);  POWO: ***A. monspessulanum*** |
| 298 | ***Koelreuteria paniculata* Laxm.** | Crimea, Sevastopol, Strilets’ka Balka, N 44°35’30”, E 33º28’20” | tree-shrub thickets along the thalweg, alt. 5 m | 23.08.2008 | A | first cases of escape from cultivation (Seregin, 2010);  POWO: ***K. paniculata*** |
| ***Solanaceae* Juss.** | | | | | | |
| 299 | ***Datura ferox* L.** | 1) Odesa  2) Dnipropetrovsk region, Kryvyi Rih, Kryvyi Rih Botanical Garden, 48.15130ºN, 33.57996ºE; and 48.15116º N, 33.58068º E | 1)-  2) botanical garden, alt. 111 m and in a protective forest strip, alt. 111 m | 1)-;  2) 25.07.2022; | A | The plant was noted from Odesa city as ephemerophyte for the first time (Petryk, 1992), but a real fact of its spreading has been only recently confirmed in Kryvyi Rih Botanical Garden (Moysiyenko et al., 2023);  POWO: ***D. ferox*** |
| 300 | ***Datura wrightii* Regel** | Crimea, Sevastopol, between Stiletska Bay and Park “Pobedy [Peremohy]”, the corner of Admiral Fadieiev Str., 44°35ʹ50ʺN, 33°27ʹ45ʺE | weedy place | 29.07.2014 | A | (Mayorov, 2018);  POWO: ***D. wrightii*** |
| 301 | ***Physalis peruviana* L.** | 1) Cherkasy region, vicinity of Uman;  2) Kyiv region, Vasylkiv | 1) near random dump on edge of forest  2) abandoned railway track | 1) 21.10.2018;  2) 20.10.2020 | A | The species was mentioned as a cultivated one (Mosyakin & Fedoronchuk, 1999), the first cases of its escape from cultivation are reported here (Raab-Straube & Raus, 2024)  POWO: ***P. peruviana*** |
| 302 | ***Physalis philadelphica* Lam. subsp. *ixocarpa* (Brot. ex Hornem.) Sobr.-Vesp. & Sanz-Elorza** | Kyiv, M.M. Gryshko National Botanical Garden of the National Academy of Sciences of Ukraine | - | 09.09.2021 | A | first cases of escape from cultivation (Shynder, Doiko, et al., 2022);  POWO: ***P. ixocarpa* Brot. ex Hornem.** |
| 303 | ***Solanum retroflexum* Dunal** | Odesa region, Danube Biosphere Reserve, the Ptashyna spit located to the right of the Bystre mouth; and the Yermakov’s Island | areas with transformed natural vegetation, or coastal and channel neoplasms | 19.07.2005 | A | (Zhmud & Zhmud, 2010);  POWO: ***S. retroflexum*** |
| ***Talinaceae* (Fenzl) Doweld** | | | | | | |
| 304 | ***Talinum paniculatum* (Jacq.) Gaertn.** | Kyiv, Symon Petlura Str., 1, O.V. Fomin Botanical Garden of the Taras Shevchenko National University of Kyiv | - | - | A | first cases of escape from cultivation (Shynder, Kolomiychuk, et al., 2022);  POWO: ***T. paniculatum*** |
| ***Tamaricaceae* Link** | | | | | | |
| 305 | ***Tamarix laxa* Willd.** | 1) Donetsk region, Mariupol district, Mangush settlement territorial community, Bilosarayska Kosa village  2) Crimea, Kerch Peninsula, near Uvarove village, 45.30049°N, 35.63050°E | 1) *Tamariceta gracilis*  2) - | 1) 08.05.2016  2)06.05.2023 | N | (Bronskov & Bronskova, 2024)  POWO: ***T. laxa*** |
| ***Violaceae* Batsch** | | | | | | |
| 306 | ***Viola* *×bachtschisaraensis* Vl. Nikit.** (= *V. dehnhardtii* Tenore × *V. sieheana* W.Becker) | Crimea, near Bakhchysarai town, above Sokolyne village | in a deciduous forest, h = 800 m above s.l. | 12.04.1989 | N | first described for science (Nikitin, 2001);  POWO: ***V. × bachtschisaraensis*** |
| 307 | ***Viola hissarica* Juz.** | 1) Dnipro, 48.4525°N, 35.0626°E, campus of Oles Honchar Dnipro National University,  Gagarina Avenue;  2) Dnipro, Olesya Honchara Street, 48.4513°N,  35.0560°E; 48.4510°N, 35.0558°E;  3) Dnipro, Kniahyni Olhy Street, 48.4755°N, 35.0252°E;  4) Odesa, Prymorska Street, 46.4864°N, 30.7430°E;  5) Odesa. Gogolya Street;  6) Odesa, Panteleimonivska Street;  7) Zaporizhzhia, Zaporizhzhia City Children Botanical Garden, administrative building and greenhouses;  8) Odesa city, Botanical Garden of I.I. Mechnykov Odesa National University;  9) Kyiv, Arboretum of M.M. Gryshko National Botanical Garden | 1) on lawn;  2) near residential building, ornamental garden area;  3) near residential  building;  4) ornamental garden area;  5) near flower bed, along cracks in sidewalk;  6) around parking lot, cracks in road surface;  7) along cracks of paths;  8) in flower bed;  9) in shade | 1) 24.04.2012;  2) 09.04.2017;  3) 25.03.2020;  4) 15.10.2021;  5) 16.09.2022, 21.04.2023;  6) 21.04.2023;  7) 01.06.2023;  8) 16.09.2022;  9) 16.04.2023, 21.06.2023 | A | first cases of escape from cultivation (Raab-Straube & Raus, 2024);  POWO: ***V. hissarica*** |
| 308 | ***Viola* *× poltavensis* Vl. Nikit.** (= *V. montana* L. × *V. nemoralis* Kütz.) | Poltava region, Pryluky district, near Irzhavets village (now – Chernihiv region) | slopes of ravines with oaks | 30.07.1916, 27.07.1917 | N | first described for science (Nikitin, 2001);  POWO: ***V. nemoralis* subsp. *nemoralis*** |
| 309 | ***Viola* × *popovae V*l. Nikit. nothosubsp. *romankoshica* Vl. Nikit.** (=*V. nemoralis* Kijrtz. subsp. *abbreviata* Vl. Nikit. × *V. sieheana* W. Beck.) | Crimea, on the top of the Rom-Kosh Mount | on rocks | 25.07.1962 | N | first described for science (Nikitin, 2003);  POWO: this species (***V.*×*popovae***) is accepted, but this nothosubspecies is not mentioned |
| 310 | ***Viola sororia* Willd.** | Kyiv, M.M. Gryshko National Botanical Garden of the National Academy of Sciences of Ukraine | - | - | A | first cases of escape from cultivation (Shynder, 2019);  POWO: ***V. sororia*** |
| 311 | ***Viola vadimii* Vl. Nikit.** | Don Oblast’, along the Donets, near Gundorovskaya village, Popov’s farm (now – Luhansk region, Sorokine district, near Popivka village) | chalk slopes | 26.06.1888 | N | first described for science (Nikitin, 2002);  POWO: ***V. vadimii*** |
| ***Vitaceae* Juss.** | | | | | | |
| 312 | ***Ampelopsis aconitifolia* Bunge** | 1) Odesa, Botanical Garden of I.I. Mechnykov Odesa National University;  2) Uzhhorod, Botanical Garden of Uzhhorod National University;  3) Donetsk, Donetsk Botanical Garden;  4) Dnipropetrovsk region, Kryvyi Rih, Botanical Garden;  5) Kyiv, M.M. Grysko Botanical Garden;  6) Cherkasy region, [Uman], Sofiivka National Dendrological Park;  7) Chernihiv region, Trostianets State Dendrological Park | - | 1) 16.09.2022;  2) 05.09.2022;  3) -  4) 08.10.2022;  5) 2016–2023;  6) and 7) – | A | first cases of escape from cultivation (Raab-Straube & Raus, 2024);  POWO: ***A. aconitifolia*** |
| 313 | ***Vitis × instabilis* Ardenghi, Galasso, Banfi & Lastrucci** | 1) Mykolaiv region, Mykolaiv district, 47.380441°N, 31.628250°E, Natural Reserve Yelanetskyi Step, Mykhaylivske division, Kemlych ravine;  2) Odesa region, Bilhorod-Dnistrovskyi district, N vicinity of Kozatske village, 46.371549°N, 30.084136°E | 1) carbonate shrub-steppe slope;  2) bank of canal | 1) 09.10.2022;  2) 23.09.2022 | A | first cases of escape from cultivation (Raab-Straube & Raus, 2024);  POWO: ***V. × instabilis*** |
| 314 | ***Vitis riparia* Michx.** | Kyiv: 1) M.M. Gryshko National Botanical garden and its surroundings: 50.410119ºN, 30.558811ºE; 50.429861ºN, 30.568471ºE; 50.422466ºN, 30.567653ºE; 50.415342ºN, 30.555466ºN;  2) Vydubychi, 50.402652ºN, 30.553323ºE;  3) Embankment of the Dnipro River, 50.431512ºN, 30.567519ºE;  4) next to the Syrets dendropark, 50.484876ºN, 30.422088ºE;  5) Cherkasy region, Kaniv, the right bank of the Dnipro Riever, to east from the bus station;  6) Cherkasy region, Cherkasy district, the west border of Bilozirya village, Bile Ozero tract;  7) Zhytomyr region, Berdychiv, southern outskirts, Chervona Hora tract, the right bank of the Hnylopyat river, 49.880871°N, 28.576423°E | 1) – ;  2) along the railway embankments;  3) along the river;  4) in the valley;  5) spontaneously in a coastal forest;  6) dry meadows on the sand;  7) along the edge of the forest, on tall shrubs; | 1) 26.09.2014; 15.09.2020; 12.10.2020; 2.11.2021;  2) 14.06.2021;  3) 31.05.2020;  4) 1.11.2021;  5) 11.08.2019;  6) 11.08.2018;  7) 24.07.2021; | A | It was mentioned as a cultivated species (Mosyakin & Fedoronchuk, 1999), however many cases of escaping from its cultivation have been recently confirmed (Orlov et al., 2022; Shynder, Doiko, et al., 2022; Shynder, Kostruba, et al., 2022);  POWO: ***V. riparia*** |
